# Interfacial epitaxy of single-crystalline Al_2_(MoO_4_)_3_ flakes for anisotropic phonon polaritons

**DOI:** 10.1038/s41467-026-74681-x

**Published:** 2026-06-25

**Authors:** Chuangye Song, Jiawei Huang, Song Zhou, Yuanhao Lyu, Xiaoyue He, Shaoxiang Sheng, Kehui Wu

**Affiliations:** 1 Tsientang Institute for Advanced Study, Hangzhou, China; 2 School of Mathematics and Physics, North China Electric Power University, Beijing, China; 3 Songshan Lake Materials Laboratory, Dongguan, China

**Keywords:** Optical materials and structures, Materials for optics, Techniques and instrumentation

## Abstract

The unique phonon characteristics of metal molybdates give rise to intriguing anisotropies in thermal conductivity and mechanical elasticity. However, direct nanoscale imaging of these phonon modes and their interactions with light remains challenging, as these materials have so far been available only as isomorphic polycrystalline films. Here, we develop a cost-effective interfacial epitaxy strategy for synthesizing thin-layer aluminum molybdate (Al_2_(MoO_4_)_3_, AMO) single crystals. This is achieved by confining a molybdenum foil between two sapphire substrates, which directs uniform nucleation and epitaxial growth of high-quality monoclinic AMO. The strong phonon-photon coupling fosters hyperbolic phonon polaritons (PhPs), whose propagation can be tailored by excitation frequency and crystal symmetry. Crucially, biaxial epitaxial strain induces anisotropic edge-launched PhPs. This work establishes a general approach for synthesizing high-quality ternary metal molybdates, providing a versatile platform for polaritonics and nanophotonics.

## Abstract (web summary)

Metal molybdates exhibit interesting anisotropic thermal and mechanical properties, but the synthesis of single-crystalline films and the characterization of their light-matter interactions remain challenging. Here, the authors report the synthesis of single-crystalline Al_2_(MoO_4_)_3_ hosting hyperbolic phonon polaritons in the mid-infrared.

## Introduction

Ternary metal molybdates (A₂(MoO₄)₃, A = Al³⁺, Fe³⁺, Cr³⁺, etc.) have garnered significant interest due to their exceptional properties, such as negative thermal expansion^1^, ferroelastic phase transitions^2,3^, elastic anisotropy^4^, and pressure-induced amorphization^5^. These compounds typically crystallize in a monoclinic structure (space group *P2*_*1*_*/a*), comprising corner-sharing AO₆ octahedra and MoO₄ tetrahedra interconnected via A–O–Mo bonds^6^. This distinctive lattice framework is key to their pronounced anisotropies in thermal conductivity and mechanical elasticity. Notably, the strong covalent bonding within the MoO₄ tetrahedra gives rise to intense optical phonon modes^7–9^, which facilitate efficient photon-phonon resonance absorption^10,11^. Furthermore, these phonons can strongly couple with electromagnetic radiation, supporting hyperbolic phonon polaritons (PhPs) that confine light at deep subwavelength scales^12–15^.

Investigating the propagation and properties of these PhPs necessitates well-defined crystal structures with minimal scattering from grain boundaries and defects^16,17^. However, studies on A₂(MoO₄)₃ have so far been constrained to isomorphic polycrystals^18,19^, where structural disorder obscures intrinsic phonon characteristics and impedes direct observation of PhPs. Conventional synthetic routes, primarily solid-state reactions between A₂O₃ and MoO₃ at 800–1000 °C^2,20,21^, typically yield polycrystalline products. A significant challenge is that these temperatures exceed the sublimation point of MoO₃^22,23^, preventing the growth of high-purity, single-crystalline phases and thus limiting their functional performance. The synthesis of high-quality, single-crystalline A₂(MoO₄)₃ could provide an additional material platform for exploring anisotropic polaritonic phenomena and prospective optoelectronic applications.

In this work, we develop an efficient and economical interfacial epitaxy strategy to synthesize single-crystalline Al₂(MoO₄)₃ (AMO) flakes with controllable dimensions. Our approach involves sandwiching a molybdenum foil between two sapphire wafers and heating the assembly in air. This configuration creates a confined space where sublimated molybdenum oxide vapors react with the sapphire surfaces, promoting uniform nucleation and epitaxial growth of AMO. Comprehensive characterization by Raman spectroscopy, X-ray diffraction (XRD), and scanning transmission electron microscopy (STEM) confirms the high crystallinity and reveals the presence of biaxial epitaxial strain. Moreover, photo-induced force microscopy (PiFM) measurements demonstrate strong phonon-polariton coupling, with hyperbolic interference patterns that clearly showcase in-plane anisotropy.

## Results

### Synthesis of AMO crystals via interfacial epitaxy

**Fig. 1:**
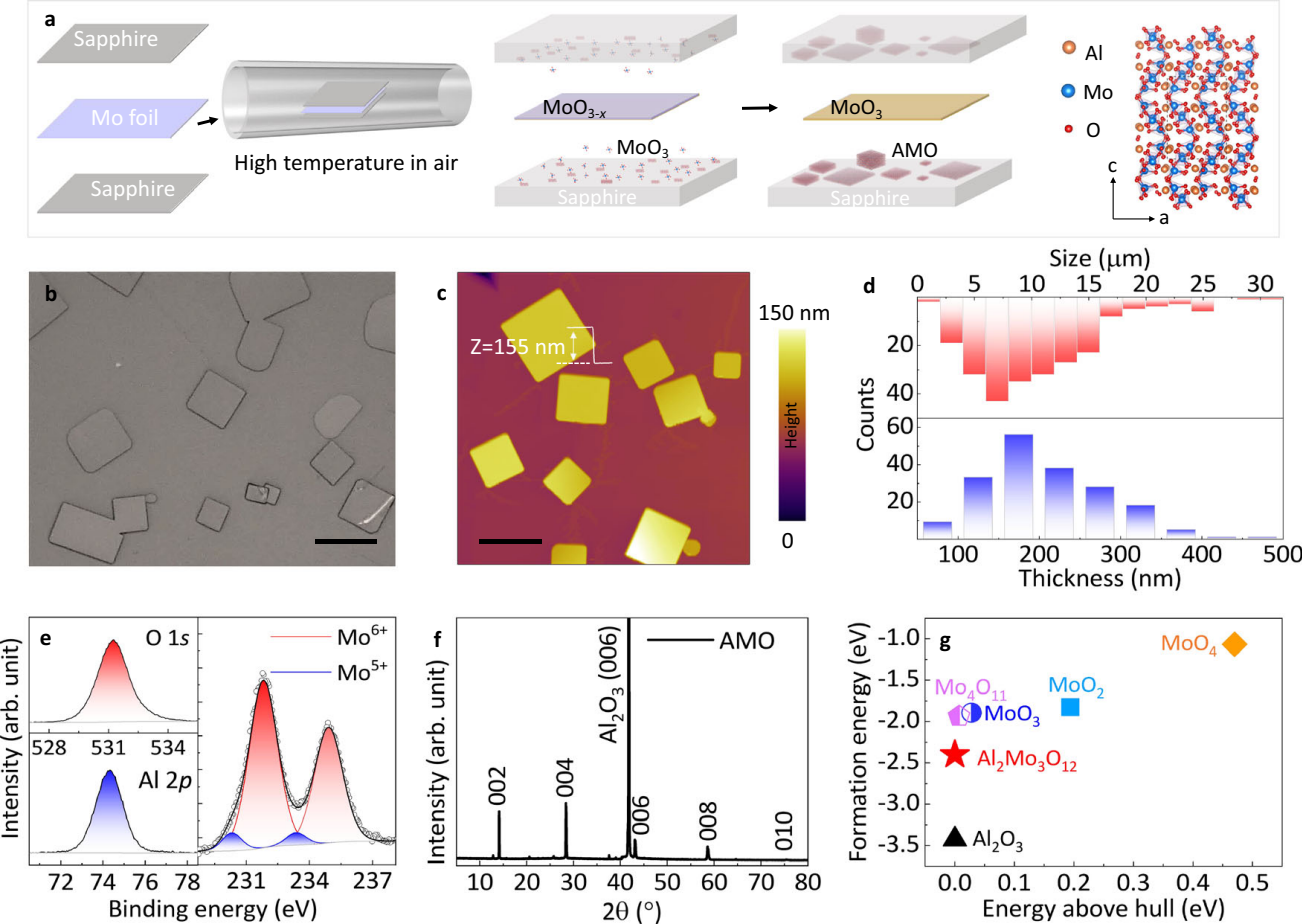
Interfacial epitaxy of Al_2_(MoO_4_)_3_ (AMO) flakes. **a** Left panel: Schematic illustration of space-confined synthetic process of AMO with the sandwich structure precursor. Right panel: the proposed growth mechanism of AMO flakes. Crystal structure of monoclinic AMO (*P2*_*1*_*/a*, *a* = 9.0 Å, *b* = 9.1 Å, *c* = 15.49 Å, *β* = 125.3°) is presented, the blue, orange, and red spheres representing molybdenum, aluminum, and oxygen atoms, respectively. MoO_4_ tetrahedron are highlighted in pink. Throughout this work, the *a*-, *b*-, and *c*-axes are defined to be parallel to the [100], [010], and [001] crystallographic directions, respectively. **b** Optical microscope image of the as-grown AMO flakes on a sapphire substrate. **c** Atomic force microscope (AFM) image of the as-grown AMO flakes. Inset is the corresponding height profile. **d** Distribution of the thickness (bottom panel) and lateral size (top panel) of the AMO flakes. **e** X-ray photoelectron spectroscopy (XPS) of the single crystalline AMO. **f** X-ray diffraction (XRD) analysis of the crystalline AMO. **g** Diagram of formation energy vs energy above hull (*E*_hull_) for the metal molybdates, a lower *E*_hull_ indicates higher thermodynamic stability. All data were retrieved from the Materials Project database^52^. Scale bars in (**b**) and (**c**) are 10 μm.

Single-crystalline AMO flakes were synthesized using a confined-space strategy. A molybdenum foil was clamped between two sapphire wafers to form a sandwich structure (Fig. 1a) and heated at 700 °C in air. During heating, the Mo foil oxidized and sublimated, providing a continuous vapor source that reacted with the sapphire to form laterally oriented flakes (Supplementary Fig. 1).

The as-grown flakes exhibit a well-defined crystallographic orientation, frequently intersecting at angles that are multiples of 30° (Fig. 1b). Large-area, square-shaped crystals with diverse thicknesses and lateral sizes were synthesized on a 2-inch c-plane sapphire substrate, achieving near-complete surface coverage (Supplementary Fig. 2). We employed atomic force microscopy (AFM) to determine the thickness and lateral dimensions of the flakes. A representative AFM image reveals a square AMO crystal with a height of ~155 nm (Fig. 1c). AFM roughness analysis yields a root-mean-square (RMS) roughness of 21 pm, indicating the smooth surface of AMO flakes (Supplementary Fig. 3). Statistical analysis of numerous flakes (Fig. 1d) shows that the thicknesses range from 50 to 500 nm and lateral sizes from 1 to 50 µm, both with narrow distributions, demonstrating good synthetic controllability.

X-ray photoelectron spectroscopy (XPS) survey scans detected no elements other than Al, Mo, and O (Supplementary Fig. 4). The high-resolution O 1 *s* and Al 2*p* peaks are centered at 531.23 eV and 74.33 eV, respectively, with no additional chemical states (Fig. 1e). Deconvolution of the Mo 3 *d* spectrum indicates the coexistence of Mo⁶⁺ and a small amount of Mo⁵⁺, with a relative ratio of ~13.29. Based on the electroneutrality principle, this ratio suggests nearly ideal oxygen stoichiometry and a negligible concentration of oxygen vacancies.

The X-ray diffraction (XRD) pattern in Fig. 1f represents an area-averaged signal from the sample, including both the sapphire substrate and the sparse AMO coverage. A series of sharp peaks at 2*θ* = 14.14°, 28.38°, 43.13°, 58.66°, 75.48°, can be indexed as the (002), (004), (006), (008), and (0010) reflections of AMO flakes. The presence of only (00 *l*) peaks indicates that the (00 *l*) planes are preferentially oriented parallel to the substrate surface. Using Bragg’s law (2 *d* sin*θ* = n*λ*, *λ* = 1.54 Å for Cu *K*_α_), the interplanar spacing for the (008) reflection is calculated to be 12.57 Å, which corresponds to *c*·sin(*β*) = 12.64 Å in the monoclinic AMO (space group *P2*_*1*_*/a*, lattice parameters *a* = 9.0 Å, *b* = 9.1 Å, *c* = 15.49 Å, *β* = 125.3°), consistent with previous reports^8^. On the basis of the above findings, we can preliminarily determine that these flakes are single-crystalline AMO.

### Atomic structural characterizations of AMO crystals

**Fig. 2:**
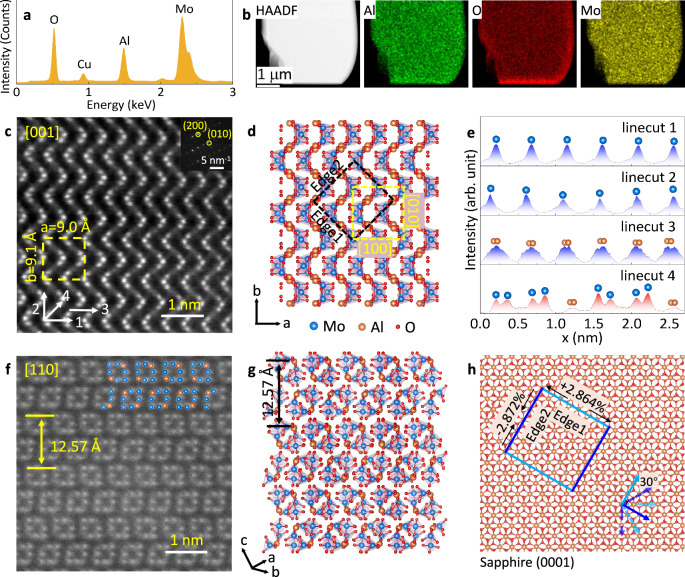
Atomic structure characterization of the AMO crystal. **a** The characteristic EDX spectrum of an AMO flake. **b** Energy-dispersive X-ray (EDX) spectroscopy mapping of aluminum (green), oxygen (red), and molybdenum (yellow) elements on the AMO flake. **c** High-angle annular dark-field scanning transmission electron microscopy (HAADF-STEM) image of AMO flake viewed along the [001] axis. Inset is the corresponding selected area electron diffraction (SAED) of a square AMO flake along [001] zone axis. **d** Atomic structure projection of AMO crystal onto the (001) plane, blue, orange, and red spheres represent Mo, Al, and O atoms, respectively. Edge1 and edge2 are used to denote the two orthogonal edges of the square-like AMO flake. The crystallographic axes [100] and [010] are denoted as a- and b-axes, respectively. **e** Line profiles of angular dark-field STEM image taken along line 1 & 2 (top panel) and line 3 & 4 (bottom panel), these lines are marked in (**c**) with white solid arrows. **f** Cross-sectional HAADF-STEM image of an AMO flake taken along the edge. **g** Atomic structure projection of AMO crystal onto the (110) plane. **h** Epitaxial relationships between AMO and sapphire (0001).

The crystal structure of the AMO flakes was elucidated through high-resolution transmission electron microscopy (HRTEM). Energy-dispersive X-ray spectroscopy (EDX) confirms the chemical composition, showing only Al, Mo, and O signals (aside from the Cu grid background) with an atomic ratio close to 2:3:12 (Fig. 2a). Corresponding elemental maps demonstrate uniform spatial distribution of all three elements, indicating high chemical homogeneity (Fig. 2b). The edge brightening observed in the oxygen map is attributed to sample charging. This was confirmed by transferring an AMO flake onto a conductive gold substrate, where EDX mapping (Supplementary Fig. 5) revealed uniform elemental distribution, demonstrating compositional homogeneity.

High-angle annular dark-field scanning transmission electron microscopy (HAADF-STEM) further reveals a periodic zig-zag structure in the basal plane (Fig. 2c). Given that image intensity scales with atomic number (Z) as ~Z^1.7^^24^, the brightest columns correspond to Mo atoms (Z = 42) at the zigzag vertices, while the weaker columns represent Al dimers (Z = 13) bridging adjacent vertices. The selected-area electron diffraction (SAED) pattern (Fig. 2c inset) displays a single set of sharp spots, confirming the flakes’ high single-crystalline quality. These observations align well with the expected monoclinic AMO structure (Fig. 2d).

Line profiles extracted along specific crystallographic directions provide quantitative lattice information (Fig. 2e). Linecut 1, taken along Mo atomic rows, shows a 4.5 Å periodicity. Linecut 2, crossing alternating chiral vertices, exhibits a 4.55 Å spacing—half the unit-cell length. Linecut 3, traced along Al dimer rows, shows consistent periodicity, while linecut 4, traversing both Mo and Al columns, clearly differentiates their intensity and width.

Cross-sectional HRTEM was performed only on a specimen prepared along edge 1. The results (Fig. 2f) visualize the alternating arrangement of AlO₆ octahedra and MoO₄ tetrahedra, yielding a spatial periodicity of 12.57 Å along the direction perpendicular to the sample surface, which is consistent with the XRD measurement. The cross-sectional HRTEM image perfectly matches the atomic structure projection of AMO onto the (110) plane. This allows us to conclude that the two orthogonal edges of the AMO nanoflake are parallel to the (110) plane and intersect the [100] or [010] axes at an angle of 45°. Consequently, combined analysis identifies the flake edges parallel to Mo–Al–Al–Mo atomic chains with a periodicity of 2.543 nm, and are crystallographically equivalent.

The epitaxial relationship with the sapphire (0001) substrate is quantified in Fig. 2h. The lattice mismatch is +2.86% along the AMO edge1 direction (sapphire Al–O–O–Al period: 0.476 nm) and –2.87% along the AMO edge2 direction (sapphire Al–Al period: 0.412 nm), indicating tensile strain along edge1 and compressive strain along edge2 direction. This biaxial strain state, consistent with the threefold symmetry of the sapphire surface, explains the observed 30° multiples in flake orientations (Fig. 1b). The epitaxial relationship between AMO and sapphire is thus unambiguously established.

To elucidate the growth mechanism, we presented the formation energy and energy above hull for relevant compounds in Fig. 1g. The AMO crystal exhibits the lowest formation energy (–2.401 eV) and an energy above hull of 0 eV, confirming its superior thermodynamic stability and explaining its preferential nucleation over other molybdenum oxides.

Building on this thermodynamic preference and the known propensity for transition metal aluminate formation at oxide interfaces^25^, we propose a kinetic model for AMO nucleation and growth (Fig. 1a, right panel). Initially, interdiffusion at the MoO₃/Al₂O₃ interface enables the rapid incorporation of Al³⁺ ions into adsorbed MoO₃, leading to the local formation of few-layer AMO nanosheets. Subsequently, thermally driven migration of Al³⁺ ions along crystal boundaries facilitates continuous chemical bonding with Mo⁶⁺ ions, ensuring a consistent vertical stacking sequence. The overall reaction is governed by the following equations:

$$2{{{\rm{Mo}}}}+3{{{{\rm{O}}}}}_{2}\to 2{{{{\rm{MoO}}}}}_{3}$$

$${{{{\rm{Al}}}}}_{2}{{{{\rm{O}}}}}_{3}+3{{{{\rm{MoO}}}}}_{3}\to {{{{\rm{Al}}}}}_{2}{({{{{\rm{MoO}}}}}_{4})}_{3}$$

The resulting monoclinic AMO structure, composed of corner-sharing AlO₆ octahedra and MoO₄ tetrahedra, is schematically illustrated in Fig. 1a. This interfacial epitaxy strategy, operating under moderate conditions, enables efficient precursor utilization and yields high-quality crystals, thereby establishing a general pathway for synthesizing ternary metal molybdates.

### Phonon modes of AMO crystals via Raman spectroscopy

**Fig. 3:**
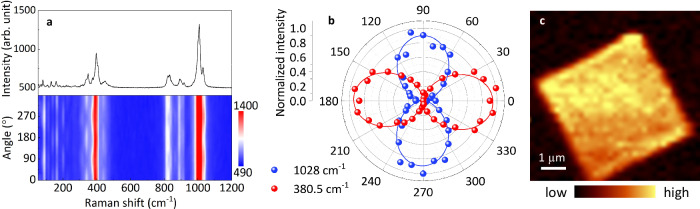
Raman spectroscopy of the AMO crystal. **a** Top panel: Raman spectrum of the as-grown AMO flake. Bottom panel: color map of the polarization dependence of each Raman scattering mode. **b** Intensity of the *A*_g_ modes at 380.5 and 1028 cm^−1^ plotted in polar coordinates. The red and blue solid spheres are the measured data points of these two modes at each polarization angle, and the solid lines are the corresponding fits to the polarimetric response. **c** Raman intensity mapping with the peak centered at 1001.8 cm^−1^.

The phonon properties of biaxially strained AMO flakes were investigated using Raman spectroscopy with a 532 nm excitation laser (30–1200 cm⁻¹ range). Consistent with prior studies on related polycrystalline molybdates and tungstates^7,9,26^, the observed Raman peaks are attributed to vibrational modes of the (MoO₄)²⁻ tetrahedra and their coupling to Al³⁺ neighbors. Specifically, the strong bands between 930 and 1050 cm⁻¹ originate from symmetric Mo–O stretching vibrations, while features between 270 and 400 cm⁻¹ correspond to O–Mo–O bending modes (Fig. 3a).

Polarization-dependent Raman measurements reveal a pronounced anisotropy in the AMO flakes. The intensity of the *A*_g_ modes at 380.5 and 1028 cm⁻¹ exhibits pronounced angular dependence characteristic of the biaxially strained AMO flake. These anisotropic optical phonon modes are expected to govern the macroscopic anisotropy in thermal conductivity and mechanical elasticity.

Raman mapping at 1001.8 cm⁻¹ on a 4.5 × 4.5 μm² AMO square flake further confirms the high spatial homogeneity of phonon vibrations (Fig. 3c). Moreover, the spectral range from 800 to 1100 cm⁻¹ shows coincident responses in both PiFM and Raman scattering spectra (Supplementary Fig. 6), indicating strong light–phonon interaction. Within this Reststrahlen band, photon absorption efficiently excites optical phonons, leading to the formation of hybrid quasiparticles—phonon polaritons (PhPs).

### Unraveling PhPs propagation in AMO crystals via PiFM

**Fig. 4:**
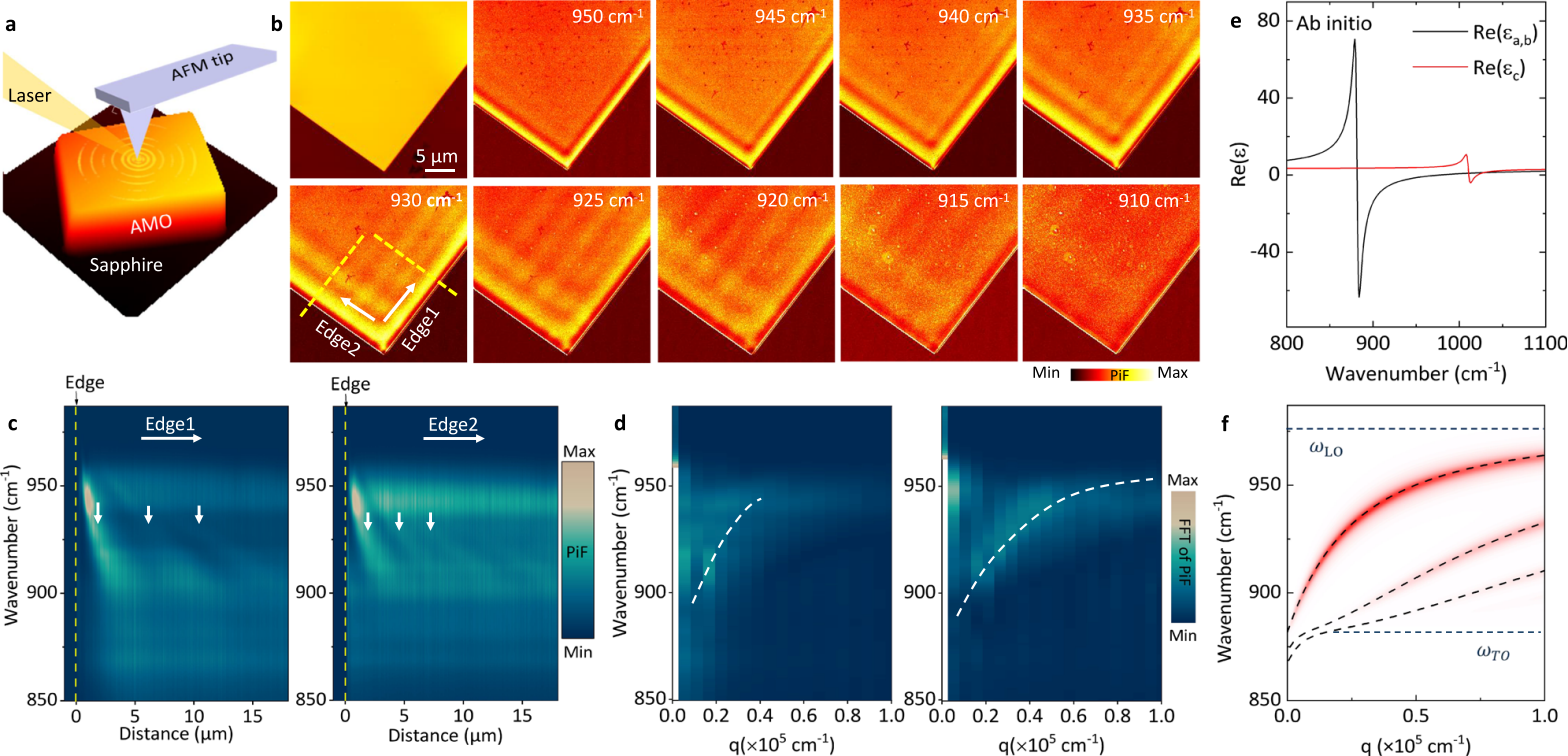
Anisotropic phonon-photon coupling in AMO flakes. **a** Schematic of the photo-induced force microscopy (PiFM) setup that includes an AFM tip and an incident laser. **b** AFM image of a 470-nm-thick AMO square, scale bar is 5 µm. A series of PiFM images is acquired with incident frequencies of 950 to 910 cm^−1^, respectively. **c** Line scans of PiF spectra along edge1 and edge2 orientations of AMO crystal as marked by the dashed lines in (**b**). Solid arrows are used to indicate the distance-polaritonic peaks dependence relative to the AMO edge. The vertical dashed lines indicate the positions of the sample edges. **d** The fast Fourier transform (FFT) of (**c**) revealing dispersions in momentum space. The dashed lines are guides to the eye, highlighting regions with high spectral weight. **e** Calculated real parts of the permittivity along [110] and [001] direction in AMO crystal from 800 cm^−1^ to 1100 cm^−1^. **f** Calculated phonon polaritons (PhPs) dispersion (wavenmber vs. in-plane wavevector) with the analytical solution (dashed curves), *ω*_TO_ = 881.5 cm^−1^ and *ω*_LO_ = 976.2 cm^−1^ are marked as transverse optical (TO) and longitudinal optical (LO) phonon modes, respectively.

We employed photo-induced force microscopy (PiFM) to investigate the PhPs in AMO flakes with nanoscale resolution. The PiFM setup (Fig. 4a) operates by focusing incident infrared light onto a Pt/Ir-coated atomic force microscope (AFM) tip, enhancing its local field. The subsequent dipole-dipole interaction between the tip and the sample generates a photo-induced force that encodes the local polaritonic field distribution, allowing simultaneous mapping of topography and near-field optical response^27,28^.

Spatial imaging of a large AMO flake from 910 to 950 cm⁻¹ revealed edge-launched PhPs, which are generated by scattering of the incident field at the sample edges (Fig. 4b), where the interference pattern varies dramatically with excitation frequency. Crucially, these edge-launched PhPs exhibit distinct periodicities along the two principal crystal axes: line profiles (Supplementary Fig. 7a) yield a polariton period of λ₁ = 6.5 μm along the edge1 direction and λ₂ = 4.3 μm along the edge2 direction. The polariton wavelength along edge1 (λ₁) is approximately 1.33 times larger than that along edge2 (λ₂), providing direct evidence of in-plane anisotropy. As summarized in Supplementary Table 1, the quality factor (*Q*)^29^ along the two orthogonal edges are respectively 4.48 (edge1) and 4.86 (edge2) via normalizing the propagation length (*L*_p_) in Supplementary Fig. 7b by the wavelength (λ), both falling within the lower range of values reported for quaternary oxides^12^ and Li/Na-V_2_O_5_^30,31^. Given that edge1 and edge2 are crystallographically equivalent (both oriented at 45° to the [100] and [010] axes), this anisotropy cannot be attributed to the intrinsic lattice asymmetry. Instead, it points to an external perturbation, most likely the biaxial epitaxial strain—tensile along edge1 and compressive along edge2—imposed by the sapphire substrate during the interfacial epitaxy process, which can be evidenced by the crystal-orientation determined polariton anisotropy (Supplementary Fig. 8). In addition, we further observed a tunable polariton response by examining crystals with different thicknesses: the polariton wavelength scales with the flake thickness in Supplementary Fig. 9, consistent with the thickness-dependent dispersion expected for strongly confined polaritons^32^. We note that the potential role of strain in modulating the edge-launched polariton modes observed here remains qualitative. A systematic, quantitative analysis of strain effects would require controlled strain experiments combined and systematic numerical simulations, which we identify as important directions for future investigation.

To quantify this anisotropy, we acquired line-scan PiFM spectra along the edge1 and edge2 directions over the spectral range of 850–990 cm⁻¹ (Fig. 4c). The resulting spectra exhibit a series of distance- and energy-dependent fringe maxima, which arise from interference between the polariton field launched by the tip (subsequently reflected from the edge) and the background field at the tip. Applying fast Fourier transform (FFT) to these line scans yields well-resolved, distinct dispersion branches in moment space for the two orthogonal directions (Fig. 4d). Notably, as the excitation frequency increases from 900 to 970 cm⁻¹, the polariton wavelength systematically decreases, a characteristic hallmark of type-II hyperbolic dispersion^33,34^. To identify our hypothesis, we have calculated the permittivity tensor using DFT (Fig. 4e and Supplementary Table 2). The resulting ab initio permittivity function qualitatively agrees with the experimental PiFM and Raman scattering spectra (Supplementary Fig. 10b). There is obvious discrepancy between the [110] and [001] directions in the range of 800–1100 cm^−1^, the isofrequency surfaces in Supplementary Fig. 10c, defined by *ω*(**k**_⊥_, **k**_∥_) = *ω*_0_ for a given frequency 930 cm^−1^, indicates the hyperbolic nature of AMO crystal, i.e., in-plane elliptical phonon polaritons and out-of-plane hyperbolic phonon polaritons. The calculated dispersion curves of AMO crystal with the TO and LO frequencies clearly labeled, are presented in Fig. 4f by numerical simulations, which shows good agreement with the analytical solution (dashed curves), thereby validating the experimental wavevector–frequency relationship.

### PhPs confinement in AMO crystals

**Fig. 5:**
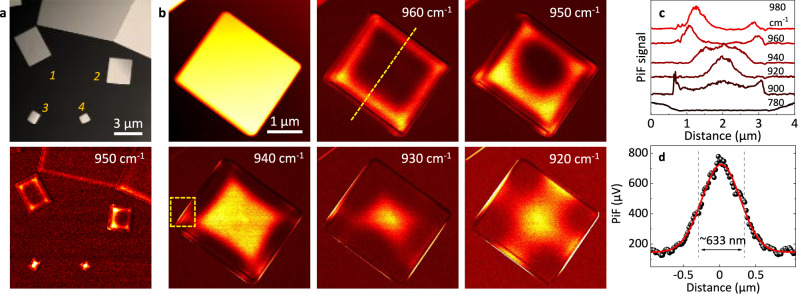
PhPs confinement in AMO crystal. **a** AFM and PiFM images of the as-grown AMO flakes on a sapphire substrate with various lateral sizes. The thickness (*d*) of isolated crystals as marked with #1 ~ #4 are 277, 283, 328, and 325 nm, respectively. **b** AFM and PiFM images of a small isolated AMO crystal with incident frequencies from 960 to 920 cm^−1^, dashed rectangle is used to highlight the area with interference fringes near the crystal edge. **c** PiFM line profiles along the yellow dashed line taken at different incident frequencies. **d** Detailed PiFM line profile of the hotspot at (**b**) 930 cm^−1^ exhibiting the focusing performance of AMO.

We further explored polariton confinement by performing PiFM on AMO flakes with controlled lateral dimensions (*l*₁ = 2.5, *l*₂ = 2.25, *l*₃ = 0.84, *l*₄ = 0.64 μm; Fig. 5a). At a fixed frequency of 950 cm⁻¹, the near-field distribution transitions from a dark central region encircled by a bright ring in larger flakes (#1, #2) to a single central bright spot in smaller flakes (#3, #4). This size-dependent mode evolution confirms that the lateral dimension acts as a critical cavity boundary for confined PhPs.

A detailed frequency scan from 920 to 960 cm⁻¹ on a 270-nm-thick, 2.56×2.26 μm² flake (Fig. 5b) reveals a rich evolution of cavity modes as the excitation wavenumber decreases. Initially, the edges of the square flake light up at 960 cm^−1^ due to efficient polariton launching. Subsequently, a bright ring forms and progressively shrinks at 950 cm^−1^, eventually converging into a single, intense hot spot at the center of the flake (940 and 930 cm^−1^). The subsequent emergence of new modes with asymmetric behavior on adjacent sides (920 cm⁻¹) further indicates strong in‑plane anisotropy. The fringes highlighted by yellow dashed rectangles, which exhibit a distinctly smaller periodicity than the fundamental mode, are most likely attributable to higher-order (*l* = 1) polariton modes^35^. To quantitatively link these short-wavelength fringes to the hyperbolic dispersion, we recalculated the polariton dispersion for the actual thickness of the AMO nanoplate in Fig. 5 (270 nm, different from the 470 nm flake in Fig. 4). Spatial Fourier analysis^35^ of the fringes at 930 cm⁻¹ and 940 cm⁻¹ yielded in-plane momenta that fall precisely on the calculated l=1 branch (Supplementary Fig. 11c). Although strong lateral confinement prevented observation of the l=0 mode in this small nanoplate, the theoretical separation $\Delta q={q}_{l=1}-{q}_{l=0}=\pi \psi /d$ gave a hyperbolic figure of merit ψ consistent with the value derived directly from the permittivity tensor (Supplementary Fig. 11d). This confirms the hyperbolic “beaming” character of the polaritons in AMO. Notably, no distinct features are observed when the incident frequency lies outside the Reststrahlen bands.

Line profiles (Fig. 5c, d) quantify this evolution, showing a systematic reduction in fringe spacing as the frequency decreases. The central hotspot at 930 cm⁻¹ has a full width at half maximum (FWHM) of approximately 633 nm, yielding a confinement factor (λ_IR_/λ_p_) of about 17. This strong confinement underscores AMO’s potential for sub-diffractional photonic devices such as superlenses or hyperlenses.

This confinement could be further optimized by tuning the lateral dimensions, thickness, and the incident frequency. To further examine the influence of microstructure geometry on the PiFM signals, we conducted analogous measurements on a rectangular AMO flake (3.5 × 2.67 μm², Supplementary Fig. 12), which exhibited a comparable frequency-dependent evolution but with different near-field interference patterns. The marked variations in these standing wave patterns confirm the presence of Fabry–Pérot resonances^34^, which can be systematically engineered by adjusting the size of the AMO flakes. Therefore, tailoring AMO flake dimensions, as well as via biaxial strain epitaxy, offers a viable approach to modulate PhP interference and achieve configurable spatial distributions.

## Discussion

Moreover, the polaritonic modes in AMO flakes emerge within a spectral window of 900–970 cm⁻¹, which overlaps with the Reststrahlen band of α-MoO₃ while extending to slightly lower wavenumbers. This spectral region therefore complements the existing mid-infrared polaritonic landscape (e.g., *h*-BN^32,36^ and *α*-MoO_3_^37–40^) by offering an additional material platform (Table 1). In this context, the successful synthesis of high-quality, single-crystalline AMO—characterized by its distinct crystal symmetry, facile synthetic route, and robust stability—further expands the materials toolkit for nanophotonic and optoelectronic applications. To achieve large‑area single‑crystalline AMO, plasma‑induced surface defects on sapphire could be used to regulate the nucleation density and lateral expansion of AMO domains, while transfer to arbitrary substrates could become feasible by using water‑soluble Sr_4_Al_2_O_7_ (SAO) as a sacrificial growth substrate. These strategies may pave the way for scalable, integrable AMO membranes in future investigations.

**Table 1:** Comparison of PhP properties among AMO, *h*-BN, and α‑MoO₃

Parameters	Al_2_(MoO_4_)_3_	*h*-BN	*α*-MoO_3_
**Polariton Type**	Hyperbolic	Hyperbolic	In-plane Hyperbolic; Elliptical
**Anisotropy**	In-plane anisotropy	Out-of-plane anisotropy	Out /In-plane anisotropy
**Operational Frequency Range**	Type II: 900-970 cm^−1^	Type II: ~1370 - 1610 cm⁻¹ Type I: ~780 - 830 cm⁻^1^	band1: ~545–553 cm⁻¹ band2: ~820–973 cm⁻¹ band3: ~1000–1020 cm⁻^1^
**Spectral Tunability**	Epitaxial strain/Lateral sizes	Thickness^44^/Hybrid polaritons with substrates^45^ /Interfacial near-field coupling^46,47^	Thickness^37^/ Hybrid polaritons with the environment^27^/Rotating crystal^38^.
**Polariton Quality Factor**	4.86	50–480^48^	200^49^ 40^50^ 76^51^
**Synthesis Methods**	Interfacial epitaxy	Mechanical exfoliation / CVD	Mechanical exfoliation / CVD

In conclusion, we have developed a general interfacial epitaxy strategy and demonstrated its efficacy by synthesizing high-quality, single-crystalline flakes of AMO. The as-grown flakes possess a well-defined biaxial strain, which leads to pronounced in-plane anisotropy in the propagation of hyperbolic phonon polaritons (PhPs). We have further established that the polaritonic interference patterns can be effectively tailored by the excitation frequency and the lateral dimensions of the flake. Beyond establishing a general synthesis platform for a coveted class of materials, this work introduces a material of interest for infrared polaritonics. The capability to engineer polaritonic behavior in AMO opens immediate opportunities for designing nanophotonic devices and positions strain-engineered ternary oxides as a frontier for exploring low-loss and tailorable light confinement across the mid-infrared to terahertz spectrum.

## Methods

### Sample preparation

The Al_2_(MoO_4_)_3_ nanoflakes were synthesized by a space-confined method with a Mo foil (thickness: 0.05 mm, lateral dimensions: 5 mm × 5 mm, purity: 99.99%) sandwiched between two sapphire substrates. The sandwich structure was then heated in air to 700 °C at 5 °C/min, maintained for several hours, and then furnace-cooled to room temperature, where the oxidized Mo foil acted as the MoO_3_ precursors and reacted with the Al_2_O_3_ substrate, driving the interfacial nucleation of Al_2_(MoO_4_)_3_ nanoflakes.

We attempted to transfer AMO flakes onto alternative substrates (CaF₂, Au-coated Si) using dry-transfer methods. However, due to the strong chemical bonding at the Al_2_(MoO_4_)_3_/sapphire interface resulting from the in-situ growth reaction, the flakes proved resistant to exfoliation and frequently fractured upon attempted transfer. Only small fragments (<5 µm) could be obtained, precluding polariton imaging. This strong adhesion contrasts with the weak van der Waals interactions in typical exfoliable 2D materials and underscores the unique growth mechanism of AMO.

#### Characterizations of the crystal and topographic structures

##### X-ray diffraction

X-ray diffraction (XRD) patterns were obtained using a SmartLab SE diffractometer (Rigaku, Japan) with Cu *K*_*α*_ radiation (λ = 1.5406 Å), data were collected in the 2*θ* range from 10° to 80°.

##### STEM measurements

The TEM samples were prepared using focused ion beam (FIB) (ThermoFisher Helios 5UX). Lamellae were prepared along the edge of the Al_2_(MoO_4_)_3_ nanoflakes. High-resolution HAADF-STEM images were acquired using a ThermoFisher Spectra 300.

##### AFM measurements

The topography of the Al_2_(MoO_4_)_3_ nanoflakes were imaged in contact-mode by a Cypher S AFM with commercially available PtIr-coated Si tip. The root-mean-square (RMS) roughness was calculated using standard AFM analysis software (AR 16.10.211).

### PiFM measurements

Near-field optical images were acquired using a commercial atomic force microscope (VistaScope, Molecular Vista, USA) equipped with a photo-induced force microscopy (PiFM) module. All PiFM measurements were performed using a pulsed, widely tunable quantum cascade laser (Block Engineering, USA) as the excitation source, covering a seamless narrowband wavenumber range of 780–1950 cm^−1^. The laser features a spectral linewidth of 2 cm^−1^ and a resolution of 0.5 cm^−1^.

The experiments were operated in sideband excitation mode, where the laser frequency was modulated at *f*_m_ = *f*_1_ − *f*_0_. Here, *f*_0_ represents the first mechanical eigenmode resonance of the cantilever used for PiF signal detection, while *f*_1_ corresponds to the second eigenmode resonance employed for the simultaneous acquisition of AFM topography. The spectra were acquired by recording the PiF signal while tuning the laser wavenumber. To account for the laser power variation across the tuning range, all spectra were normalized by the laser power profile, which was measured simultaneously during the scan. As to the observed fringe period, we note that the correction^41^ is modest (within ±5–10%) and does not affect the key conclusions in our study.

Gold-coated silicon probes (HQ:NSC15/CR-AU, MikroMasch Inc.) with a resonant frequency of approximately 300 kHz were used for all measurements.

#### Theoretical calculation

##### Formation energy above hull (*E*_hull_)

In essence, the energy above hull is a descriptor of thermodynamic stability derived from first-principles calculations. It represents the energy difference between a given structure and the most stable combination of competing phases at the same chemical composition (the convex hull). A value of *E*_hull_ = 0 meV/atom indicates a stable (ground state) phase that is thermodynamically stable against decomposition. Conversely, a positive value indicates a metastable phase; the smaller the value, the more likely the phase is to be experimentally synthesizable. For example, a material with *E*_hull_ < 70 meV/atom is often considered potentially synthesizable^42^. The thermodynamic stability data presented in Fig. 1g were obtained from the Materials Project database (https://materialsproject.org). The specific material identifiers used are: [AMO: mp-19521], [MoO_2_: mp-559140], [MoO_3_: mp-20589], [MoO_4_: mp-1180203], [Mo_4_O_11_: mp-542610], and [Al_2_O_3_: mp-1143].

##### Permittivity of AMO

The frequency-dependent dielectric function of AMO crystal was computed using density functional perturbation theory (DFPT) as implemented in VASP with the PBE functional. The perpendicular ${\varepsilon }_{{{\rm{c}}}}$ and parallel ${\varepsilon }_{{{\rm{a}}},{{\rm{b}}}}$ components of the permittivity tensor are approximated with a Drude-Lorentz model^33^.

$${\epsilon }_{a}\left(\omega \right)={\epsilon }_{a,\infty }\left(1+\frac{{({\omega }_{{LO}}^{a})}^{2}-{({\omega }_{{TO}}^{a})}^{2}}{{({\omega }_{{TO}}^{a})}^{2}-{\omega }^{2}-i\omega {\gamma }^{a}}\right)$$

${\omega }_{{TO}}$ and ${\omega }_{{LO}}$ refer to the transversal (TO) and longitudinal (LO) phonon frequencies, The high-frequency dielectric tensor ${\varepsilon }_{\infty }$ was taken from the independent-particle calculation, and a damping constant *γ* = 5 cm^−1^ was used for all oscillators. The in-plane (${\varepsilon }_{{{\rm{a}}},{{\rm{b}}}}$) and out-of-plane (${\varepsilon }_{{{\rm{c}}}}$) components were evaluated separately to reflect the anisotropic response of the monoclinic crystal.

##### Theoretical Calculation of the Dispersion Diagrams

The phonon polariton (PhP) dispersion of AMO was calculated using a multilayer model consisting of an AMO flake on a semi-infinite sapphire substrate, with air as the superstrate, the corresponding interface reflectivity are ${r}_{a}$ and ${r}_{s}$. The complex reflectivity ${r}_{p}(q,\omega )$ contains the information of phonon polariton^43^, reflectivity of the interface from air to the AMO layer ${r}_{p}$ can be expressed as

$${r}_{a}=\frac{{\varepsilon }^{\perp }{k}_{a}^{z}-{\varepsilon }_{a}{k}_{e}^{z}}{{\varepsilon }^{\perp }{k}_{a}^{z}+{\varepsilon }_{a}{k}_{e}^{z}}$$

$${r}_{s}=\frac{{\varepsilon }_{s}{k}_{e}^{z}-{\varepsilon }^{\perp }{k}_{s}^{z}}{{\varepsilon }_{s}{k}_{e}^{z}+{\varepsilon }^{\perp }{\varepsilon }_{s}^{z}}$$

$${r}_{p}=\frac{{r}_{a}+{r}_{s}{e}^{i2{k}_{e}^{z}d}}{1+{r}_{a}{r}_{s}{e}^{i2{k}_{e}^{z}d}}$$

Where ${\varepsilon }_{a},{\varepsilon }^{\perp }$, *and* ${\varepsilon }_{s}$ refer to the permittivity of air, AMO (out of plane component), and substrate, respectively. ${k}_{a}^{z}$ or ${k}_{s}^{z}$ represents the *z*-axis component momentum of the photon in air or substrate, the momentum ${k}_{e}^{z}$ corresponding to an extraordinary ray of AMO due to the anisotropic properties. The *d* represents the thickness of AMO crystal. For ${\lambda }_{p}\ll {\lambda }_{{IR}}$, we derived the analytical formula for polariton dispersion:

$$q\left(\omega \right)+i\kappa \left(\omega \right)=-\frac{\psi }{d}\left[\arctan \left(\frac{{\varepsilon }_{a}}{{\varepsilon }_{\perp }\psi }\right)+\arctan \left(\frac{{\varepsilon }_{s}}{{\varepsilon }_{\perp }\psi }\right)+\pi l\right],\psi=\frac{\sqrt{{\varepsilon }_{\parallel }}}{i\sqrt{{\varepsilon }_{\perp }}}$$

Where ${\varepsilon }_{a}(\omega )$, ${\varepsilon }_{\perp }(\omega )$, ${\varepsilon }_{\parallel }(\omega )$, and ${\varepsilon }_{s}(\omega )$ are the dieletric function for air, AMO (for out-of-plane direction), AMO (for in-plane direction), and substrate.

## Supplementary information

Supplementary Information

Transparent Peer Review file

## Data Availability

Relevant data supporting the key findings of this study are available within the article and the Supplementary Information file. All raw data generated during the current study are available from the corresponding authors upon request.
