# Supplementary material for: Interfacial epitaxy of single-crystalline Al2(MoO4)3 flakes for anisotropic phonon polaritons
Source: Nat Commun. 2026 Jun 25;17:7956. doi: 10.1038/s41467-026-74681-x (PMC13448070; doi:10.1038/s41467-026-74681-x)
Supplement: Supplementary file 1 [file 41467_2026_74681_MOESM1_ESM.pdf]

Supplementary Information for
Interfacial Epitaxy of Single-crystalline $Al_2(MoO_4)_3$ Flakes for Anisotropic Phonon Polaritons

Chuangye Song,[1,2#*] Jiawei Huang,[3#] Song Zhou,[1] Yuanhao Lyu,[1] Xiaoyue He,[3] Shaoxiang Sheng,[1*] Kehui Wu[1*]

[1]Tsientang Institute for Advanced Study, Hangzhou 310024, China

[2]School of Mathematics and Physics, North China Electric Power University, Beijing 102206, China

[3]Songshan Lake Materials Laboratory, Dongguan 523808, China

Corresponding author: khwu@tias.ac.cn; sxsheng@tias.ac.cn; cysong@tias.ac.cn;

[#]These authors contributed equally to this work.

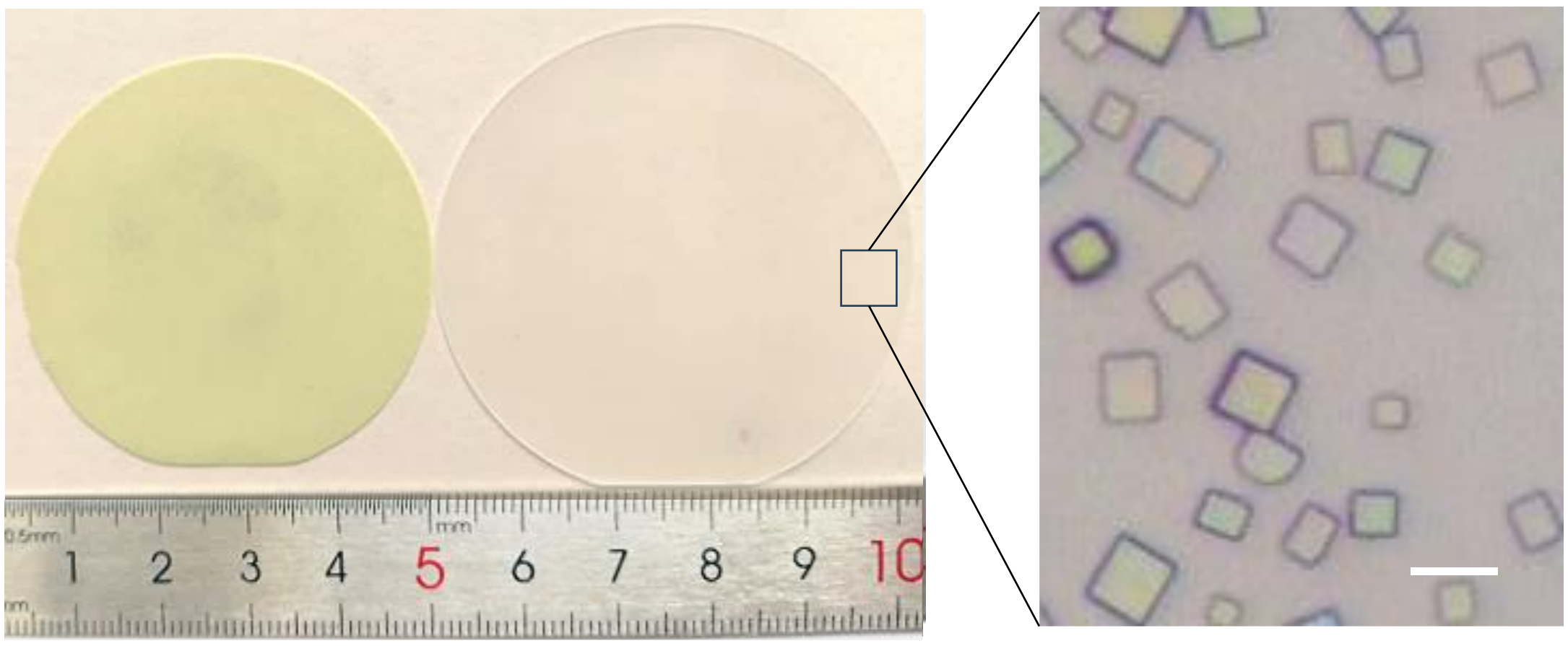

Supplementary Figure 1. Optical photograph of the oxidized Mo foil (left) and sapphire wafer (right) after the growth. The square AMO flakes can cover the 2-inch wafer and even larger area depending on the size of the Mo foil, the scale bar is 10 μm.

a b

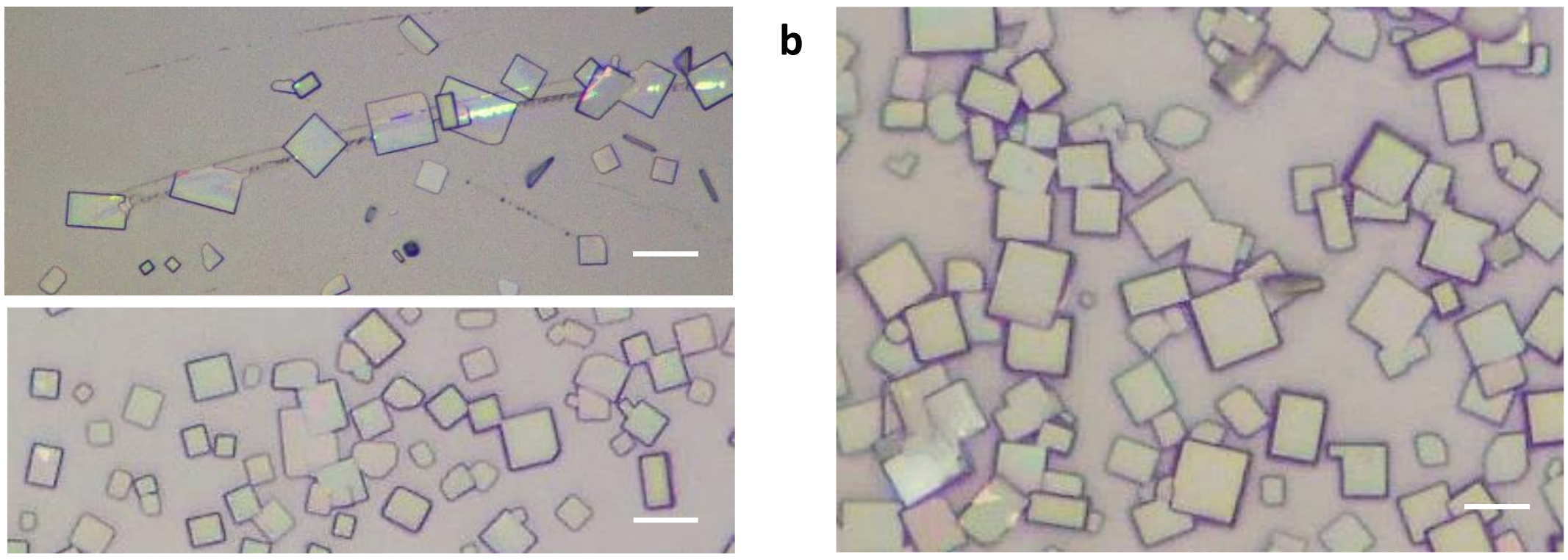

Supplementary Figure 2. (a - b) Optical microscopy images of the as-grown AMO crystals on sapphire substrates, the scale bars are 10 μm.

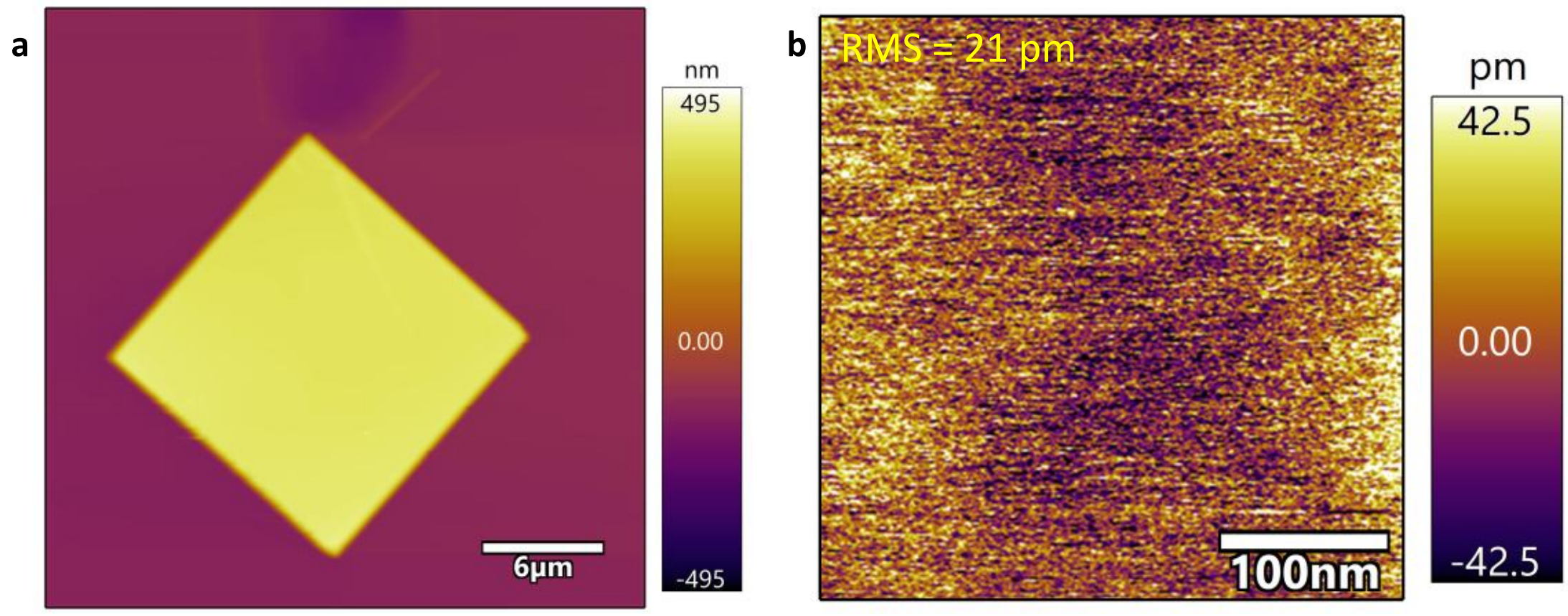

Supplementary Figure 3. (a) AFM image of an AMO flake. (b) Zoom-in image image of the AMO flake where the RMS is calculated. The root-mean-square (RMS) roughness was calculated using standard AFM analysis software (AR 16.10.211). We selected multiple regions of 0.35 μm × 0.35 μm, the RMS roughness measured in all these regions is 21 pm (0.021 nm). This value is representative of those obtained across multiple flakes and regions.

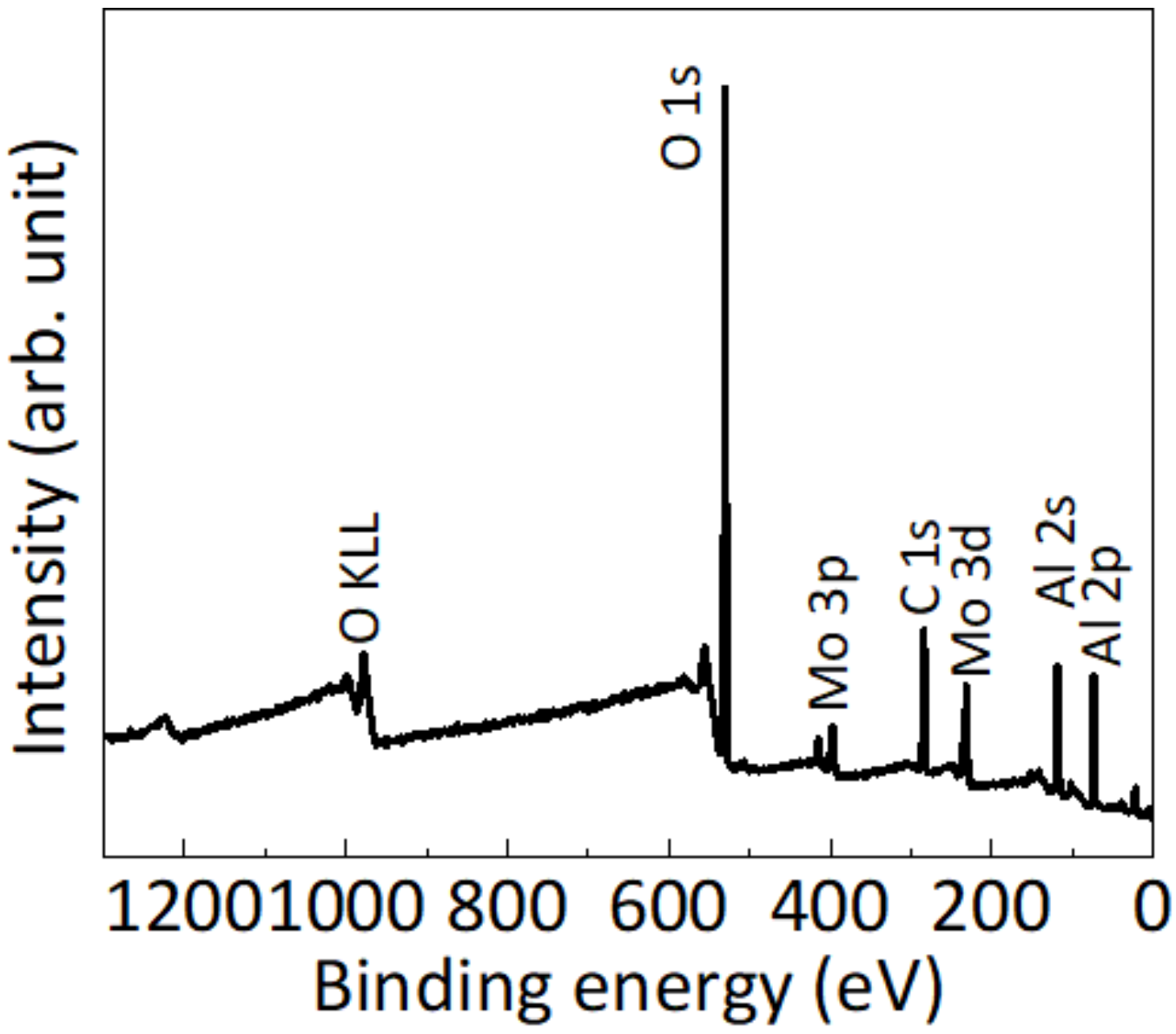

Supplementary Figure 4. XPS spectra of the AMO crystals on sapphire substrate.

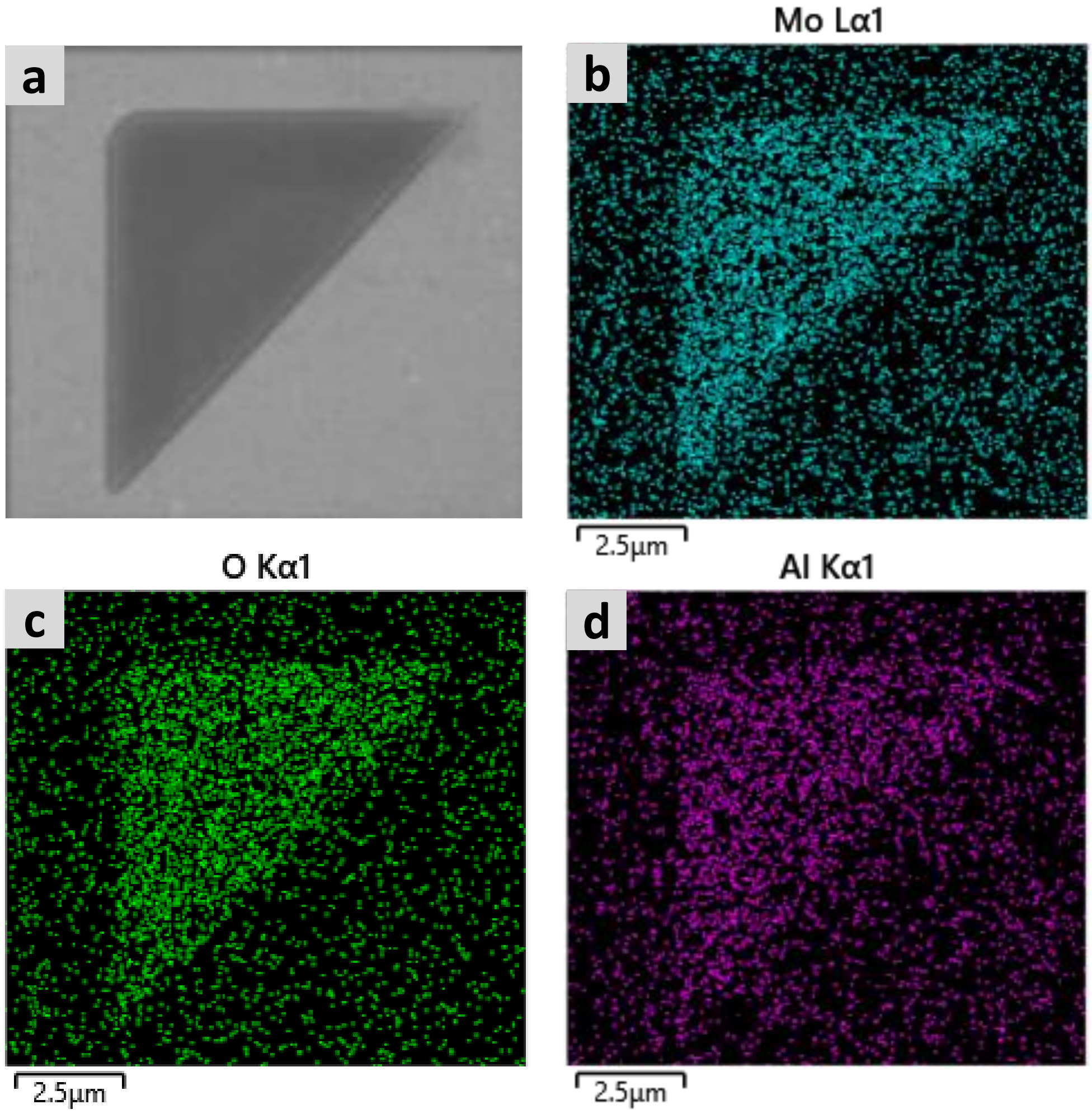

Supplementary Figure 5. (a) SEM image of a transferred AMO flake. EDX mapping of molybdenum (b), oxygen (c) and aluminum (d) elements was performed after transferring the AMO flake onto a conductive gold thin film to eliminate charging artifacts.

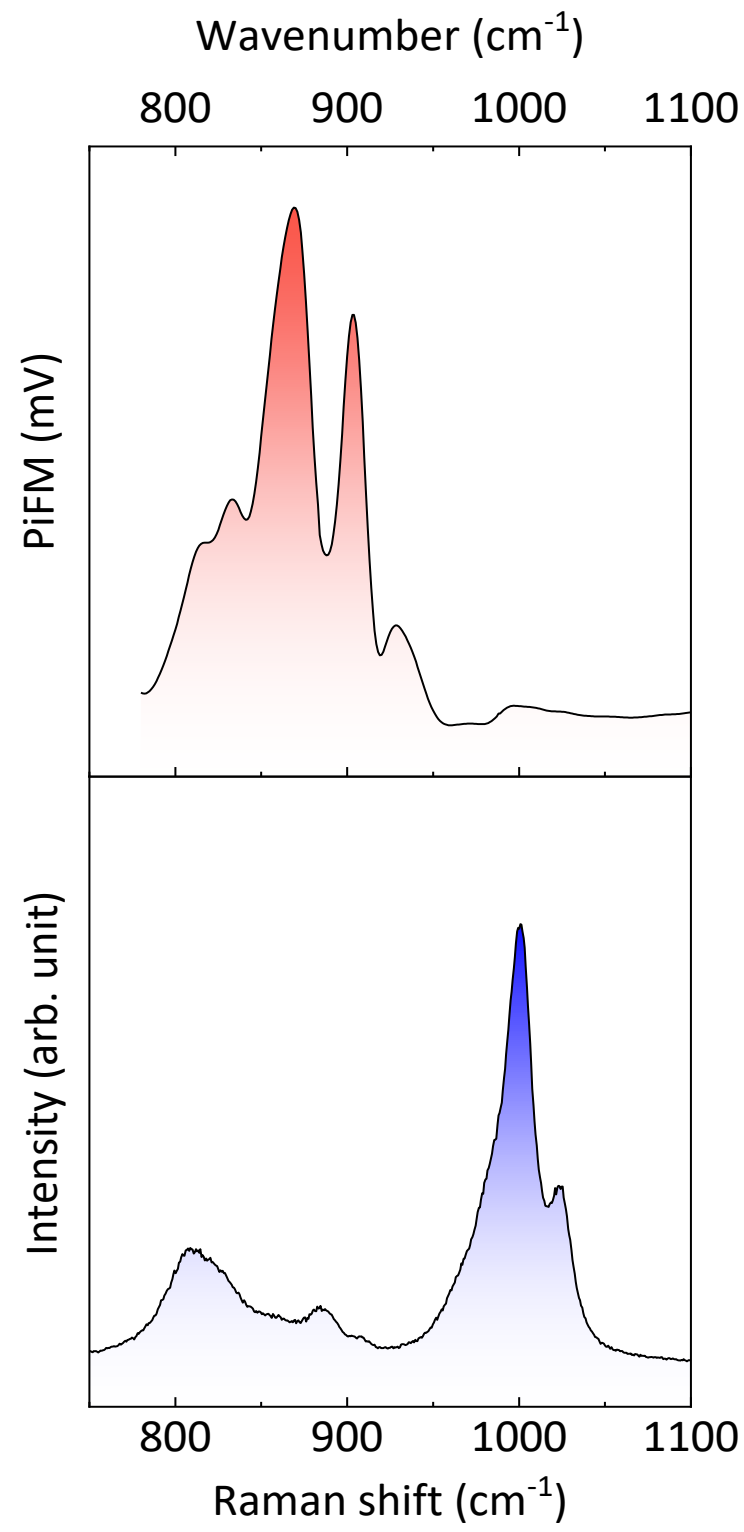

Supplementary Figure 6. Point PiFM spectrum (up panel) and Raman scattering spectrum (down panel) of the as-grown AMO flakes.

The PiFM spectrum was acquired by recording the PiF signal while tuning the laser wavenumber. It should be noted that the PiFM spectrum reported herein reflects the local infrared absorption at specific wavenumbers, is physically consistent with a nano-infrared (nano-IR) absorption spectrum[1, 2, 3, 4]. To account for the laser power variation across the tuning range, all spectra were normalized by the laser power profile, which was measured simultaneously during the scan.

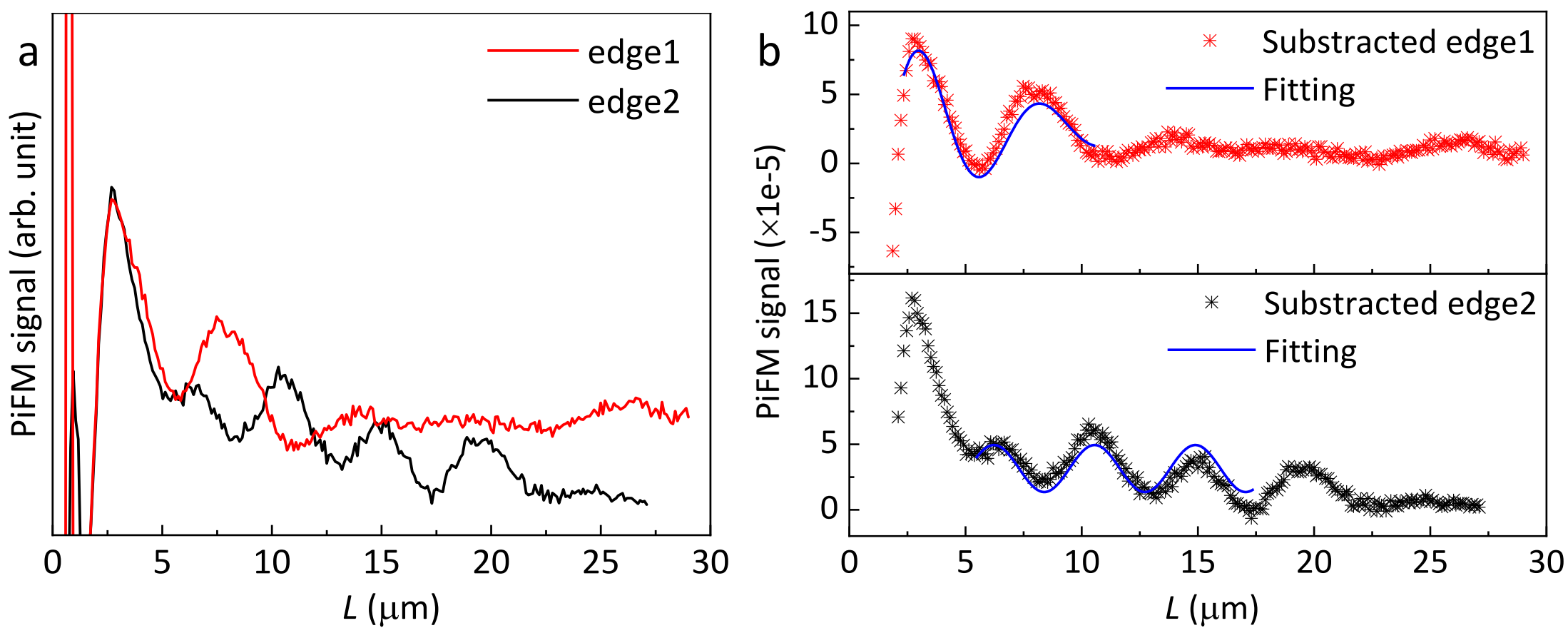

Supplementary Figure 7. (a) Detailed profiles taken from the edge1 (red) and edge2 (black) directions, the propagation periods are extracted to be λ_1 = 6.5 μm and λ_2 = 4.3 μm, respectively. (b) Fitting of the background-subtracted PiFM amplitude profiles using the following equation.

$$y(x) = y_0 + A_1 e^{-x/L_P} \sin\left(\frac{2\pi(x-x_c)}{\lambda_P}\right)$$

where y is the overall amplitude, y_0 is a vertical offset, A_1 is the amplitude of the edge-launched wave, L_p is the polariton propagation length, λ_p is the polariton wavelength, and x_c is the phase offset. The L_{p1} = 29.1 μm and L_{p2} = 20.9 μm can be extracted.

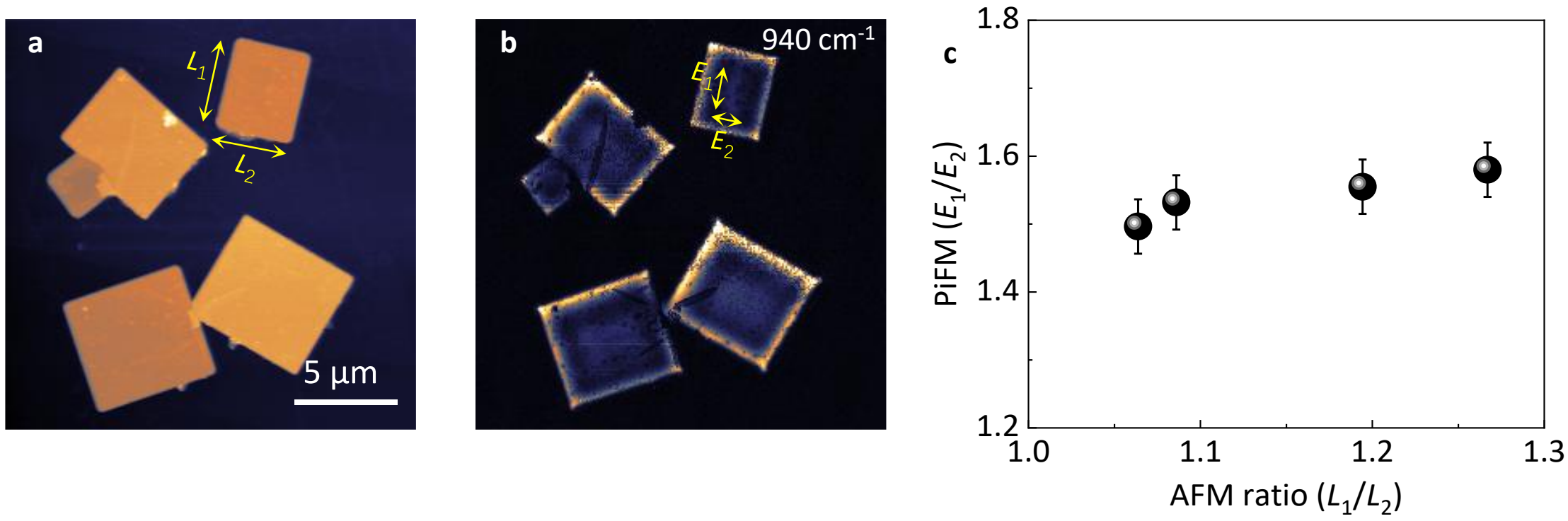

Supplementary Figure 8. (a) Atomic force microscopy (AFM) of AMO flakes. (b) The corresponding PiFM image at 940 cm^{-1}. (c) Aspect ratios of the central rectangular hot spots extracted from PiFM images (E_1/E_2) vs the corresponding geometric aspect ratios (L_1/L_2).

we performed additional PiFM measurements on a set of adjacent AMO nanoflakes that exhibit different in-plane orientations. Due to limitation of the maximum scan range of our PiFM setup, we selected four neighboring nanoflakes (Supplementary Figure 8a) that are sufficiently close to ensure comparable growth conditions, yet display distinct crystallographic orientations. PiFM images were acquired at a fixed excitation wavenumber of 940 cm^{-1}, as shown in Supplementary Figure 8b, the central rectangular-shaped hot spot rotates systematically with the orientation of each nanoflake. For four adjacent AMO nanoflakes, the aspect ratios of the central rectangular hot spots extracted from PiFM images (E_1/E_2, ranging from 1.49 to 1.58) are systematically larger than the corresponding geometric aspect ratios measured from AFM topography (L_1/L_2, ranging from 1.06 to 1.27). This discrepancy indicates that the observed shape anisotropy of the hot spot cannot be solely attributed to the flake geometry. Instead, it reflects an additional contribution from strain-induced in-plane dielectric anisotropy, which locally modifies the polariton focusing behavior.

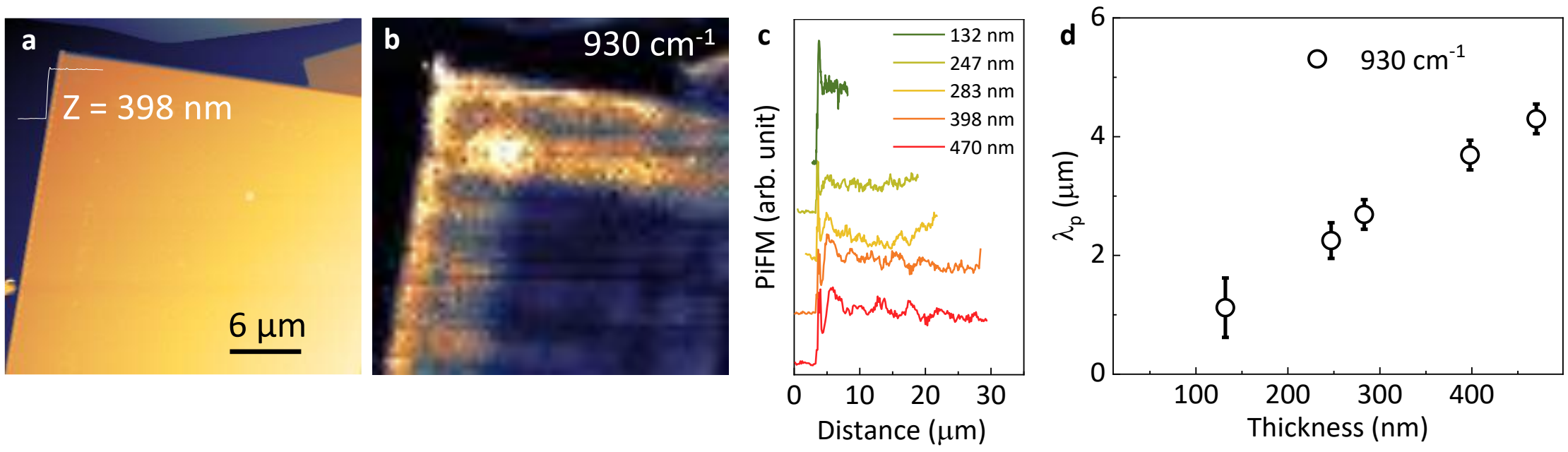

Supplementary Figure 9. (a) A typical AMO flake with a thickness t = 398 nm. (b) The corresponding PiFM image at 930 cm^{-1}. (c) Phonon polariton line profiles for AMO crystal with different thickness. (d) Wavelengths of phonon polaritons probed at 930 cm^{-1} for crystals with different thickness.

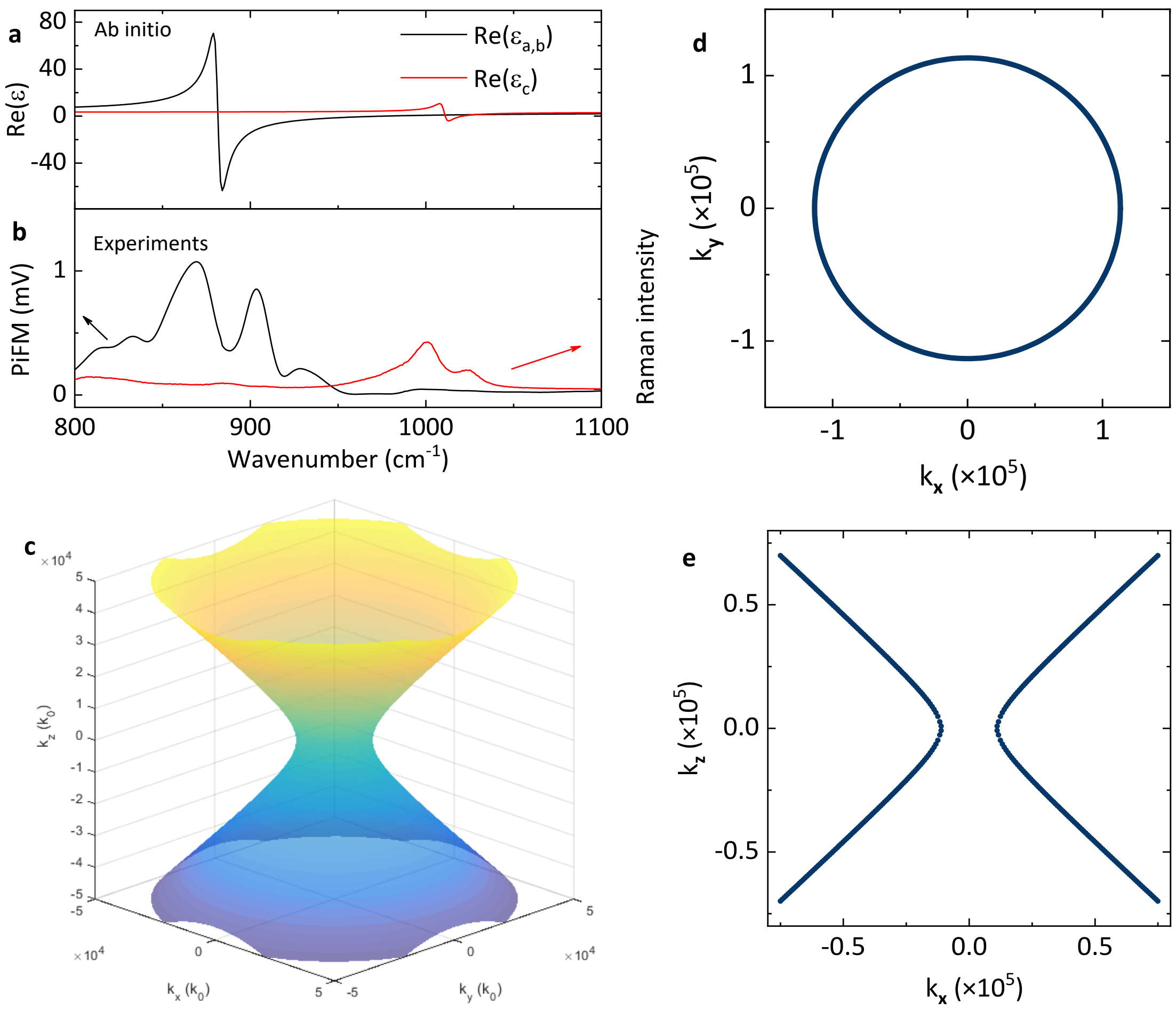

Supplementary Figure 10. (a) Ab initio permittivity of AMO crystal along the *ab*-[110] and *c*-[001] axes. (b) PiFM (black) and Raman scattering (red) spectra of the as-grown AMO crystal. (c) Isofrequency surfaces (IFC) defined by $\omega(k_\perp, k_\parallel) = \omega_0$ for a given frequency 930 cm^{-1}, in which $\varepsilon_{a,b} = -3.346$, $\varepsilon_c = 3.763$. (d) Projection of the IFC onto the k_x-k_y plane. (e) Projection of the IFC onto the k_x-k_z plane.

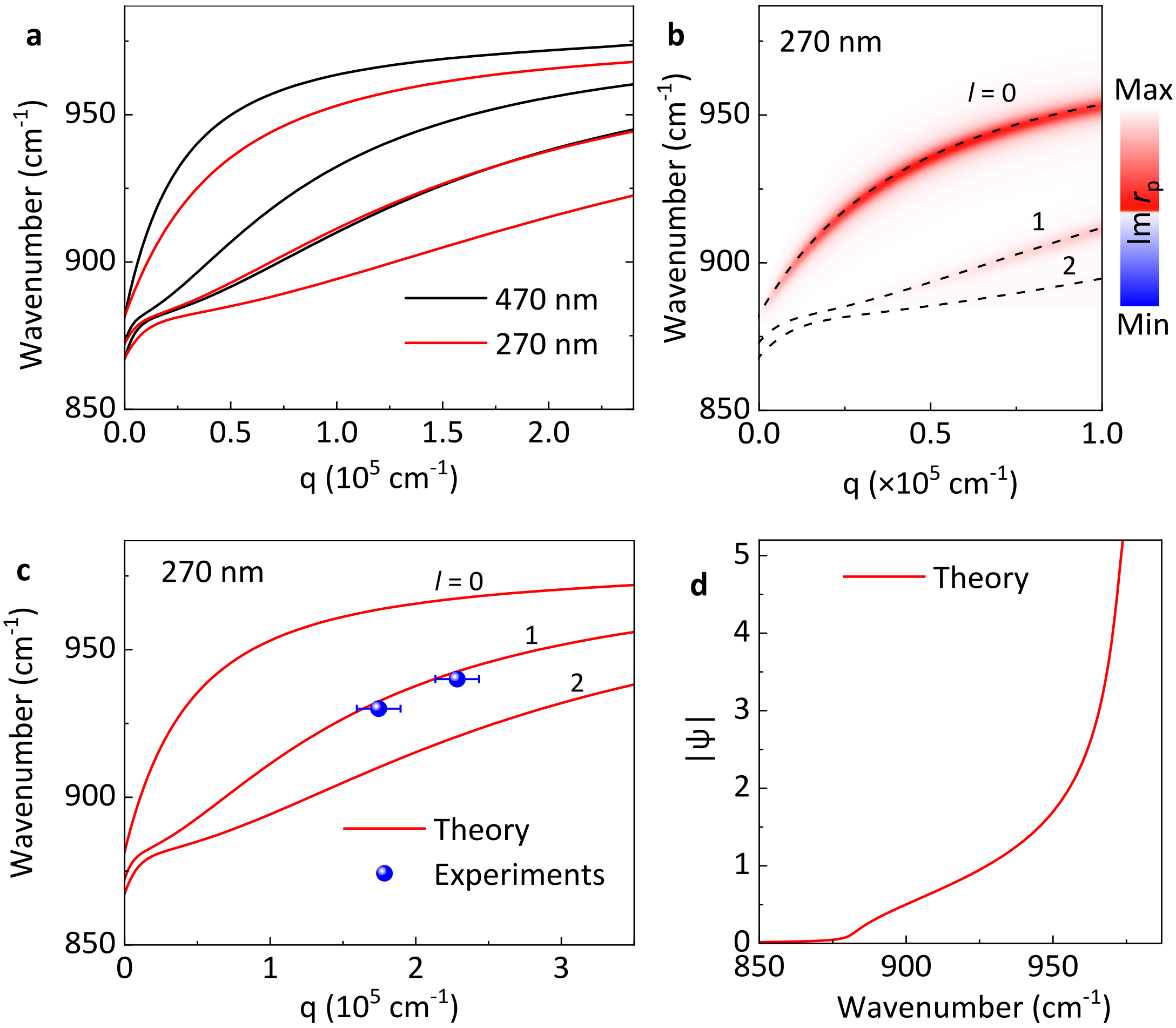

Supplementary Figure 11. (a) Calculated dispersion curves of AMO crystal with thickness d = 470 nm (black) and d = 270 nm (red) by numerical simulations. (b) Numerical (solid) vs. analytical (dashed) dispersion curves of the 270-nm-thick AMO crystal for l = 0, 1, 2 modes. The close agreement confirms the validity of the analytical polariton model. (c) Experimentally extracted l = 1 mode momenta (blue spheres) at 930 cm^{-1} and 940 cm^{-1} superimposed on the theoretical dispersion curves (solid) of the 270 nm-thick AMO nanoplate. (d) Theoretical hyperbolic figure of merit ψ as a function of wavenumber.

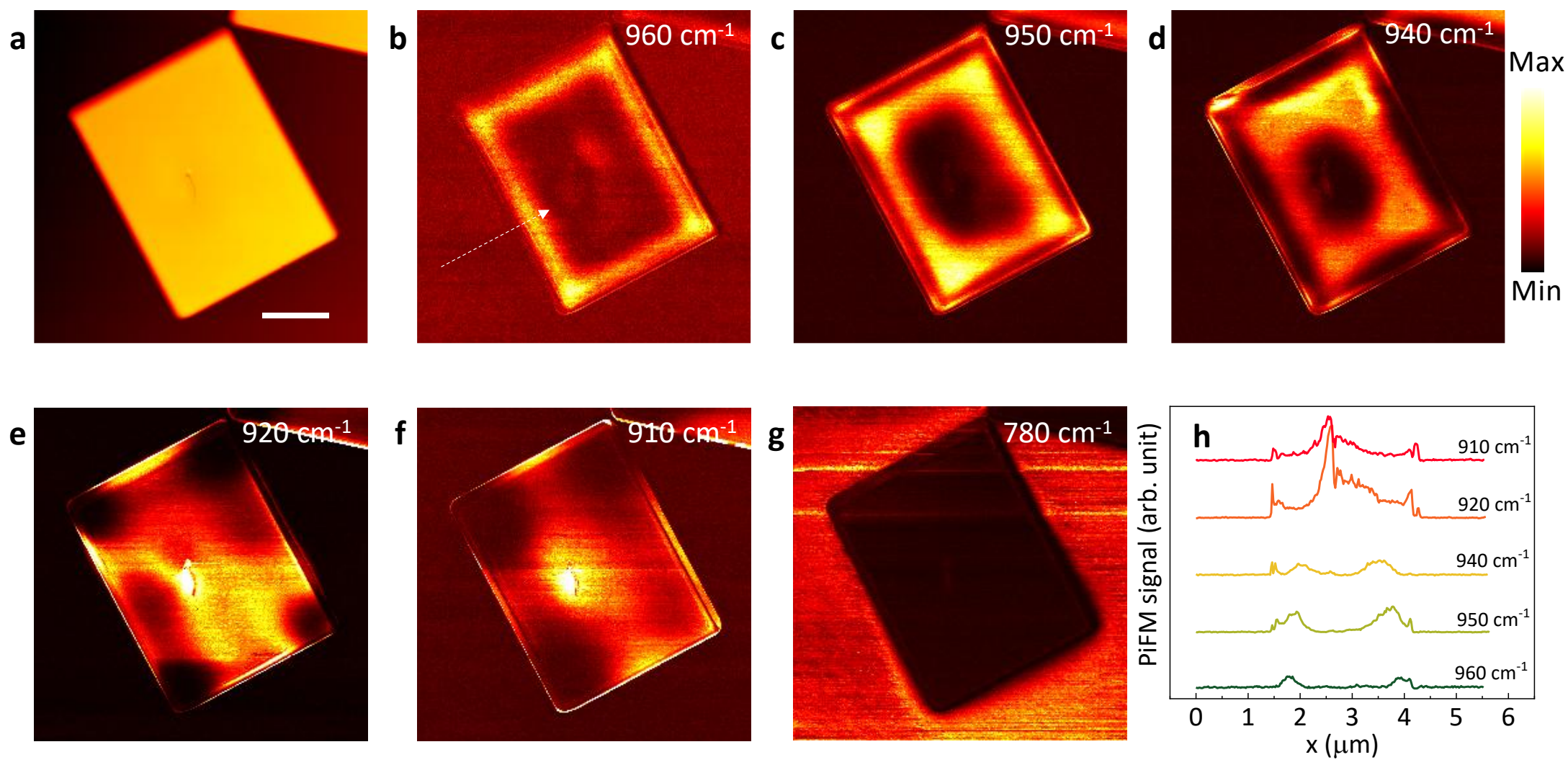

Supplementary Figure 12. (a) AFM image of an isolated single AMO crystal, scale bar is 1 μm. PiFM images of the AMO square for incident frequencies of 960 cm^{-1}, 950 cm^{-1}, 940 cm^{-1}, 940 cm^{-1}, 920 cm^{-1}, 910 cm^{-1}, and 780 cm^{-1}, respectively. (h) Amplitude line profiles extracted along the dashed line in the (b).

Supplementary Table 1. Comparison of PhP properties among AMO, $ATeMoO_6$, and Na/Li-V_2O_5

Parameters	$Al_2(MoO_4)_3$	Quaternary Oxides	Na/Li-V_2O_5
**Polariton Type**	Hyperbolic	Hyperbolic	Hyperbolic
**Anisotropy**	In-plane anisotropy	In-plane anisotropy	In-plane anisotropy
**Frequency Range**	900-970 cm^{-1}	860-950 cm^{-1}	948-1024 cm^{-1}
**Spectral Tunability**	Epitaxial strain/Lateral sizes	Cation substitution	Na intercalation
**Quality Factor**	4.86	6-10[5]	5[6, 7]
**Synthesis Methods**	Interfacial epitaxy	Chemical flux growth + exfoliation	Intercalation of Na/Li into V_2O_5 vdW crystals

Supplementary Table 2. The permittivity tensor via DFT calculations

	a,b-[110]					*c*-[001]			
**Band**	**TO**	**LO**	**Width**	**(Im)TO**	**Band**	**TO**	**LO**	**Width**	**(Im)TO**
**1**	346.7	365.6	18.9	31.2	**1**	293.6	299.7	6.1	21.6
**2**	407.3	410.6	3.3	12.2	**2**	371.5	384.4	12.9	33.7
**3**	461.1	464.4	3.3	31.3	**3**	453.8	486.1	32.3	70.6
**4**	474.1	525.4	51.3	34.3	**4**	505.7	535.9	30.2	20.9
**5**	530.4	543.5	13.1	3.1	**5**	874.2	883.0	8.8	45.1
**6**	842.3	845.2	2.9	20.0	**6**	909.8	997.8	88.0	84.1
**7**	881.5	976.2	94.7	66.9	**7**	1010.4	1021.6	11.2	1.8
**8**	997.9	1007.9	9.9	2.5					

Supplementary References

1. Wang L, Wang H, Vezenov D, Xu XG. Direct measurement of photoinduced force for nanoscale infrared spectroscopy and chemical-sensitive imaging. *J. Phys. Chem. C* **122**, 23808-23813 (2018).
2. Davies-Jones JA, Davies PR. Photo induced force microscopy: chemical spectroscopy beyond the diffraction limit. *Mater. Chem. Front.* **6**, 1552-1573 (2022).
3. Nowak D, Morrison W, Wickramasinghe HK, Jahng J, Potma E, Wan L*, et al.* Nanoscale chemical imaging by photoinduced force microscopy. *Sci. Adv.* **2**, e1501571 (2016).
4. Jahng J, Potma EO, Lee ES. Nanoscale spectroscopic origins of photoinduced tip–sample force in the midinfrared. *PNAS* **116**, 26359-26366 (2019).
5. Sun T, Chen R, Ma W, Wang H, Yan Q, Luo J*, et al.* Van der Waals quaternary oxides for tunable low-loss anisotropic polaritonics. *Nat. Nanotechnol.* **19**, 758-765 (2024).
6. Taboada-Gutiérrez J, Álvarez-Pérez G, Duan J, Ma W, Crowley K, Prieto I*, et al.* Broad spectral tuning of ultra-low-loss polaritons in a van der Waals crystal by intercalation. *Nat. Mater.* **19**, 964-968 (2020).
7. F. Tresguerres-Mata AI, Lanza C, Taboada-Gutiérrez J, Matson JR, Álvarez-Pérez G, Isobe M*, et al.* Observation of naturally canalized phonon polaritons in LiV_2O_5 thin layers. *Nat. Commun.* **15**, 2696 (2024).
