# Supplementary material for: Interfacial epitaxy of single-crystalline Al2(MoO4)3 flakes for anisotropic phonon polaritons
Source: Nat Commun. 2026 Jun 25;17:7956. doi: 10.1038/s41467-026-74681-x (PMC13448070; doi:10.1038/s41467-026-74681-x)
Supplement: Supplementary file 2 [file 41467_2026_74681_MOESM2_ESM.pdf]

nature portfolio

Peer Review File

Interfacial Epitaxy of Single-crystalline $Al_2(MoO_4)_3$ Flakes for Anisotropic Phonon Polaritons

Corresponding Author: Dr Chuangye Song

This file contains all reviewer reports in order by version, followed by all author rebuttals in order by version.

Version 0:

Reviewer comments:

Reviewer #1

(Remarks to the Author)
In this work, C. Song and coworkers showcase a unique synthesis method that realizes, for the first time, high quality single crystal flakes of aluminum molybdenate. The authors unveil polaritons in this material with a remarkable anisotropy that is plausibly tunable by strain or growth conditions.

I have four major concerns with this work that should all be addressed before it is suitable for publication in Nature Communications. In descending order of severity:

1) The authors introduce the AMO polaritons as “Hyperbolic phonon polaritons”, and beginning at line [67] somewhat consistently refer to them as such. Where is the evidence for hyperbolicity for these polaritons? At lines [230-232] authors state “as the excitation frequency increase … the polariton wavelength systematically decreases, a signature of type-II hyperbolic dispersion.” The observed “positive dispersion” is an insufficient condition to show hyperbolicity, and is consistent with conventional polariton media. In the related work (Ref. 27) by these authors, that evidence was supplied by the superlensing effect seen in polariton “hot rings” from a buried structure below the crystal. This effect owes to the superposition of discrete higher order polaritons that are unique to hyperbolic media. Whereas it could be difficult to observe such an effect for AMO, given the synthesis method presented here, the authors should provide some evidence for hyperbolicity. Can higher order polariton modes by observed near AMO crystal edges? A generous eye may find some hints in the images of Fig. 5, but higher order modes receive no discussion. At present, I see no clear evidence for hyperbolicity and the authors must remedy this or remove the “hyperbolic” claim.

2) The authors credit the impressive 33% anisotropy of AMO polaritons to in-plane biaxial strain which, if true, suggests tunability of the anisotropy. I do not see convincing evidence that polariton anisotropy owes to biaxial strain. Were this so, I might expect crystals e.g. in Fig. 1c to showcase variable polariton anisotropy relative to the AMO crystal axes, since these crystal are presumably differentially strained upon the sapphire substrate. Can the authors convincingly identify any variation in polariton anisotropy that could be associated to “tunable” strain? Otherwise, I find it more plausible that optical anisotropy in oscillator strength of the Mo-O stretch mode, allowed by the crystal symmetry, could induce observed polariton anisotropy. Such anisotropy in Raman-active modes is already showcased in Fib. 3b, which credits this scenario. Like issue 1), this controversy could be settled with a measurement, prediction, or at least credible reference to the anisotropic optical constants of AMO. Alternatively, if authors can transfer an AMO crystal from sapphire onto a substrate where strain is mitigated, does the anisotropy change or vanish?

3) Table 1 is interesting but problematic in several ways that must be addressed. As mentioned, “hyperbolic type” for AMO is unsubstantiated. “Spectral tunability” for each material under comparison is indicated as “high”; unless a quantitative metric can be supplied, this descriptor should be removed. Certain details of the table are either confusing or incorrect. For instance, hBN is indicated to enjoy spectral tunability through “electrostatic doping”. The ionic solid is an insulator; what electrostatic tuning is referenced? Broadly, a reference should be added for each quoted tuning mechanism.

4) The manuscript should be augmented to present more technical detail regarding measurement methods used. Nature Communications articles conventionally include a “Methods” section and this should be the case for the present submission in order to explain, for example, the following: How were the X-ray diffraction results presented in Fig. 1f acquired? Are these area-averaged measurements from a sapphire substrate with sparse coverage of AMO? What diffractometer was used?

What PiFM instrument and laser were used to acquire the nano-imaging results shown throughout the article? What "signal", explicitly, is imaged in e.g. Fig. 1b? I can infer that these details are provided in Ref. 27, but a similarly detailed explanation of PiFM methods should be provided in the present manuscript. More detail should be supplied to explain how the "nano-infrared absorption" spectrum of Fi[g]ure S4 was acquired. Is this truly an absorption spectrum, or a PiFM spectrum? Discussion of PiFM methodology should include an explanation of normalization of spectra, if applicable.

Provided that the above major concerns can be addressed, I suggest the authors address the following matters to improve the quality of the manuscript:

* The authors quote the maximum propagation length for polaritons in AMO. Since this figure clearly depends on the polariton wavelength, which itself varies with energy and also (presumably) AMO thickness, it is a less useful comparative metric than the polariton quality factor. Can the authors include this metric in comparison to the values for "alternative" materials in their Table 1?
* Polaritons in several AMO crystals of different thickness can be identified in the images. How does the observed polariton wavelength depend ("tunably") on crystal thickness?
* The concept of "formation energy above hull" (Fig. 1g) will be unfamiliar to most of the general readership of this journal and should be given more explanation. Furthermore, how were the data points in Fig. 1g predicted? If these were obtained by novel electronic structure calculations, then calculation details should be provided. If values are taken from literature, references should appear in the Figure and/or its caption.
* The discussion of modal evolution with frequency in the AMO crystal shown in Fig. 5 is interesting, but what is the main conclusion of this analysis? If the answer is the remarkable confinement (Fig. 1d), can the authors rationalize its scale? Can the authors provide a simple simulation to rationalize these images? And further, can the authors hypothesize why polariton images show significant asymmetry within these apparently symmetric crystals?
* EDX mapping in Fig. 2b appears to show an excess of oxygen at crystal edges; can the authors assess whether this is a genuine feature of these crystals, and whether it could have any impact on polaritons "launched" from edges?

The following minor issues were also identified:
* Line [175] of the manuscript asserts "the resulting AMO structure … is schematically illustrated." Where?
* Line [252] of the manuscript references Fig. 5b, then puzzlingly refers to an irrelevant panel in Fig. 4b.

This polaritonic medium and its accessible synthesis method will be of broad interest to the photonics community. But major claims of the manuscript must be substantiated with more evidence before it is acceptable for publication.

Reviewer #2

(Remarks to the Author)
In this manuscript, the authors present a novel interfacial epitaxy method for synthesizing 2D single-crystalline $Al_2(MoO_4)_3$ (AMO). This strategy consists in putting a molybdenum foil in between two sapphire wafers and heating them up in air atmosphere. Once synthesized, the authors thoroughly characterize the AMO single crystals on sapphire by Raman spectroscopy, X-ray diffraction, and scanning transmission electron microscopy (STEM). The authors then study AMO's mid-IR near-field optical response by performing photo-induced force microscopy (PiFM) scans to map the phonon-polariton (PhPs) dispersion in AMO. In my opinion, the manuscript, in its current form, requires major revision. While the synthesis and structural characterization of 2D single-crystalline $Al_2(MoO_4)_3$ (AMO) are interesting, the work lacks sufficient theoretical, analytical, and comparative support, particularly regarding the near-field polaritonic experiments and the positioning of AMO within the broader family of mid-infrared polaritonic materials.

In particular:

1. The authors must include a detailed Methods section in which all experimental procedures are described comprehensively. The experimental details of the PiFM near-field measurements should be explained thoroughly to ensure reproducibility.

2. In Figure 1 panel c, there is a missing "Z" or similar to denote the magnitude that is plotted in this panel (topography).

3. The FFT resolution of the experimentally extracted polariton dispersion in Fig. 4 is notably low, which limits the accuracy of the reported wavevector–frequency relationship. The authors should improve the FFT resolution and clarify how this limitation affects the extracted polaritonic parameters.

4. The authors employ phrases such as "critically needed," "fills a critical gap," and "highly promising candidate" multiple times in the manuscript. The authors should soften them to maintain a balanced and objective tone consistent with the level of evidence provided.

5. The manuscript frequently refers to AMO flakes with thicknesses of 50–500 nm as "two-dimensional (2D)". In the context of nanophotonics and polaritonics, this terminology is typically reserved for ultrathin or quasi-2D materials. The authors should reconsider this terminology (for instance, use single thin layers).

6. The authors state: "Moreover, the polaritonic modes in AMO flakes are exclusively observed within a narrow frequency window (900–970 cm^{-1}), complementing previously reported PhP propagation bands in van der Waals materials such as h-

BN31 an α-MoO332, 33, 34 (Table 1). This distinct spectral band thereby fills a critical gap in the mid-infrared polaritonic landscape, extending the accessible frequency window for nanophotonic and optoelectronic applications.", but the said spectral regime widely overlaps with α-MoO3 Reststrahlen band. The authors must temper this claim and clarify what uniquely distinguishes AMO beyond its specific spectral window.

7. The authors state: "The synthesis of high-quality, single-crystalline $A_2(MoO_4)_3$ is therefore critically needed to unlock fundamental studies of anisotropic polaritonic phenomena and to enable prospective photonic and optoelectronic applications." The claim that the synthesis of single-crystalline AMO is "critically needed" to unlock polaritonic studies is too strong, since in its Restrahlen Band overleap with other mid-IR polaritonic materials. Moreover, the generality of the synthesis approach is not demonstrated beyond AMO. The authors must rewrite this part with a more balanced tone and clearer positioning relative to existing materials.

8. There are some relevant references of pioneering polaritonic works in hBN (Science 2014, 343, 1125-1129) and in α-MoO3 (Nature 2018, 562, 557–562) missing.

9. The AMO crystals are compared to benchmark polaritonic materials such as h-BN and α-MoO3 (Table 1). However, these materials are typically obtained as atomically flat, exfoliated flakes. The AMO flakes reported here have thicknesses between 50 and 500 nm and no quantitative surface roughness analysis is provided. A systematic AFM roughness study (RMS roughness over representative areas) is necessary to assess whether the surface quality is suitable for low-loss nanophotonic applications and to enable a fair comparison with established 2D polaritonic materials.

10. Some parameters listed in Table 1 (e.g., propagation length and tunability) are presented without specifying comparable experimental conditions such as substrate, thickness, or excitation frequency. The authors must clarify these conditions to improve the usefulness of the comparison.

11. The manuscript currently compares AMO only with h-BN and α-MoO3. Given the rapidly expanding family of mid-IR polaritonic materials, a broader comparison is necessary. The authors should contextualize AMO against other recently reported systems, such as quaternary oxides (Nat. Nanotechnol. 2024, 19, 758–765), Li- and Na-V2O5 (Nat. Commun. 2024, 15, 2696 and Nat. Mater. 2020, 19, 964–968), among others, using consistent polaritonic metrics.

12. The authors state "The polaritonic propagation length Lp = 20.9 μm is two times larger than that of α-MoO3 flakes (Table1).". As noted above, meaningful comparisons of polaritonic figures of merit require measurements performed under comparable conditions, for instance when the materials are placed on mid-IR transparent substrates. In addition, the main text manuscript does not clearly specify along which in-plane crystallographic direction this propagation length is extracted, which is particularly important given the anisotropic nature of the system.

13. The authors state "Line profiles (Figure 5c,d) quantify this evolution, showing a systematic reduction in fringe spacing as the frequency decreases. The central hotspot at 930 cm^{-1} has a full width at half maximum (FWHM) of approximately 633 nm, yielding a confinement factor ($\lambda IR/\lambda p$) of about 17—comparable to the value (50) previously reported for microcavity-modulated α-MoO_3 nanosheets.30". However, in the current manuscript, ref. [30] corresponds to Duan et al., Adv. Mater. 2022, 34, 2104954, which is based on h-BN nanoantennas rather than α-MoO3 nanosheets. The authors must carefully verify all references to ensure that they correctly support the corresponding statements in the text. In addition, and as mentioned above, comparisons with other polaritonic materials should only be made under comparable experimental conditions (e.g., similar substrate, thickness, and geometry).

14. Throughout the manuscript, the authors directly interpret anisotropic polariton propagation as hyperbolic behavior. However, anisotropy alone does not imply hyperbolicity. The authors should explicitly demonstrate hyperbolic dispersion by extracting isofrequency curves (IFCs) or by determining the sign of the principal components of the dielectric tensor. Without this, the classification of the observed PhPs as hyperbolic remains ambiguous. In particular, the experimentally extracted dispersion from Fig. 4d suggests elliptical PhPs. The authors must clarify this.

15. If AMO is to be proposed as a polaritonic material, a more exhaustive experimental and theoretical analysis is required. Exhaustive investigations of key polaritonic figures of merit—such as propagation length, group velocity, and lifetime—are currently missing. In addition, the authors should analyze the frequency-dependent complex PhP phasor along the [100] and [010] crystallographic directions (see, e.g., Nature Photonics 2015, 9, 674–678, Fig. 2cf and Nano Letters 2025, 25, 6, 2203–2209, Fig. 3g) to determine whether the observed anisotropic PhPs are hyperbolic or elliptical.

16. The PiFM results are not supported by analytical or numerical modeling. Given that AMO is biaxial and sapphire permittivity is well known, the authors could model the PhP dispersion using established analytical frameworks (e.g., Phys. Rev. B 2019, 100, 235408) or FEM simulations. Such modeling is essential to validate the extracted dispersions and to confirm the claimed hyperbolic behavior.

17. Since the authors propose AMO as a new mid-IR nanophotonic material, Raman spectroscopy alone is insufficient. FTIR spectroscopy should be performed to identify the transverse optical (TO) and longitudinal optical (LO) phonon modes. This information is essential to extract and fit the infrared dielectric permittivity tensor of AMO, which is a prerequisite for any quantitative polaritonic analysis.

18. All near-field experiments are performed on sapphire substrates, which exhibit strong phonon resonances in the same spectral range as the reported AMO Reststrahlen band. This might potentially induce substrate-induced artificially enhanced

propagation lengths. The authors should clarify whether the AMO crystals can be mechanically picked up and transferred onto alternative substrates commonly used in mid-IR nanophotonics (e.g., Si, SiO2, CaF2, BaF2). If transfer is feasible, the authors should perform PiFM measurements on mid-IR transparent substrates (CaF2 or BaF2) to isolate the intrinsic polaritonic properties of AMO.

19. A very important note: The tunability attributed to biaxial strain and lateral size in Table 1 is not supported by convincing experimental or theoretical evidence. Strain is inferred from epitaxial mismatch but is neither measured nor varied, and no strain-dependent phonon or polariton shifts are reported. Additionally, the influence of lateral size is discussed without separating it from thickness effects. In its current form, the table suggests a level of material tunability that is not demonstrated in the manuscript.

20. In the discussion of Fig. 4, the authors refer to the observation of "edge modes," based on the fringe patterns visible in the panels of Fig. 4b. However, the features observed in these panels correspond to edge-launched or edge-reflected phonon polaritons rather than true edge modes (for real edge-modes see Nature Photon 2016, 10, 239–243). The authors should revise the text accordingly and use the correct terminology (see, e.g., Nano Letters 2017, 17, 9, 5285–5290). In addition, the authors should carefully check the fitting procedures of the near-field line profiles reported in the Supporting Information.

21. While edge-launched PhP are observed, their frequency dependence and physical origin are not sufficiently analyzed. A systematic study of PhP dispersion and its dependence on strain and crystal orientation is needed. At present, the role of strain in shaping edge modes remains qualitative.

22. Sapphire is not a standard substrate for nanophotonic device integration. If AMO flakes are transferred to other substrates, it is unclear whether the epitaxial strain—and thus its reported influence in anisotropic polaritonic behavior—would be preserved. This point should be discussed to assess the practical relevance of strain-engineered AMO.

In its current form, the manuscript does not yet meet the scientific standards required for publication in Nature Communications. Substantial additional experimental and theoretical work is necessary to support the central claims, particularly regarding the polaritonic properties and their physical interpretation. However, if the authors thoroughly address all the points raised above and significantly strengthen the manuscript, the work could become suitable for publication in Nature Communications.

Reviewer #3

(Remarks to the Author)
The authors present an efficient method for synthesizing thin layers of aluminium molybdate (AMO), provide a detailed characterization of these layers, and demonstrate the propagation of phonon polaritons (PhPs) within layers of this material. The paper is solid, well-written, and clear. The section on the synthesis and basic characterization of the material provides a comprehensive description of the synthesis procedure, leaving no doubt regarding the structure and quality of the resulting samples. However, the final section, devoted to the study of PhPs in AMO, requires some revision. Overall, I believe the paper is interesting and valuable to the scientific community. However, the following questions should be addressed before the paper can be accepted for publication.

1. The authors claim that the in-plane anisotropy of phonon polaritons is related to the presence of in-plane compression and tension of the material along different directions (specifically on page 13, lines 234-236). However, in my opinion, this statement does not appear fully substantiated. Indeed, the AMO itself is already structurally anisotropic in the plane; consequently, optical anisotropy can be expected even in the absence of strain, as in other biaxial materials. Therefore, it remains unclear to what extent the anisotropy of phonon polariton propagation is due to structural anisotropy and to what extent to the presence of strain.
2. In fact, the observed fringe period does not always correspond to the polariton wavelength. The error arises when the polariton wavelength is comparable to the wavelength in vacuum, which is the case in this study, unlike many other mid-IR experiments, where the polariton wavelength can be tens of times shorter than the free-space wavelength. With such a long wavelength, the sample irradiation direction can influence the fringe length, thereby increasing or decreasing the observed anisotropy. A method for correctly determining the polariton wavelength, taking into account the beam incidence direction, is described in the following paper: Nature Photon 11, 356–360 (2017). These corrections may not be very large in this case, but it is reasonable to estimate them.
3. The obtained data allows to estimate the principal components of the in-plane permittivity tensor. Indeed, in the two-dimensional conductivity (ultrathin layer) approximation (Nature 562, 557–562 (2018), Phys. Rev. B 100, 235408 (2019)), the out-of-plane component of the permittivity tensor can be eliminated from the dispersion equation, allowing for a unique relationship between the wavevector and the in-plane component of the permittivity tensor. This result may help substantiate the nature of in-plane anisotropy.
4. On page 13, line 232, reference 29 seems to be misplaced, because the cited paper describes other types of polaritons, surface polaritons. The authors probably wanted to mention uniaxial hyperbolic material types, described, e.g., in Nat Commun 5, 5221 (2014). Furthermore, since the material is obviously not uniaxial, this classification should be used with caution and with appropriate comments.
The following terms are used incorrectly:
5. On page 13, line 223, the observed modes are called edge modes; however, these are not edge modes, but in-plane modes excited by the edge. Edge modes are modes that propagate along the edge, see, for example, Nano Lett. 2017, 17, 1, 228–235.

6. Fringes, observed in Fig. 4, are not resonant peaks (page 13, line 230) because they do not correspond to any resonances; they are just interference maxima formed by the edge-launch polaritons fields and by the field of the tip.
7. The use of the term "two-dimensional" in the context of the resulting flakes can be misleading. Flakes thicker than 100 nm are far from being monoatomic layers and are not two-dimensional for most of the physical processes occurring within them.

Reviewer #4

(Remarks to the Author)
I co-reviewed this manuscript with one of the reviewers who provided the listed reports. This is part of the Nature Communications initiative to facilitate training in peer review and to provide appropriate recognition for Early Career Researchers who co-review manuscripts.

Version 1:

Reviewer comments:

Reviewer #1

(Remarks to the Author)
The authors have addressed in essence all the concerns I first expressed about the manuscript and its conclusions. Most essential is the convincing presentation of the identified polaritons as hyperbolic in nature.

There are in my view two remaining important issues to address:

First, the authors' brief added discussion of higher order polaritons perhaps identifiable as short-wavelength excitations near crystal edges is important and, therefore, perhaps too brief. The authors should make an effort to identify the wavelength of these putative high-order modes, for at least a few energies, so that they can be added to the authors' theoretical hyperbolic dispersion. The presented theoretical permittivity tensor makes possible to predict the wavevector of l=1 modes which are presumably detectable in the experiment. Notably, the separation of l=0 and l=1 mode momenta is a direct reporter of "psi", which is an important figure of merit for hyperbolicity in connection to the angle of polariton ray propagation. With such expanded quantitative discussion of these higher order modes, and their importance for hyperbolic "beaming", the hyperbolic claims of the manuscript will be adequately substantiated.

Second, the authors should expand their discussion of the unique merits of their epitaxial hyperbolic medium, in contrast to exfoliated hyperbolic crystals. The tabular comparisons to alternate materials are useful, but leave unaddressed the technological need for and progress towards large-area single-crystalline media. What are the foreseeable prospects for large-area single crystalline growth of this hyperbolic medium, especially in comparison to ongoing efforts towards CVD growth of e.g. hBN? Could large-area single crystalline sheets be obtained, and by what modified synthesis approach? Given the authors' new experience with materials transfer, what are the prospects and potential solutions for transfer of this medium to alternate optical substrates? Addressing these questions will better establish the significance of this new epitaxial hyperbolic medium.

With these substantial modifications, I believe the work could deserve interest by the broad readership of Nature Communications.

Reviewer #2

(Remarks to the Author)
The authors have adequately addressed my previous concerns. The manuscript has improved substantially compared to the initial submission, and I believe it is now suitable for publication in Nature Communications in its current form.

Reviewer #3

(Remarks to the Author)
I have carefully reviewed the responses to my questions and comments. I believe the authors have done a very high-quality job, convincingly arguing their answers to the issues I raised. I am confident that, after the corrections and improvements have been made, the paper can be published in Nature Communications.

Reviewer #4

(Remarks to the Author)
I co-reviewed this manuscript with one of the reviewers who provided the listed reports. This is part of the Nature Communications initiative to facilitate training in peer review and to provide appropriate recognition for Early Career Researchers who co-review manuscripts.

Open Access This Peer Review File is licensed under a Creative Commons Attribution 4.0 International License, which permits use, sharing, adaptation, distribution and reproduction in any medium or format, as long as you give appropriate credit to the original author(s) and the source, provide a link to the Creative Commons license, and indicate if changes were made.
In cases where reviewers are anonymous, credit should be given to 'Anonymous Referee' and the source.
The images or other third party material in this Peer Review File are included in the article's Creative Commons license, unless indicated otherwise in a credit line to the material. If material is not included in the article's Creative Commons license and your intended use is not permitted by statutory regulation or exceeds the permitted use, you will need to obtain permission directly from the copyright holder.
To view a copy of this license, visit https://creativecommons.org/licenses/by/4.0/

Response Letter for No. NCOMMS-25-100045

Dear editors,

We thank you and the reviewers for your consideration and careful evaluation of our manuscript (No. NCOMMS-25-100045, entitled "Interfacial Epitaxy of Single-crystalline Al2(MoO4)3 Flakes for Anisotropic Phonon Polaritons"). The constructive comments and valuable suggestions, particularly regarding the role of strain, and the properties of the observed phonon polaritons, have considerably improved the quality of our manuscript. The manuscript has been revised accordingly, and all changes are highlighted in yellow in the revised version. The point-by-point responses are listed as follows.

For Reviewer #1:

Reviewer #1: C. Song and coworkers showcase a unique synthesis method that realizes, for the first time, high quality single crystal flakes of aluminum molybdenate. The authors unveil polaritons in this material with a remarkable anisotropy that is plausibly tunable by strain or growth conditions. I have four major concerns with this work that should all be addressed before it is suitable for publication in Nature Communications.

Our response: We thank the reviewer for their positive feedback on our manuscript. We have addressed all the concerns raised by the reviewer, which helped us improve the manuscript substantially. Below we provide a point-by-point response.

Comment 1: The authors introduce the AMO polaritons as "Hyperbolic phonon polaritons", and beginning at line [67] somewhat consistently refer to them as such. Where is the evidence for hyperbolicity for these polaritons? At lines [230-232]

authors state “as the excitation frequency increase … the polariton wavelength systematically decreases, a signature of type-II hyperbolic dispersion.” The observed “positive dispersion” is an insufficient condition to show hyperbolicity, and is consistent with conventional polariton media. In the related work (Ref. 27) by these authors, that evidence was supplied by the superlensing effect seen in polariton “hot rings” from a buried structure below the crystal. This effect owes to the superposition of discrete higher order polaritons that are unique to hyperbolic media. Whereas it could be difficult to observe such an effect for AMO, given the synthesis method presented here, the authors should provide some evidence for hyperbolicity. Can higher order polariton modes by observed near AMO crystal edges? A generous eye may find some hints in the images of Fig. 5, but higher order modes receive no discussion. At present, I see no clear evidence for hyperbolicity and the authors must remedy this or remove the “hyperbolic” claim.

Our response: We thank the reviewer for the time and effort spent on evaluating our manuscript and sincerely appreciate the reviewer’s concerns about the hyperbolic nature of the phonon polaritons in AMO crystal, which are highly constructive and have helped us in performing additional analyses that greatly strengthened the paper.

In response to the reviewer’s concern regarding the demonstration of hyperbolic behavior in our phonon polaritons, we have calculated the permittivity tensor using DFT method (Figure **R1a**). The resulting ab initio permittivity function qualitatively agrees with the experimental PiFM and Raman scattering spectra (Figure **R1b**). There is obvious discrepancy between the *ab*-[110] and *c*-[001] axes in the range of 800-1100 cm^{-1}. The isofrequency surfaces in Figure **R1c**, defined by $\omega(k_{\perp}, k_{\parallel}) = \omega_0$ for a given frequency 930 cm^{-1}, indicates the hyperbolic nature of AMO crystal, i.e., in-plane elliptical phonon polaritons (Figure **R1d**) and out-of-plane hyperbolic phonon polaritons (Figure **R1e**).

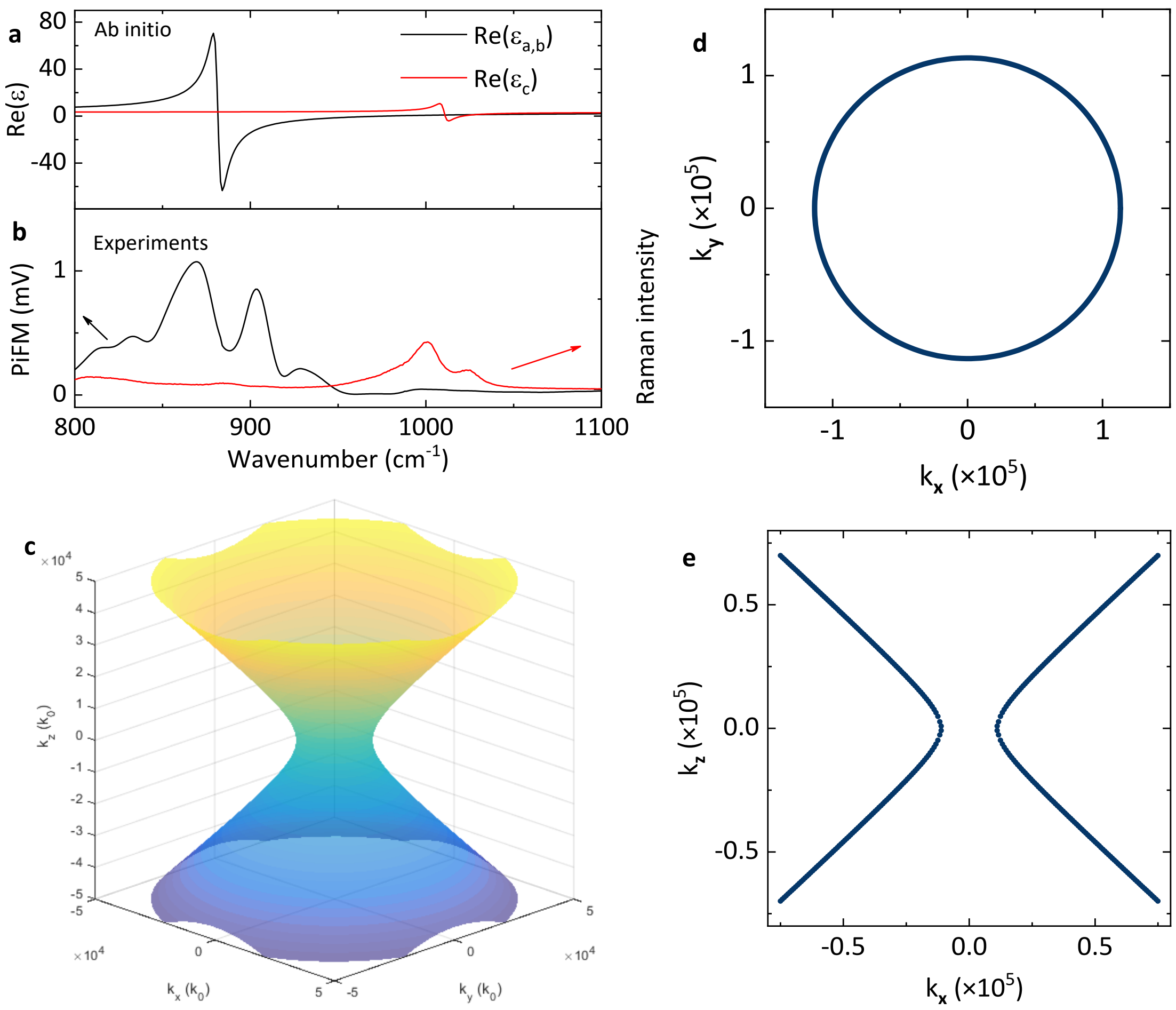

Figure R1. (a) Ab initio permittivity of AMO crystal along the *ab*-[110] and *c*-[001] axes. (b) PiFM (black) and Raman scattering (red) spectra of the as-grown AMO crystal. (c) Isofrequency curves (IFC) defined by $\omega(k_\perp, k_{//}) = \omega_0$ for a given frequency 930 cm^{-1}, in which $\varepsilon_{a,b}$ = -3.346, ε_c = 3.763. (d) Projection of the IFC onto the k_x-k_y plane. (e) Projection of the IFC onto the k_x-k_z plane.

We have also conducted an in-depth analysis of the PiFM measurements presented in Figure **5** and Figure **S11**, which could provide compelling experimental evidence. As shown in **Figure R2**, the observed evolution of the near-field pattern with decreasing excitation wavenumber, initially, the edges of the square nanoislands light up at 960 cm^{-1} due to efficient polariton launching. Subsequently, a bright ring forms and progressively shrinks at 950 cm^{-1}, eventually converging into a single, intense hot

spot at the center of the flake (940 and 930 cm^{-1}). In addition, we have now highlighted a representative region near the left edge of the AMO flake with yellow dashed rectangles in Fig. R2. Within these areas, one can clearly resolve fringes with a distinctly smaller periodicity compared to the fundamental mode, which is characteristic of higher-order guided polariton modes. This assignment is consistent with the established understanding of hyperbolic polaritons in anisotropic layered materials. For instance, Dai et al. [Nat. Commun. 6, 6963 (2015)] observed multiple guided modes (up to $l = 2$) in hexagonal boron nitride by analyzing near-field interference patterns near sample edges, where each mode index corresponds to a quantized out-of-plane momentum and manifests as a distinct spatial periodicity in real-space images. Following the same rationale, the short-period fringes we observe near the AMO edge can be attributed to the excitation and reflection of higher-order polariton modes (e.g., $l = 1, 2, \ldots$). These modes have larger in-plane wavevectors and thus produce shorter oscillation periods, especially prominent in regions where the tip is very close to the edge. We have now added a brief discussion of this point in the revised manuscript, page 16, line 303, "*The fringes highlighted by yellow dashed rectangles, which exhibit a distinctly smaller periodicity than the fundamental mode, are most likely attributable to higher-order polariton modes*".

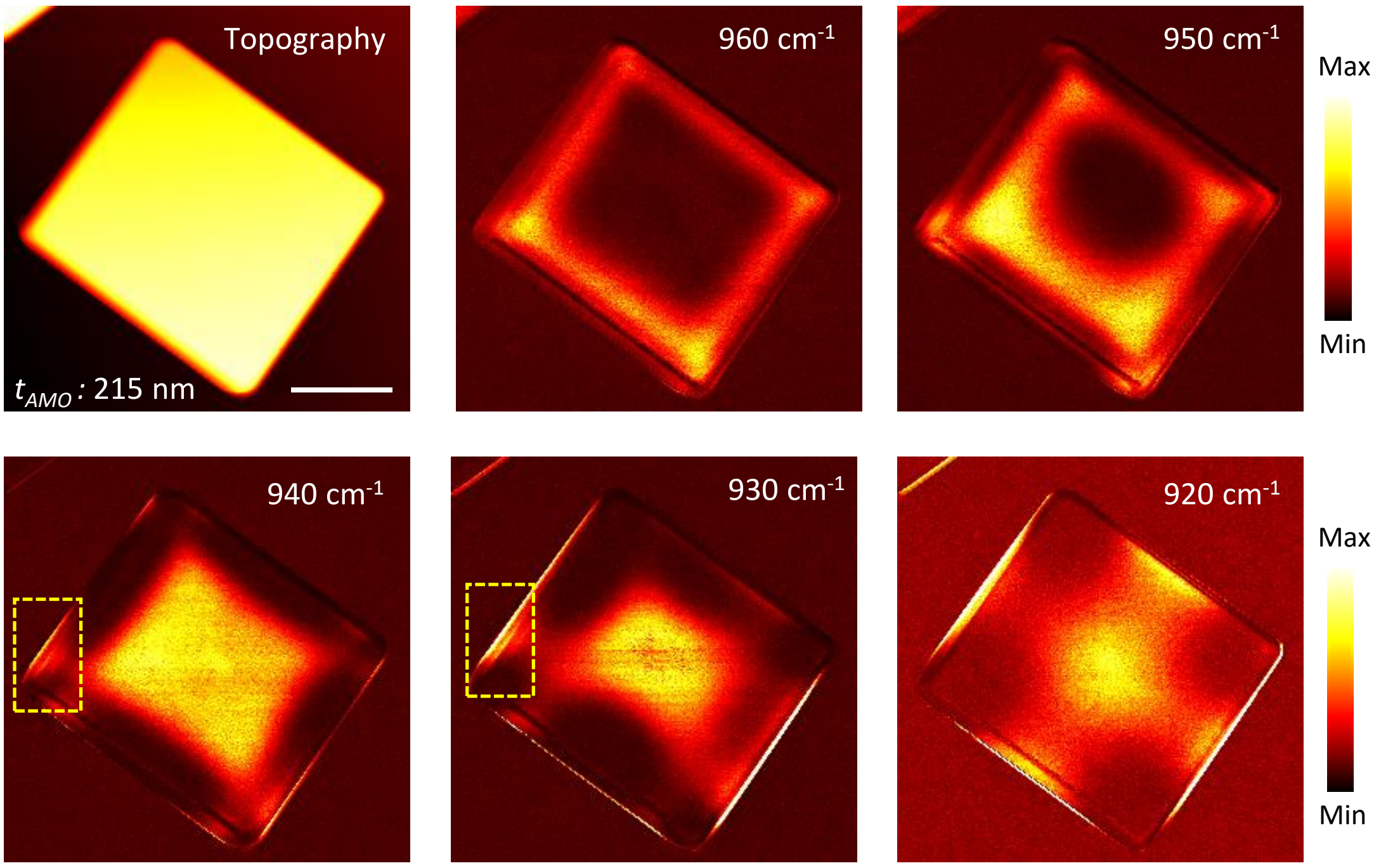

Figure R2. AFM and PiFM images (4 × 4 μm^2) of a square AMO crystal with incident frequencies from 960 to 920 cm^{-1}. Scale bar, 1 μm. Thickness of the AMO flake is t = 215 nm, the lateral dimensions are approximately 2.9 μm (length) and 2.6 μm (width). Dashed rectangles are used to highlight the high-order modes with small PhPs periodicity, showing interference fringes near the crystal edge.

Collectively, ab initio dielectric tensor calculations and wavelength-dependent focusing unequivocally demonstrate the hyperbolic polaritonic properties of our system. Following the reviewer's pertinent observation, we have now added the above discussion on this topic in the revised manuscript (page 14, line 271) and included Figure **R1** as Figure **S10**.

Comment 2: The authors credit the impressive 33% anisotropy of AMO polaritons to in-plane biaxial strain which, if true, suggests tunability of the anisotropy. I do not see convincing evidence that polariton anisotropy owes to biaxial strain. Were this so, I might expect crystals e.g. in Fig. 1c to showcase variable polariton anisotropy relative to the AMO crystal axes, since these crystals are presumably differentially strained

upon the sapphire substrate. Can the authors convincingly identify any variation in polariton anisotropy that could be associated to "tunable" strain? Otherwise, I find it more plausible that optical anisotropy in oscillator strength of the Mo-O stretch mode, allowed by the crystal symmetry, could induce observed polariton anisotropy. Such anisotropy in Raman-active modes is already showcased in Fib. 3b, which credits this scenario. Like issue 1), this controversy could be settled with a measurement, prediction, or at least credible reference to the anisotropic optical constants of AMO. Alternatively, if authors can transfer an AMO crystal from sapphire onto a substrate where strain is mitigated, does the anisotropy change or vanish?

Our response: We thank the reviewer for this insightful comment. This goes to the heart of understanding the physical origin of the remarkable anisotropy observed in our AMO flakes.

The reviewer raises a valid alternative hypothesis that the observed polariton anisotropy could arise from anisotropy in the oscillator strength of the Mo-O stretch mode, as suggested by the polarization-dependent Raman data in Figure **3b**. However, the two orthogonal edges of our square-like flakes are crystallographically equivalent, i.e., both aligned along the diagonals of the *ab*-plane. As shown in Figure **R3**, the atomic arrangement in Figure **R3b** is excellent agreement with Figure **R3c**. This correspondence confirms that the two orthogonal edges of the square-like AMO flake are parallel to the (110) plane, and are rotated by 45° relative to the *a*-axis ([100]) and *b*-axis ([010]), respectively. Consequently, combined analysis identifies the flake edges are crystallographically equivalent. If intrinsic anisotropy (*a* vs. *b* lattice parameters) were the sole origin, we would expect no difference in polariton propagation along these two equivalent directions.

In fact, we observe a significant difference point to an external perturbation—namely, strain, which has been evidenced by the previous work (J.D. Bai, et al., *J. Phys. Chem. Lett.* 2025, 16,3689−3697). The lattice mismatch calculation yields nearly equal but opposite strains (-2.87% and +2.86%), which are sufficient to induce the observed level of anisotropy based on literature precedents for strain-induced phonon splitting in oxides [V.A. Volodin & V.A. Sachkov, *Physica B*: Condensed Matter 2021, 614, 4130082021; etc.].

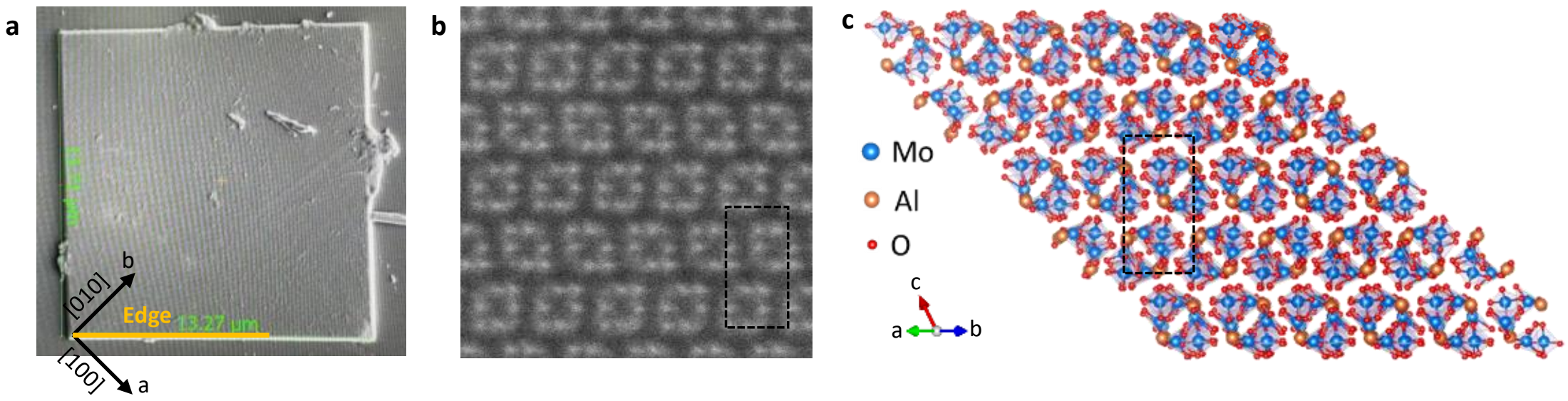

Figure R3. (a) Scanning electron microscope image of a AMO flake. Yellow solid line is used to mark the edge, where the cross-sectional HRTEM was performed. (b) Cross-sectional high-angle annular dark-field STEM image of AMO flake. (c) Atomic structure projected onto the (110) plane.

To directly test whether the polariton anisotropy varies with the crystal orientation (and thus with the orientation-dependent strain state imposed by the sapphire substrate), we performed additional PiFM measurements on a set of adjacent AMO nanoflakes that exhibit different in-plane orientations. Due to limitation of the maximum scan range of our PiFM setup, we selected four neighboring nanoflakes (Figure **R4a**) that are sufficiently close to ensure comparable growth conditions, yet display distinct crystallographic orientations. PiFM images were acquired at a fixed excitation wavenumber of 940 cm^{-1}, as shown in Figure **R4b**, the central rectangular-shaped hot spot rotates systematically with the orientation of each nanoflake. For four adjacent AMO nanoflakes, the aspect ratios of the central

rectangular hot spots extracted from PiFM images (E_1/E_2, ranging from 1.49 to 1.58) are systematically larger than the corresponding geometric aspect ratios measured from AFM topography (L_1/L_2, ranging from 1.06 to 1.27). This discrepancy indicates that the observed shape anisotropy of the hot spot cannot be solely attributed to the flake geometry. Instead, it reflects an additional contribution from strain-induced in-plane dielectric anisotropy, which locally modifies the polariton focusing behavior.

Crucially, because all four flakes are located within a small area (≤ 20 μm), they experience the same macroscopic growth conditions but differ in their epitaxial strain states due to their different in-plane rotations relative to the sapphire lattice. The fact that the polariton anisotropy (as visualized by the hot-spot orientation) varies from flake to flake, and does so in a way that follows the flake's edge orientation, provides strong experimental evidence that the observed anisotropy is intimately linked to the orientation-dependent biaxial strain imposed by the substrate.

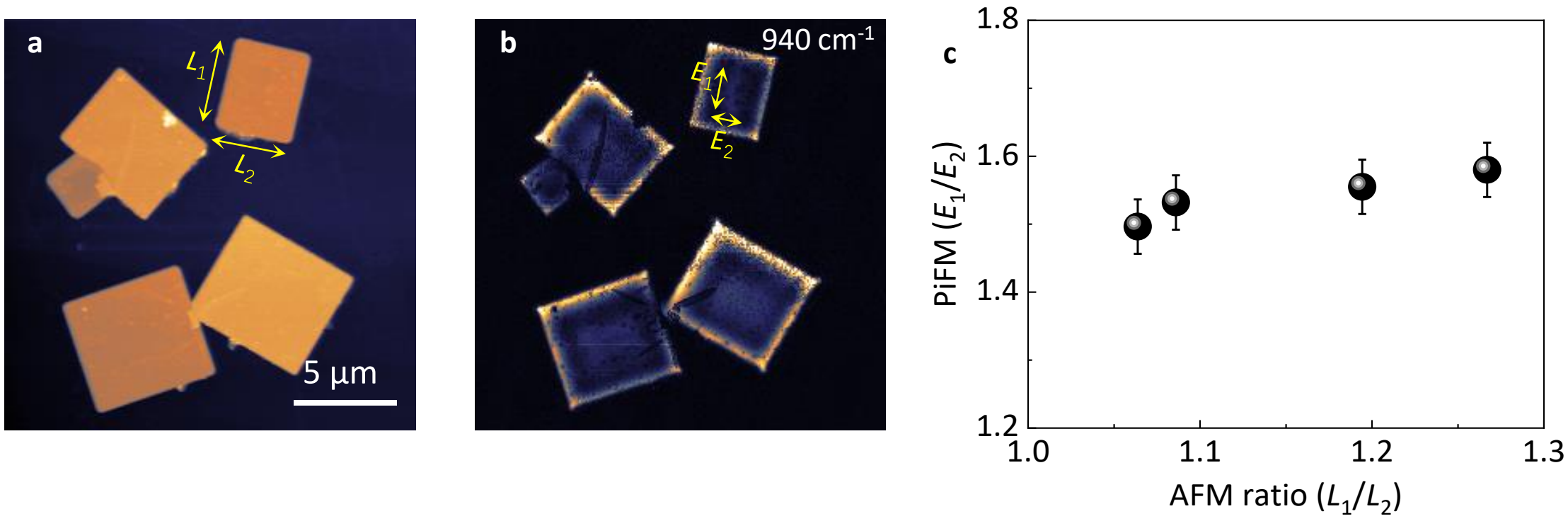

Figure R4. (a) Atomic force microscopy (AFM) of AMO flakes. (b) The corresponding PiFM image at 940 cm^{-1}. (c) Aspect ratios of the central rectangular hot spots extracted from PiFM images (E_1/E_2) vs the corresponding geometric aspect ratios (L_1/L_2), error bars denote standard deviations.

The reviewer's suggestion to transfer an AMO flake to a strain-relieving substrate (e.g., a holey grid or a low-adhesion substrate) is scientifically ideal. We have indeed

attempted such experiments, but encountered two major challenges. (1) Mechanical fragility: Attempts to mechanically lift the flakes using PDMS stamps or adhesive tape resulted in fracture and fragmentation, especially given their large lateral dimensions (tens of micrometers). In most cases, only small triangular fragments (< 5 µm in lateral size) could be transferred, as shown in Figure **R5a,** which are insufficient for PiFM measurements of polariton propagation (which require flakes with dimensions of at least 10–20 µm to clearly resolve fringe patterns). (2) Strong interfacial bonding: AMO grows via an interfacial reaction between MoO_3 and the Al_2O_3 substrate. This chemical reaction likely confirms a robust interfacial bonding, rather than the weak van der Waals interactions typical of exfoliable materials. In addition, the previous work in Figure **R5b** also claimed ionic bonding between the AMO flakes and sapphire substrate.

Nevertheless, we fully agree that such experiments would provide definitive evidence. We have therefore added a statement in the Outlook or Future Directions section (page 14, lines 257-261) acknowledging this as an important goal for future work: "*We note that the potential role of strain in modulating the edge-launched polariton modes observed here remains qualitative. A systematic, quantitative analysis of strain effects would require controlled strain experiments combined with direct strain measurements, which we identify as an important direction for future investigation*".

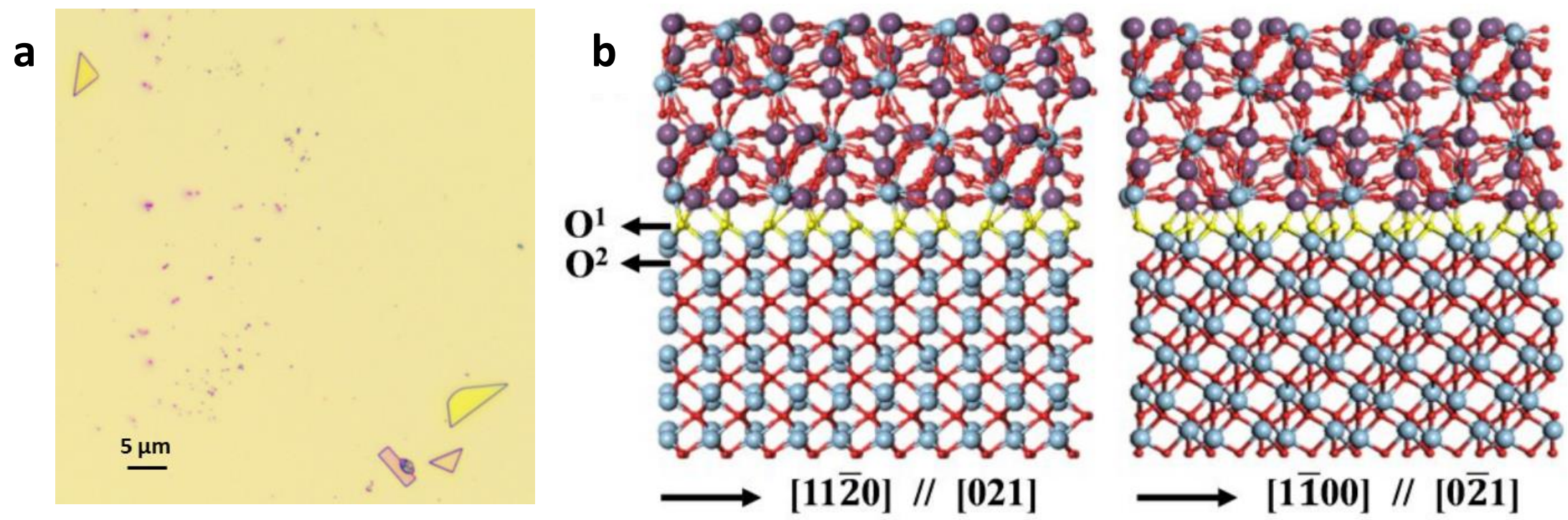

Figure R5. (a) Optical microscopy image of AMO flakes transferred on Au coated SiO2/Si substrate. (b) Side view of AMO along sapphire $[11\bar{2}0]$ and $[1\bar{1}00]$

directions from Figure S10 in reference J.D. Bai, et al., *J. Phys. Chem. Lett.* **2025**, 16,3689−3697.

Comment 3: Table 1 is interesting but problematic in several ways that must be addressed. As mentioned, “hyperbolic type” for AMO is unsubstantiated. “Spectral tunability” for each material under comparison is indicated as “high”; unless a quantitative metric can be supplied, this descriptor should be removed. Certain details of the table are either confusing or incorrect. For instance, hBN is indicated to enjoy spectral tunability through “electrostatic doping”. The ionic solid is an insulator; what electrostatic tuning is referenced? Broadly, a reference should be added for each quoted tuning mechanism.

Our response: We fully concur with the reviewer’s assessment. This is indeed a crucial aspect that deserves more clarifications and discussions. According to the reviewer’s constructive suggestions, we have removed the qualitative description of the spectral tunability (high).

Just as the reviewer stated, it is inherently challenging to modify the charge carrier concentration of hexagonal boron nitride (h-BN) directly through electrostatic doping (i.e., via the application of a gate voltage), due to its wide-bandgap. Almost all current research concerning the "*tuning of h-BN phonon polaritons by electrostatic doping*" is fundamentally based on the mechanism of near-field coupling between graphene plasmons and h-BN phonon polaritons in the stacking Graphene/h-BN heterostructures (*Nature Nanotech.* **2015**, 10, 682-686). In this approach, electrostatic doping (gate voltage) is utilized to modulate the Fermi level of graphene (arxiv.org/pdf/1704.08980), thereby controlling its plasmonic response and indirectly influencing the behavior of the coupled h-BN phonon polaritons. In addition, α-$RuCl_3$ as a 2D electron acceptor, has been used to induce emergent nano-optical behavior

in h-BN arising from interlayer charge polarization (*Nano Lett.* **2023**, 23, 8426–8435), i.e., the distinct work function between α-$RuCl_3$ and h-BN layers induces the charge transfer at the interface, providing an emergent nanoscale electrostatic control strategy.

Although we believe our original description "*electrostatic doping*" was correct, we now realize that the presentation is confusing. We have now replaced it with "*interfacial near-field coupling*" to avoid any potential misunderstanding. We also agree that each tuning mechanism should be properly supported by relevant references. In the revised manuscript, we have now added the appropriate citations for each mentioned tuning mechanism (Table 1).

Comment 4: The manuscript should be augmented to present more technical detail regarding measurement methods used. Nature Communications articles conventionally include a "Methods" section and this should be the case for the present submission in order to explain, for example, the following: How were the X-ray diffraction results presented in Fig. 1f acquired? Are these area-averaged measurements from a sapphire substrate with sparse coverage of AMO? What diffractometer was used? What PiFM instrument and laser were used to acquire the nano-imaging results shown throughout the article? What "signal", explicitly, is imaged in e.g. Fig. 4b? I can infer that these details are provided in Ref. 27, but a similarly detailed explanation of PiFM methods should be provided in the present manuscript. More detail should be supplied to explain how the "nano-infrared absorption" spectrum of Figure S4 was acquired. Is this truly an absorption spectrum, or a PiFM spectrum? Discussion of PiFM methodology should include an explanation of normalization of spectra, if applicable.

Our response: We thank the reviewer for these valuable suggestions to improve the clarity and completeness of our manuscript. We have now substantially augmented the "*Methods section*" to include all the requested technical details. The specific changes are outlined below in a point-by-point manner.

Comment 4-1: The manuscript should be augmented to present more technical detail regarding measurement methods used... How were the X-ray diffraction results presented in Fig. 1f acquired? Are these area-averaged measurements from a sapphire substrate with sparse coverage of AMO? What diffractometer was used?"

Our Response: We have now added a detailed description of the XRD measurements in the Methods section, i.e., the XRD pattern in Fig. 1f was acquired using a SmartLab SE diffractometer (Rigaku, Japan) with Cu K_α radiation (λ = 1.5406 Å).

Regarding the nature of the measurement, we confirm that these are area-averaged measurements. The beam footprint covered a region containing both the sapphire substrate and the sparsely distributed AMO material. This has been clarified in the revised manuscript, page 6, line 107, "*The X-ray diffraction (XRD) pattern in Figure 1f represents an area-averaged signal from the sample, including both the sapphire substrate and the sparse AMO coverage*".

Comment 4-2: What PiFM instrument and laser were used to acquire the nano-imaging results shown throughout the article? What "signal", explicitly, is imaged in e.g. Fig. 4b?

Our Response: We appreciate for pointing this out that further clarification on the PiFM setup and the imaging signal are required. We have now explicitly stated the instrument model and laser source in the Methods section. The PiFM measurements were performed using a VistaScope microscope (Molecular Vista, USA), excited by a pulsed quantum cascade laser (Block Engineering, USA) tunable from 780 to 1950 cm^{-1}.

Explicitly, the signal displayed in Fig. 4b is the photo-induced force (PiFM) signal. In the measurement setup, the AFM feedback laser is directed to the cantilever and reflected to a position-sensitive detector that records the superposition of topographic and PiFM signals. The PiFM signal, which is usually described by a dipole–dipole interaction force model between an AFM tip and the sample, carries the local spatial modulation information of the electric field induced by the excited polaritons on the sample surface. Thus, the optical near-field images of phonon-polaritons interference can be built without detecting any light through this technique.

Comment 4-3: More detail should be supplied to explain how the "nano-infrared absorption" spectrum of Figure S4 was acquired. Is this truly an absorption spectrum, or a PiFM spectrum? Discussion of PiFM methodology should include an explanation of normalization of spectra, if applicable.

Our Response: We thank the reviewer for pointing out this ambiguity regarding the terminology used in Figure S4. The spectrum shown in Figure S4 is indeed a PiFM spectrum. The PiFM spectrum reported herein is physically consistent with a nano-infrared (nano-IR) absorption spectrum. Both techniques probe the intrinsic IR-active vibrational modes of chemical bonds, which are determined solely by the molecular structure and chemical environment, rather than the detection scheme. Therefore, for the purpose of chemical identification, vibrational mode assignment, and nanoscale compositional characterization, the PiFM spectrum is consistent with infrared spectrum. (J. Phys. Chem. C **2018**, 122, 41, 23808–23813; Mater. Chem. Front., **2022**, 6, 1552-1573; Sci. Adv.**2016**, 2, e1501571; Proc. Natl. Acad. Sci. U.S.A. **2019**, 116, 26359-26366). The figure caption has been updated in the revised Figure **S6** to clarify that these are PiFM spectrum, reflecting the local infrared response at specific wavenumbers.

We have expanded the PiFM methodology section to include the spectral

acquisition details. The spectra were acquired by recording the PiFM signal while tuning the laser wavenumber. To account for the laser power variation across the tuning range, all spectra were normalized by the laser power profile, which was measured simultaneously during the scan. This normalization procedure ensures that the resulting spectra represent the material's intrinsic optical response. This detail has now been added to the Methods section.

We believe these additions have significantly strengthened the manuscript and provided the necessary transparency regarding our experimental methods.

Comment 5: The authors quote the maximum propagation length for polaritons in AMO. Since this figure clearly depends on the polariton wavelength, which itself varies with energy and also (presumably) AMO thickness, it is a less useful comparative metric than the polariton quality factor. Can the authors include this metric in comparison to the values for "alternative" materials in their Table 1?

Our response: We thank the reviewer for this insightful suggestion. We agree that the polariton quality factor ($Q = L_p / \lambda$) serves as a more intrinsic and comparable metric across different material systems, as it normalizes the propagation length by the wavelength.

Following this recommendation, we have calculated the quality factor of the AMO polaritons observed in our study. Based on the extracted propagation lengths and corresponding wavelengths, the quality factor is approximately Q = [4.86$\pm$0.3]. Table 1 has been updated to include a new column for the quality factor. We have also added comparative Q values for the alternative materials discussed (e.g., *h*-BN, α-MoO_3) based on literature reports. In the revised manuscript, page 13, line 244, we wrote: " *As summarized in Table S1, the quality factor (Q) [W.Y. Chien, et al. J. Appl.*

Phys. ***2009****, 106, 044310] along the two orthogonal edges are respectively 4.48 (edge1) and 4.86 (edge2) via normalizing the propagation length (L_p) in Figure S7b by the wavelength (λ), both falling within the lower range of values reported for quaternary oxides and Li/Na-V_2O_5".*

We believe this addition makes the comparison presented in Table 1 more meaningful and rigorous.

Comment 6: Polaritons in several AMO crystals of different thickness can be identified in the images. How does the observed polariton wavelength depend ("tunably") on crystal thickness?

Our Response: We thank the reviewer for raising this interesting point. To address it, we have performed additional PiFM measurements on AMO crystals with varying thicknesses, ranging from 130 nm to 500 nm. Crucially, all flakes selected for this thickness-dependent study have lateral dimensions larger than 30 µm × 30 µm. This ensures that the observed fringe patterns originate primarily from propagating polaritons rather than from focusing effects (e.g., ring shrinkage or hot-spot formation), which become dominant in smaller flakes. The large flake size allows us to extract reliable propagation lengths and polariton wavelengths over extended distances, free from geometric confinement artifacts.

As shown in Figure **R6**, the polariton propagation behavior changes systematically with flake thickness:

- Thinner flakes (e.g., ~132 nm) exhibit stronger in-plane anisotropy (anisotropy ratio ≈ 1.53) and shorter propagation lengths, consistent with a higher degree of retained epitaxial strain.
- Thicker flakes (e.g., ~470 nm) show reduced anisotropy (ratio ≈ 1.33) and slightly

longer propagation lengths, indicating progressive strain relaxation.

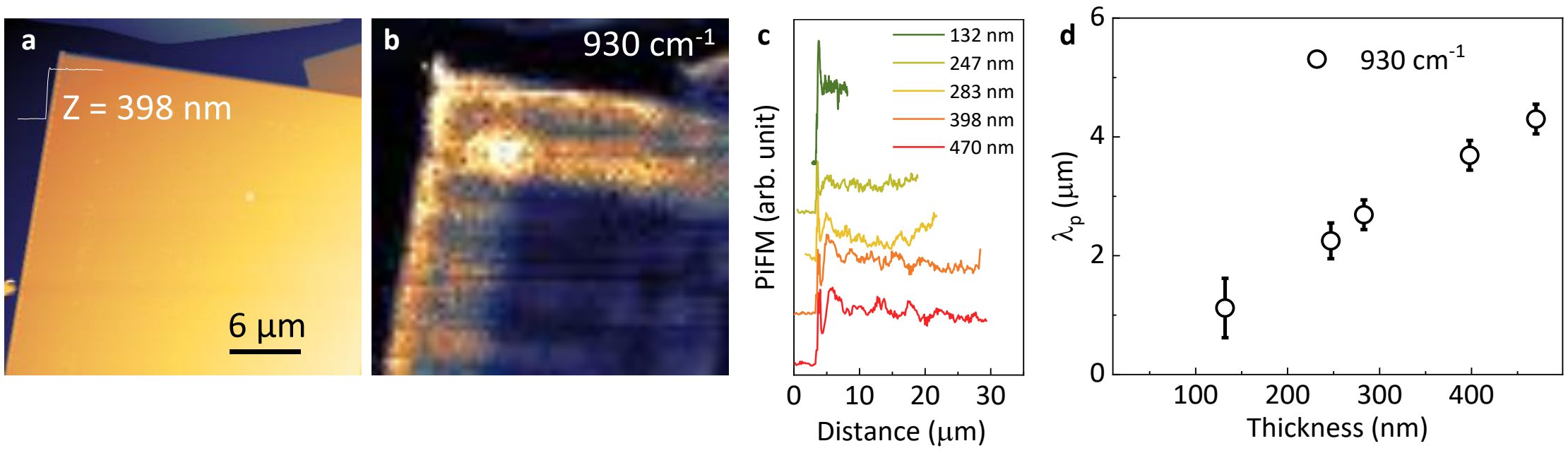

Figure R6. (a) A typical AMO flake with a thickness t = 398 nm. (b) The corresponding PiFM image at 930 cm^{-1}. (c) Phonon polariton line profiles for AMO crystal with different thickness. (d) Thickness-dependent wavelengths of phonon polaritons (λ_p) probed at 930 cm^{-1}.

These trends are in good agreement with the lattice-mismatch-derived biaxial strain (–2.87 % and +2.86 % along orthogonal directions), which is expected to relax as thickness increases. The detailed results, including extracted polariton wavelengths and anisotropy ratios, have been added to Table **R1**.

Table **R1.**

Flake thickness (nm)	Polaritonic wavelength (μm)	Anisotropic ration
470	4.3	1.33
398	3.69	1.38
283	2.61	1.45
247	2.25	1.49
132	1.12	1.53

We have added a new supplementary figure (Figure **S9**) showing representative

line profiles and the extracted wavelength as a function of thickness. The following sentence has been added to the revised manuscript, page 14, line 254. "*we further observed a tunable polariton response by examining crystals with different thicknesses: the fringe period (i.e., polariton wavelength) scales with the flake thickness in Figure* ***S9****, consistent with the thickness-dependent dispersion expected for strongly confined polaritons [Science* ***2014****, 343, 1125.].*"

We thank the reviewer for encouraging us to explore this parameter space, which has added valuable insight into the tunability of AMO polaritons.

Comment 7: The concept of “formation energy above hull” (Fig. 1g) will be unfamiliar to most of the general readership of this journal and should be given more explanation. Furthermore, how were the data points in Fig. 1g predicted? If these were obtained by novel electronic structure calculations, then calculation details should be provided. If values are taken from literature, references should appear in the Figure and/or its caption.

Our Response: We thank the reviewer for raising this point. We realize that the term "*formation energy above hull*" (E_{hull}) may not be immediately familiar to a broad readership. In essence, the energy above hull is a descriptor of thermodynamic stability derived from first-principles calculations. It represents the energy difference between a given structure and the most stable combination of competing phases at the same chemical composition (the convex hull). A value of E_{hull} = 0 meV/atom indicates a stable (ground state) phase that is thermodynamically stable against decomposition. Conversely, a positive value indicates a metastable phase; the smaller the value, the more likely the phase is to be experimentally synthesizable. For example, a material with E_{hull} < 70 meV/atom is often considered potentially synthesizable [W.H.

Sun, et al. Sci. Adv. **2016**, 2, e1600225].

Regarding the origin of the data points in Figure 1g, we confirm that these values were not obtained from our own electronic structure calculations, but rather were retrieved from the Materials Project database (https://materialsproject.org), a well-established, publicly accessible repository of computed materials properties based on density functional theory (DFT). To ensure proper attribution and transparency, we have added a brief explanatory sentence in the revised manuscript, the Figure 1g caption now explicitly states that "*a lower E_{hull} indicates higher thermodynamic stability. All data were retrieved from the Materials Project database (Nature Materials 2025, **24**, 1522)*". The sentence: "*The specific material identifiers used are: [AMO: mp-19521], [MoO_2: mp-559140], [MoO_3: mp-20589], [MoO_4: mp-1180203], [Mo_4O_{11}: mp-542610], and [Al_2O_3: mp-1143]*", has also been added in the Methods section.

Comment 8: The discussion of modal evolution with frequency in the AMO crystal shown in Fig. 5 is interesting, but what is the main conclusion of this analysis? If the answer is the remarkable confinement (Fig. 5d), can the authors rationalize its scale? Can the authors provide a simple simulation to rationalize these images? And further, can the authors hypothesize why polariton images show significant asymmetry within these apparently symmetric crystals?

Our Response: We thank the reviewer for the positive feedback on Fig. 5 and for raising these important points that have helped us strengthen the manuscript.

As the reviewer correctly inferred, the main conclusion of this analysis is the remarkable electromagnetic field confinement achieved in our AMO nanoislands. To rationalize the scale of the confinement observed in Fig. 5d, we have quantified the polariton compression ratio, defined as λ_0/λ_p, where λ_0 is the free-space excitation

wavelength and λ_p is the polariton wavelength extracted from the fringe period. The extracted compression ratio for the focused spot is approximately 17. This strong confinement underscores AMO's potential for sub-diffractional photonic devices such as superlenses or hyperlenses.

We sincerely appreciate the reviewer's valuable suggestion of electromagnetic simulations to rationalize the experimental images, and we have carefully evaluated the feasibility of this work within the scope of the current manuscript. The core innovation of this study is the development of a novel interfacial epitaxy strategy to synthesize high-quality single-crystalline AMO flakes, which supports phonon-polaritons. The polaritonic modes observed in our experiments are synergistically modulated by multiple complex factors in the real epitaxial system, including biaxial epitaxial strain, substrate interface coupling, three-dimensional size effects of the nanoflakes. A simplified simulation that ignores these factors cannot accurately reproduce the experimental results, and may even introduce misleading conclusions due to over-simplified assumptions. On the other hand, a full-factor simulation that fully considers all the above effects involves extremely complex multi-physics calculations, which goes far beyond the core scope of this work. More importantly, we have systematically elucidated the physical mechanism of the observed mode evolution through the combination of ab initio dielectric tensor calculations, quantitative dispersion analysis, and PiFM experimental characterization, which forms a complete evidence chain and fully supports our core conclusions. Therefore, we plan to carry out systematic full-wave electromagnetic simulations and in-depth theoretical analysis of the polaritonic mode engineering in our follow-up work, which will focus on the nanophotonic device applications of AMO. We have therefore clearly indicated in the revised manuscript (page 14, line 259): "*a systematic, quantitative analysis of strain effects would require controlled strain experiments combined and systematic*

numerical simulations, which we identify as important directions for future investigation".

Regarding the asymmetry in apparently symmetric crystals (Figure **R4** & Figure **R6**): The observed asymmetry originates from biaxial epitaxial strain imposed by the sapphire substrate. Although the AMO nanoflakes appear geometrically symmetric (square-like), the lattice mismatch between AMO and *c*-sapphire leads to unequal in-plane strains: compressive (-2.87%) along one orthogonal direction and tensile (+2.86%) along the other. This strain state breaks the in-plane symmetry, resulting in anisotropic dielectric permittivity and, consequently, different polariton propagation behaviors on adjacent edges.

Once again, we sincerely thank the reviewer for these constructive comments, which have helped us significantly improve the clarity and rigor of the manuscript. We hope that our detailed response and the revised manuscript have fully addressed all the questions raised by the reviewer.

Comment 9: EDX mapping in Fig. 2b appears to show an excess of oxygen at crystal edges; can the authors assess whether this is a genuine feature of these crystals, and whether it could have any impact on polaritons "launched" from edges?

Our response: We thank the reviewer for this careful observation. The apparent oxygen enrichment at the crystal edges in the original EDX mapping (Fig. 2b) is indeed an artifact caused by sample charging, rather than a genuine compositional feature of the AMO flakes. To eliminate charging effects, we attempted to transfer AMO flakes onto a conductive gold thin film (on a Si substrate) for EDX analysis. However, the transfer process proved challenging: the AMO flakes grown on sapphire adhere strongly and are brittle, making them prone to fracture during mechanical exfoliation. As a result, most flakes broke during transfer—in one case, a square-shaped flake fractured into a triangular fragment (Figure **R7**). Nevertheless, this transferred flake allowed us to acquire EDX maps under charging-free conditions. The oxygen map shows uniform distribution across the flake, with no edge enrichment. This confirms that the previously observed edge brightening was an artifact of charging and does not reflect the actual composition of AMO, so its impact on polaritons cannot be evaluated from current data.

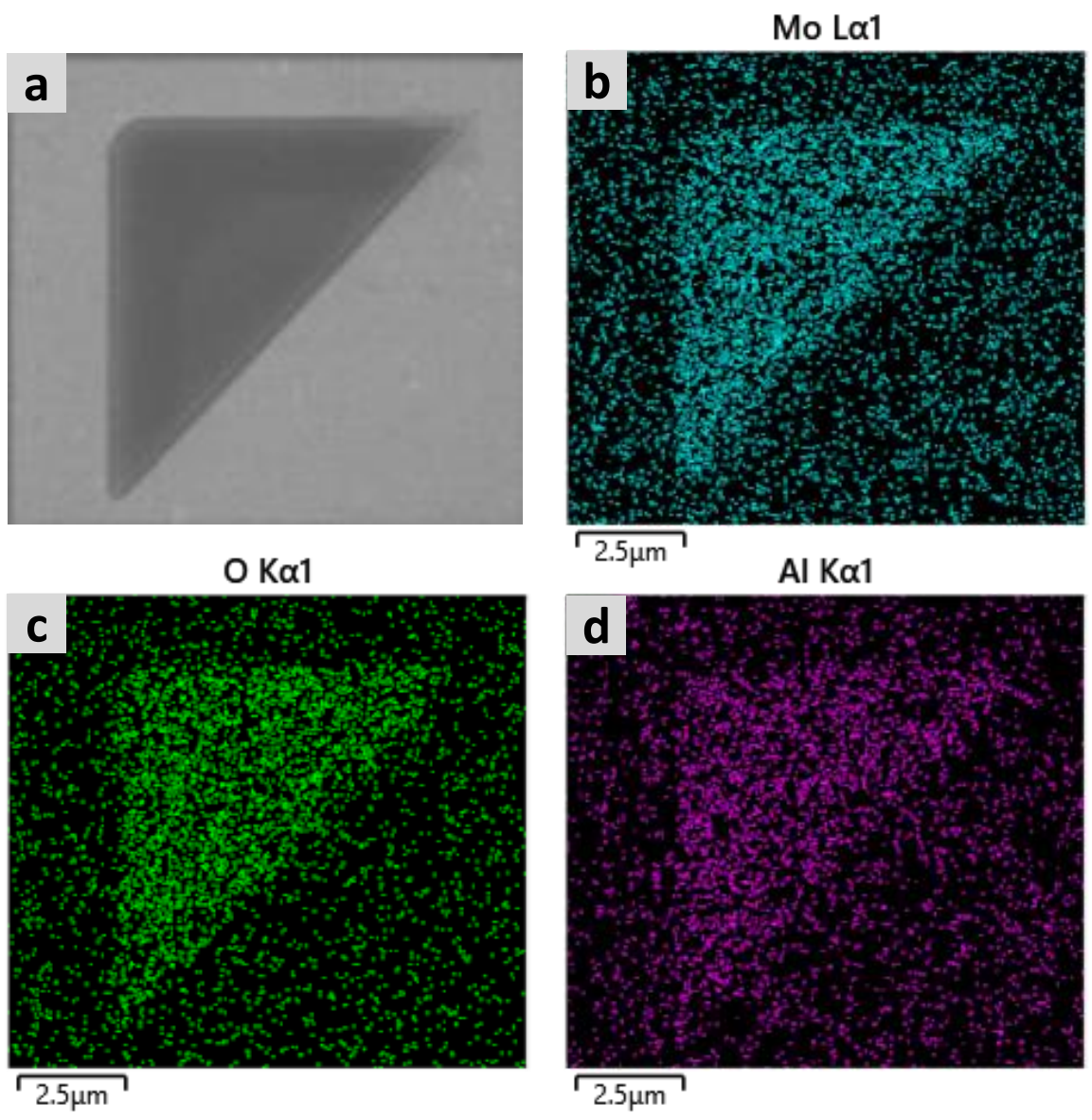

Figure R7. (a) SEM image of a transferred AMO flake. EDX mapping of molybdenum (b), oxygen (c) and aluminum (d) elements was performed after transferring the AMO flake onto a conductive gold thin film to eliminate charging artifacts.

In the revised manuscript, page 8, line 136, we wrote “*The edge brightening observed in the oxygen map is attributed to sample charging. This was confirmed by transferring an AMO flake onto a conductive gold substrate, where EDX mapping (Figure* ***S5****) revealed uniform elemental distribution, demonstrating compositional homogeneity.*”

The following minor issues were also identified:

We thank the reviewer for their careful reading of our manuscript and for identifying these minor issues, which we have now corrected.

Comment 10: Line [175] of the manuscript asserts “the resulting AMO structure … is schematically illustrated.” Where?

Our Response: We apologize for the lack of clarity. The schematic illustration referred to in line 175 is Figure 1. We have now revised the sentence to read: "*The resulting AMO structure is schematically illustrated in Figure 1a.*"

Comment 11: Line [252] of the manuscript references Fig. 5b, then puzzlingly refers to an irrelevant panel in Fig. 4b.

Our response: We thank the reviewer for pointing out this oversight. The reference to Fig. 4b at line 252 was a typo and has been removed from the revised manuscript to ensure consistency with the discussion of Fig. 5b.

Comment 12: This polaritonic medium and its accessible synthesis method will be of broad interest to the photonics community. But major claims of the manuscript must be substantiated with more evidence before it is acceptable for publication.

Our Response: We sincerely thank the reviewer for their positive assessment of our work and for acknowledging that our polaritonic medium and its synthesis method will be of broad interest to the photonics community.

We also fully agree with the reviewer that the major claims of the manuscript must be rigorously substantiated. In response to this important requirement, we have carefully addressed all the specific comments raised throughout the review process.

We believe that these revisions, together with the point-by-point corrections of the minor issues, have substantially strengthened the evidence supporting our major conclusions. We hope that the manuscript is now suitable for publication in Nature Communications.

For Reviewer #2:

Reviewer #2: In this manuscript, the authors present a novel interfacial epitaxy method for synthesizing 2D single-crystalline $Al_2(MoO_4)_3$ (AMO). This strategy consists in putting a molybdenum foil in between two sapphire wafers and heating them up in air atmosphere. Once synthesized, the authors thoroughly characterize the AMO single crystals on sapphire by Raman spectroscopy, X-ray diffraction, and scanning transmission electron microscopy (STEM). The authors then study AMO's mid-IR near-field optical response by performing photo-induced force microscopy (PiFM) scans to map the phonon-polariton (PhPs) dispersion in AMO. In my opinion, the manuscript, in its current form, requires major revision. While the synthesis and structural characterization of 2D single-crystalline Al2(MoO4)3 (AMO) are interesting, the work lacks sufficient theoretical, analytical, and comparative support, particularly regarding the near-field polaritonic experiments and the positioning of AMO within the broader family of mid-infrared polaritonic materials.

Our Response: We thank the reviewer for the positive acknowledgment of our synthesis and structural characterization work, and for the constructive overarching assessment. We agree with the reviewer that this manuscript could be strengthened with additional theoretical, analytical, and comparative support. In the revised manuscript, we have comprehensively addressed these aspects, accordingly. Below we provide a point-by-point response.

Comment 1: The authors must include a detailed Methods section in which all experimental procedures are described comprehensively. The experimental details of the PiFM near-field measurements should be explained thoroughly to ensure reproducibility.

Our Response: We thank the reviewer for this important reminder. In response, we have significantly expanded and detailed the Methods section in the revised manuscript. Specifically, we have now included:

- *Sample preparation*. The $Al_2(MoO_4)_3$ nanoflakes were synthesized by a space-confined method with a Mo foil (thickness: 0.05 mm, lateral dimensions: 5 mm × 5 mm, purity: 99.99%) sandwiched between two sapphire substrates. The sandwich structure was then heated in air to 700 °C at 5 °C/min, maintained for several hours, and then furnace-cooled to room temperature, where the oxidized Mo foil acted as the MoO_3 precursors and reacted with the Al_2O_3 substrate, driving the interfacial nucleation of $Al_2(MoO_4)_3$ nanoflakes.

- *Characterizations of the crystal and topographic structures.*

1. *X-ray diffraction*. X-ray diffraction (XRD) patterns were obtained using a SmartLab SE diffractometer (Rigaku, Japan) with Cu K_α radiation (λ = 1.5406 Å), data were collected in the 2 θ range from 10° to 80°.

2. *STEM measurements*. The TEM samples were prepared using focused ion beam (FIB) (ThermoFisher Helios 5UX). Lamellae were prepared along the edge of the $Al_2(MoO_4)_3$ nanoflakes. High-resolution HAADF-STEM images was acquired using a TherThermoFisher Spectra 300.

3. *AFM measurements*. The topography of the $Al_2(MoO_4)_3$ nanoflakes were imaged in contact-mode by a Cypher-S AFM with commercially available PtIr-coated Si tip.

- *Photo-induced force microscopy (PiFM)*. Near-field optical images were acquired using a commercial atomic force microscope (VistaScope, Molecular Vista, USA) equipped with a PiFM module. All PiFM measurements were performed using a pulsed, widely tunable quantum cascade laser (Block Engineering, USA) as the excitation source, covering a seamless narrowband wavenumber range of 780–1950 cm^{-1}. The laser features a spectral linewidth of 2 cm^{-1} and a resolution of 0.5 cm^{-1}.

The experiments were operated in sideband excitation mode, where the laser frequency was modulated at $f_m = f_1 - f_0$. Here, f_0 represents the first mechanical eigenmode resonance of the cantilever used for PiF signal detection, while f_1 corresponds to the second eigenmode resonance employed for the simultaneous acquisition of AFM topography.

Gold-coated silicon probes (HQ:NSC15/CR-AU, MikroMasch Inc.) with a resonant frequency of approximately 300 kHz were used for all measurements.

We believe these additions now provide the necessary level of detail to ensure full reproducibility of our experiments. The Methods section can be found on pages [19–21] of the revised manuscript.

Comment 2: In Figure 1 panel c, there is a missing “Z” or similar to denote the magnitude that is plotted in this panel (topography).

Our response: We thank the reviewer for pointing out this oversight. The label “Z” has now been added to Figure 1c to clearly denote the topographic magnitude.

Comments 3: The FFT resolution of the experimentally extracted polariton dispersion in Fig. 4 is notably low, which limits the accuracy of the reported wavevector–frequency relationship. The authors should improve the FFT resolution and clarify how this limitation affects the extracted polaritonic parameters.

Our response: We fully agree that high-resolution wavevector–frequency data are essential for accurate determination of polaritonic parameters. The limited FFT resolution in Fig. 4 originates from the maximum sampling pixel position of the photo-induced force (PiF) spectra. This finite pixel number, together with the typical scan size (~20–40 μm), restricts the number of real-space sampling points along a

given line profile, thereby limiting the intrinsic wavevector resolution of the FFT ($\Delta q \approx 2\pi/L$, where L is the spatial length of the line profile).

Within these hardware constraints, we have optimized the data processing as follows: (1) Optimized the FFT algorithm: We have applied zero-padding to the real-space line profiles before FFT, which interpolates the frequency domain and provides a smoother representation of the dispersion, although it does not inherently increase the true resolution. (2) Averaged multiple line profiles: To improve the signal-to-noise ratio, we performed repeated line-scan measurements on the same region and averaged the acquired profiles. We have included the updated, higher-quality FFT spectra in the revised Figure 4.

As to the possible effect on the extracted polaritonic parameters due to this limitation, we here try to perform the analysis. We think the group velocity is less affected by the finite resolution, as peak positions can be determined with sub-pixel accuracy through Gaussian fitting of the FFT peaks. However, our reported Q values ($Q = Re(q)/2Im(q)$) should be considered as lower-bound estimates, as peak broadening from limited resolution can artificially increase Im(q).

Comment 4: The authors employ phrases such as "critically needed," "fills a critical gap," and "highly promising candidate" multiple times in the manuscript. The authors should soften them to maintain a balanced and objective tone consistent with the level of evidence provided.

Our Response: We have carefully reviewed the manuscript and softened or rephrased the overly assertive statements throughout the text. Specific modifications include:

The original sentence "*single-crystalline $A_2(MoO_4)_3$ is therefore <u>critically needed</u> to unlock fundamental studies of anisotropic polaritonic phenomena and to enable*

prospective photonic and optoelectronic applications." now reads: "*The synthesis of high-quality, single-crystalline $A_2(MoO_4)_3$ provides an additional material platform for exploring anisotropic polaritonic phenomena and prospective optoelectronic applications*" in the revised manuscript, page 4, line 53.

The original sentence "*This distinct spectral band thereby fills a critical gap in the mid-infrared polaritonic landscape, extending the accessible frequency window for nanophotonic and optoelectronic applications.*" now reads " *The polaritonic modes in AMO flakes emerge within a spectral window of 900–970 cm^{-1}, which overlaps with the Reststrahlen band of α-MoO_3 while extending to slightly lower wavenumbers. This spectral region therefore complements the existing mid-infrared polaritonic landscape by offering an additional material platform (Table 1).*" in the revised manuscript, page 17, line 324.

The original sentence "*Beyond establishing a general synthesis platform for a coveted class of materials, this work introduces a highly promising candidate for infrared polaritonics.*" now reads "*Beyond establishing a general synthesis platform for a coveted class of materials, this work introduces a material of interest for infrared polaritonics*" in the revised manuscript, page 18, line 338.

We believe these revisions bring the manuscript's language into better alignment with the standards of cautious and objective scientific writing. We thank the reviewer for helping us improve the clarity and professionalism of our presentation.

Comment 5: The manuscript frequently refers to AMO flakes with thicknesses of 50–500 nm as "two-dimensional (2D)". In the context of nanophotonics and polaritonics, this terminology is typically reserved for ultrathin or quasi-2D materials. The authors should reconsider this terminology (for instance, use single thin layers).

Our response: We thank the reviewer for this terminological clarification. We agree that in the context of nanophotonics and polaritonics, the term "*two-dimensional (2D)*" is conventionally reserved for ultrathin van der Waals materials with atomic-scale thickness (e.g., monolayer or few-layer graphene, hBN, or transition metal dichalcogenides). In response, we have carefully revised the manuscript to replace all instances of "*2D*" with more accurate terminology. Specifically, "*2D AMO flakes*" has been replaced with "*thin AMO flakes*" or "*thin-layer AMO crystals*" throughout the text.

Comment 6: The authors state: "Moreover, the polaritonic modes in AMO flakes are exclusively observed within a narrow frequency window (900–970 cm^{-1}), complementing previously reported PhP propagation bands in van der Waals materials such as h-BN31 an α-MoO332, 33, 34 (Table 1). This distinct spectral band thereby fills a critical gap in the mid-infrared polaritonic landscape, extending the accessible frequency window for nanophotonic and optoelectronic applications.", but the said spectral regime widely overlaps with α-MoO3 Reststrahlen band. The authors must temper this claim and clarify what uniquely distinguishes AMO beyond its specific spectral window.

Our response: We thank the reviewer for this incisive observation. The spectral window of AMO (900-970 cm^{-1}) indeed overlaps with the Reststrahlen band of α-MoO_3, and our original phrasing—particularly the term "*fills a critical gap*"—was inaccurate and overstated. We have revised the manuscript accordingly.

The revised text now reads: " *Moreover, the polaritonic modes in AMO flakes emerge within a spectral window of 900–970 cm^{-1}, which overlaps with the Reststrahlen band of α-MoO_3 while extending to slightly lower wavenumbers. This spectral region therefore complements the existing mid-infrared polaritonic landscape*

(e.g., h-BN and α-MoO_3) by offering an additional material platform (Table 1)", page 17, line 324.

In addition, we have now articulated the following distinctive features of AMO crystal:

- Synthesis accessibility and scalability: Unlike α-MoO_3, which typically requires mechanical exfoliation from bulk crystals, AMO can be synthesized via a solution-based, scalable method that yields high-quality, single-crystalline flakes. This accessibility makes AMO a practically attractive platform for large-scale integration.
- Extended spectral coverage: Although the 900-970 cm^{-1} window overlaps with α-MoO_3, AMO's Reststrahlen band also extends to slightly lower frequencies, offering potentially useful spectral complementarity for applications requiring fine-tuning within this range.

Thus, the novelty of AMO lies not in offering an entirely new frequency band, but in providing a new, accessible, and structurally distinct material platform within a technologically important spectral range. This positions AMO as a complementary addition to the family of mid-infrared polaritonic materials, rather than a replacement or a gap-filler. In the revised manuscript, page 17, line 328, we wrote "*In this context, the successful synthesis of high-quality, single-crystalline AMO—characterized by its distinct crystal symmetry, facile synthetic route, and robust stability—further expands the materials toolkit for nanophotonic and optoelectronic applications*".

We believe these changes present a more accurate and compelling case for the significance of our work.

Comment 7: The authors state: "The synthesis of high-quality, single-crystalline $A_2(MoO_4)_3$ is therefore critically needed to unlock fundamental studies of anisotropic polaritonic phenomena and to enable prospective photonic and optoelectronic

applications.” The claim that the synthesis of single-crystalline AMO is “critically needed” to unlock polaritonic studies is too strong, since in its Restrahlen Band overleap with other mid-IR polaritonic materials. Moreover, the generality of the synthesis approach is not demonstrated beyond AMO. The authors must rewrite this part with a more balanced tone and clearer positioning relative to existing materials.

Our Response: We thank the reviewer for this constructive critique. The synthetic route is primarily reported here, and the claim of “*critically needed*” is indeed too strong. In the revised manuscript, page 4, line 53, we have modified the text to read" *The synthesis of high-quality, single-crystalline $A_2(MoO_4)_3$ provides an additional material platform for exploring anisotropic polaritonic phenomena and prospective optoelectronic applications* ".

Comment 8: There are some relevant references of pioneering polaritonic works in hBN (Science 2014, 343, 1125-1129) and in α-MoO3 (Nature 2018, 562, 557–562) missing.

Our response: We thank the reviewer for pointing out the omission of these two seminal works. They are very relevant references for any manuscript discussing phonon polaritons in van der Waals materials. In response, we have now added both citations to the revised manuscript as reference [33] and [41].

Comment 9: The AMO crystals are compared to benchmark polaritonic materials such as h-BN and α-MoO3 (Table 1). However, these materials are typically obtained as atomically flat, exfoliated flakes. The AMO flakes reported here have thicknesses between 50 and 500 nm and no quantitative surface roughness analysis is provided. A systematic AFM roughness study (RMS roughness over representative areas) is

necessary to assess whether the surface quality is suitable for low-loss nanophotonic applications and to enable a fair comparison with established 2D polaritonic materials.

Our response: We thank the reviewer for this important request. To address this, we have performed a systematic AFM roughness analysis on representative AMO flakes and now include the results in the revised manuscript.

We acquired high-resolution AFM topography images on several AMO flakes. The root-mean-square (RMS) roughness was calculated using standard AFM analysis software (AR 16.10.211). For the flake shown in Figure 1c, we selected multiple regions of 0.35 μm × 0.35 μm, the RMS roughness measured in all these regions is 21 pm (0.021 nm). This value is representative of those obtained across multiple flakes and regions. As shown in Figure **R8**, a typical topographic AFM image demonstrates the ultra-low RMS roughness.

In the revised manuscript (page 6, lines 95), we wrote "*AFM roughness analysis yields a root-mean-square (RMS) roughness of 21 pm, indicating the smooth surface of AMO flakes (Figure **S3**)*". The calculation of RSM is also included in the Methods section.

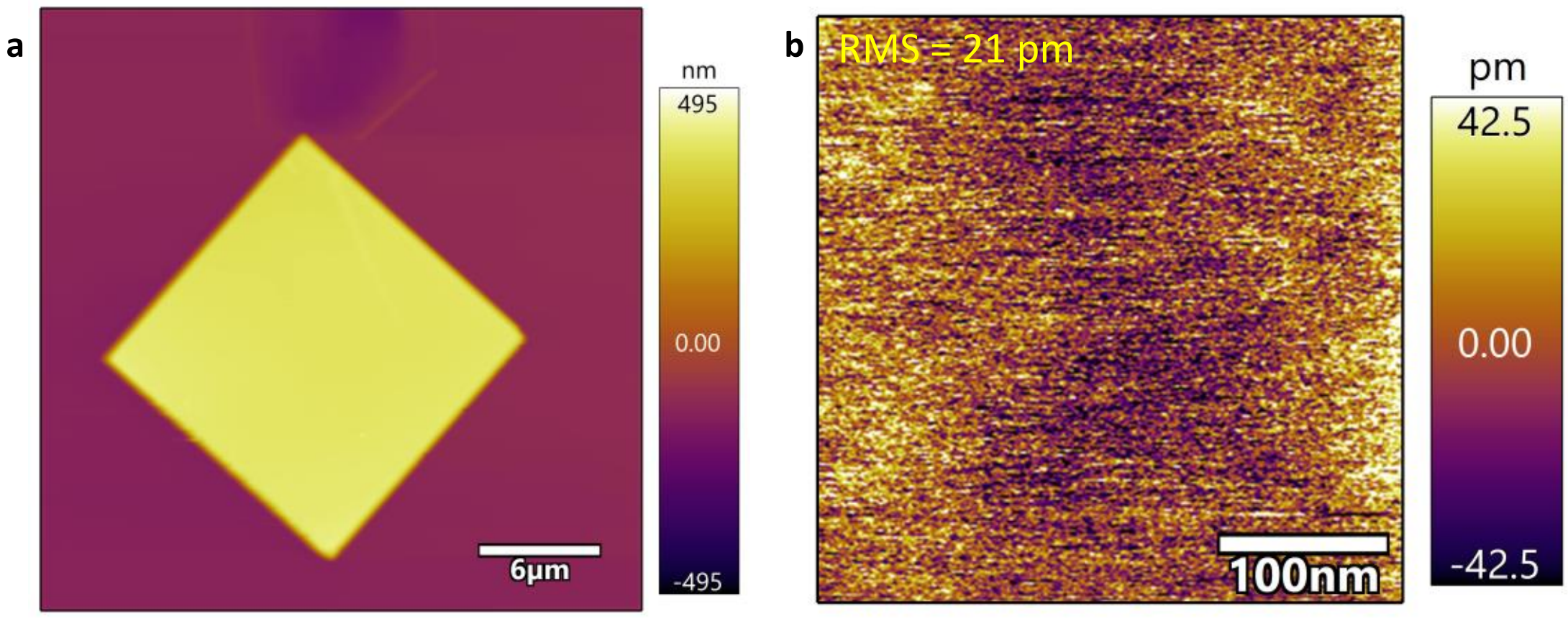

Figure R8. (a) AFM topography image of an AMO flake. (b) Enlarged view of the AMO flake where the RMS is calculated.

Comment 10: Some parameters listed in Table 1 (e.g., propagation length and tunability) are presented without specifying comparable experimental conditions such

as substrate, thickness, or excitation frequency. The authors must clarify these conditions to improve the usefulness of the comparison.

Our Response: We thank the reviewer for this important observation. Since the polariton wavelength varies with its energy and sample thickness, it is a less useful comparative metric than the polariton factor. The polariton quality factor ($Q = L_p / \lambda$) can serve as a more intrinsic and comparable metric across different material systems, as it normalizes the propagation length by the wavelength.

Following this, we have calculated the quality factor for the AMO polaritons observed in our study. Based on the extracted propagation lengths and corresponding wavelengths, the quality factor is approximately Q = [4.86 ± 0.3]. Table 1 has been updated to include a new column for the quality factor. We have now added comparative Q values for the alternative materials discussed (e.g., *h*-BN, α-MoO_3) based on literature reports.

We believe these modifications significantly improve the usefulness and scientific rigor of the comparison presented in Table 1.

Comment 11: The manuscript currently compares AMO only with h-BN and α-MoO_3. Given the rapidly expanding family of mid-IR polaritonic materials, a broader comparison is necessary. The authors should contextualize AMO against other recently reported systems, such as quaternary oxides (Nat. Nanotechnol. 2024, 19, 758–765), Li- and Na-V2O5 (Nat. Commun. 2024, 15, 2696 and Nat. Mater. 2020, 19, 964–968), among others, using consistent polaritonic metrics.

Our response: We thank the reviewer for this insightful suggestion. In response, we have prepared Table **S1** to include the recently reported material systems suggested by the reviewer.

Parameters	$Al_2(MoO_4)_3$	Quaternary oxides	Na/Li-V_2O_5
**Polariton Type**	Hyperbolic	Hyperbolic	Hyperbolic
**Anisotropy**	In-plane	In-plane	In-plane
**Frequency Range**	900-970 cm^{-1}	860-950 cm^{-1}	948-1024 cm^{-1}
**Spectral Tunability**	Epitaxial strain/Lateral sizes	Cation substitution	Na intercalation
**Polariton quality factor**	4.86	6-10 (*Nature Nanotechnology, 2024, 19.6: 758-765.*)	5 (*Nat. Commun*. 2024, 15, 2696 and Nat. Mater. 2020, 19, 964–968)
**Synthesis Methods**	Interfacial epitaxy	Chemical flux growth + exfoliation	Intercalation of Na/Li into V_2O_5 vdW crystals

Comment 12: The authors state "The polaritonic propagation length L_p = 20.9 μm is two times larger than that of α-MoO_3 flakes (**Table1**)". As noted above, meaningful comparisons of polaritonic figures of merit require measurements performed under comparable conditions, for instance when the materials are placed on mid-IR transparent substrates. In addition, the main text manuscript does not clearly specify along which in-plane crystallographic direction this propagation length is extracted, which is particularly important given the anisotropic nature of the system.

Our Response: We thank the reviewer for this important methodological critique. We fully agree that direct comparisons of propagation length (L_p) across different materials and studies can be misleading if experimental conditions (substrate, thickness, excitation frequency) are not carefully matched. In response to this concern—which was also raised by Reviewer #1—we have substantially revised our approach to comparing polaritonic performance. As detailed in our response to Reviewer #1 and previous comments, we have replaced direct L_p comparisons with the more robust metric of quality factor (Q), defined as $Q=L_p/\lambda_p$. This metric normalizes the propagation length by the polariton wavelength, providing a material-intrinsic figure of merit that is less sensitive to experimental conditions such as

excitation frequency and flake geometry [Basov et al., Science 2016; Ma et al., Nature 2018].

Accordingly, we have removed the statement comparing L_p values from the main text. In its place, we now present a comparison based on quality factors in Table 1, where AMO (Q≈4.86) is contextualized alongside hBN (Q≈50–480), α-MoO_3 (Q≈76–200), and other recently reported systems (e.g., LiV_2O_5, quaternary oxides in Table S1).

Given the anisotropic nature of AMO, the direction of polaritonic propagation is indeed critical. In the revised manuscript, we have now explicitly specified that the propagation length and corresponding Q factor were extracted along the edge2 direction of the AMO flake. The relevant sentence in the revised manuscript, page 13, Line 241, now reads: " *line profiles (Figure S7a) yield a polariton period of* $\lambda_1 = 6.5$ *μm along the edge1 direction and* $\lambda_2 = 4.3$ *μm along the edge2 direction. The polariton wavelength along edge1 (*λ_1*) is approximately 1.33 times larger than that along edge2 (*λ_2*), providing direct evidence of in-plane anisotropy. As summarized in Table S1, the quality factor (Q) along the two orthogonal edges are respectively 4.48 (edge1) and 4.86 (edge2) via normalizing the propagation length (*L_p*) in Figure S7b by the wavelength (*λ*), both falling within the lower range of values reported for quaternary oxides and Li/Na-*V_2O_5".

We believe these revisions address the reviewer's concerns comprehensively and present a more rigorous and transparent comparison of AMO's polaritonic performance.

Comment 13: The authors state "Line profiles (Figure 5c,d) quantify this evolution, showing a systematic reduction in fringe spacing as the frequency decreases. The central hotspot at 930 cm^{-1} has a full width at half maximum (FWHM) of approximately

633 nm, yielding a confinement factor (λ_{IR}/λ_p) of about 17—comparable to the value (50) previously reported for microcavity-modulated α-MoO_3 nanosheets.30”. However, in the current manuscript, ref. [30] corresponds to Duan et al., Adv. Mater. 2022, 34, 2104954, which is based on h-BN nanoantennas rather than α-MoO3 nanosheets. The authors must carefully verify all references to ensure that they correctly support the corresponding statements in the text. In addition, and as mentioned above, comparisons with other polaritonic materials should only be made under comparable experimental conditions (e.g., similar substrate, thickness, and geometry).

Our response: We sincerely apologize for this oversight. Upon verification, we confirm that Ref. [30] (Duan et al., Adv. Mater. 2022, 34, 2104954) indeed reports work on hBN nanoantennas, not α-MoO_3 nanosheet. In addition, we fully agree with the reviewer that direct comparisons of polaritonic figures of merit should only be made under comparable experimental conditions. To address this, we have removed the direct comparative statement from the main text. In the revised manuscript, page 16, line 308, we wrote “*The central hotspot at 930 cm^{-1} has a full width at half maximum (FWHM) of approximately 633 nm, yielding a confinement factor (λ_{IR}/λ_p) of about 17. This strong confinement underscores AMO's potential for sub-diffractional photonic devices such as superlenses or hyperlenses*”.

Comment 14: Throughout the manuscript, the authors directly interpret anisotropic polariton propagation as hyperbolic behavior. However, anisotropy alone does not imply hyperbolicity. The authors should explicitly demonstrate hyperbolic dispersion by extracting isofrequency curves (IFCs) or by determining the sign of the principal components of the dielectric tensor. Without this, the classification of the observed PhPs as hyperbolic remains ambiguous. In particular, the experimentally extracted dispersion from Fig. 4d suggests elliptical PhPs. The authors must clarify this.

Our response: We thank the reviewer for this constructive comment. In response, we have carried out a combined theoretical and experimental analysis to unambiguously determine the polaritonic nature of AMO.

Using density functional theory (DFT) calculations together with experimental data (including PiFM and Raman spectra measurements), we extracted the frequency-dependent in-plane and out-of-plane dielectric tensor components, ε_{xy} and ε_z of the AMO crystal. Based on this permittivity tensor, we constructed the three-dimensional isofrequency surfaces at 930 cm^{-1}. As shown in Figure **R9a**, the 3D IFC exhibits an open hyperboloid oriented along the k_z direction, directly demonstrating that AMO supports out-of-plane hyperbolic phonon polaritons.

To further clarify the polaritonic behavior in different propagation planes, we examined the projections of the 3D IFC. As shown in Figure **R9b**, the 3D IFC is projected onto the k_x-k_y plane. The contour is a closed ellipse, indicating that the in-plane dispersion is elliptical. This elliptical in-plane response is consistent with the anisotropic but non-hyperbolic propagation observed in the real-space near-field images and with the dispersion shown in Figure **4d**. Figure **R9c** shows the projection onto the k_x-k_z plane. The contour is an open hyperbola, confirming the out-of-plane hyperbolic character. Taken together, these results establish that AMO hosts a polaritonic regime of the in-plane elliptical and out-of-plane hyperbolic phonon polaritons.

The reviewer correctly notes that the experimentally extracted dispersion points in Figure **4d** appear to follow an elliptical trend. This observation is not contradictory to our assignment of hyperbolicity. Indeed, because the measurement in Figure **4d** probes only the in-plane wavevector, the dispersion of an out-of-plane hyperbolic material can indeed appear elliptical when only the in-plane component is considered.

To address the reviewer's concern and provide full transparency, we have revised the main text to explicitly state that AMO supports in-plane elliptical / out-of-plane hyperbolic phonon polaritons, and clarified that the dispersion in Figure **4d** corresponds to the in-plane projection, which is elliptical by nature. Figure **R9** is added as Figure **S10** in the Supporting Information describing the extraction of the permittivity tensor and the construction of 3D IFCs.

We believe these additions provide the explicit demonstration of hyperbolic dispersion requested by the reviewer and remove any ambiguity regarding the classification of the observed polaritons. We thank the reviewer again for pushing us to perform this critical analysis, which has substantially deepened the theoretical foundation of our work.

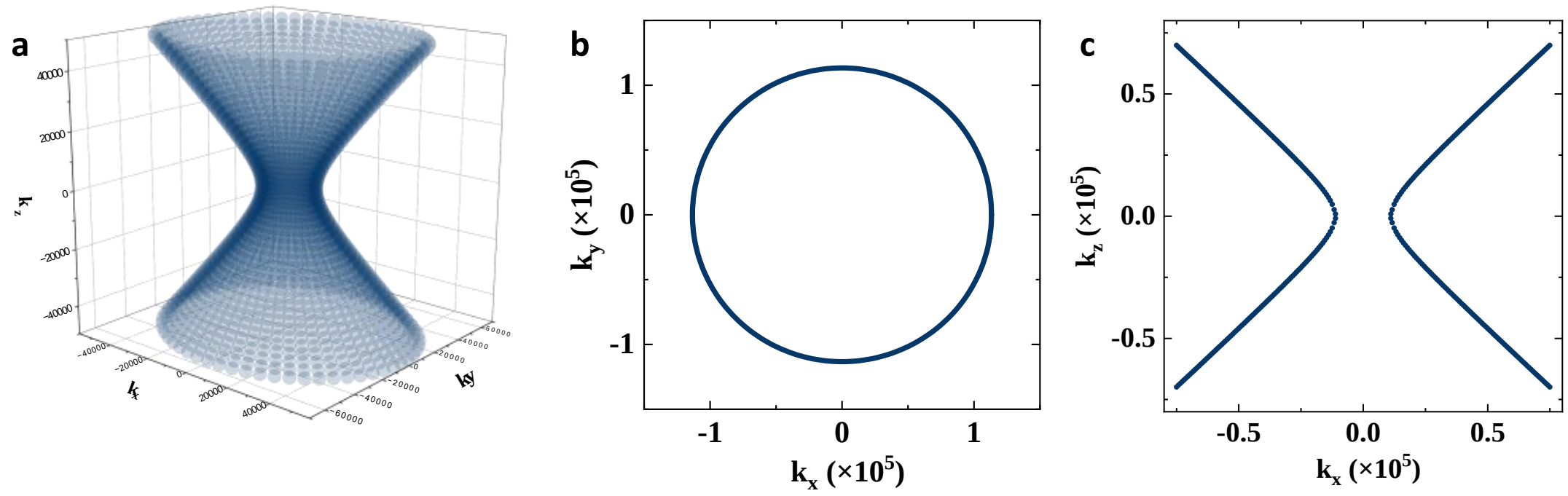

Figure R9. (a) 3D isofrequency surfaces at 930 cm^{-1} with ε_{xy} = -3.36 and ε_z = 3.763. (b) Projection of the IFC onto the k_x-k_y plane. (c) Projection of the IFC onto the k_x-k_z plane.

Comment 15: If AMO is to be proposed as a polaritonic material, a more exhaustive experimental and theoretical analysis is required. Exhaustive investigations of key polaritonic figures of merit—such as propagation length, group velocity, and lifetime—are currently missing. In addition, the authors should analyze the frequency-dependent complex PhP phasor along the [100] and [010] crystallographic directions (see, e.g., Nature Photonics 2015, 9, 674–678, Fig. 2cf and Nano Letters 2025, 25, 6,

2203–2209, Fig. 3g) to determine whether the observed anisotropic PhPs are hyperbolic or elliptical.

Our response: In the following, we address the reviewer's comments from both experimental and theoretical perspectives.

In response to the reviewer's concern regarding the demonstration of hyperbolic behavior in the phonon polaritons, we have calculated the permittivity tensor using DFT (Figure **R10a**). The resulting ab initio permittivity function qualitatively agrees with the experimental PiFM and Raman scattering spectra (Figure **R10b**). There is obvious discrepancy between the [110] and [001] directions in the range of 800-1100 cm^{-1}, the isofrequency surfaces in Figure **R10c**, defined by $\omega(k_{\perp}, k_{\parallel}) = \omega_0$ for a given frequency 930 cm^{-1}, indicates the hyperbolic nature of AMO crystal, i.e., in-plane elliptical phonon polaritons and out-of-plane hyperbolic phonon polaritons. As shown in Figure R10e-g, we calculated the wavenumber-dependent group velocity (v_g) and lifetime (τ). Near ν = 930 cm^{-1}, we obtained v_g = 0.007c and τ = 9.55 ps, using the method described in Ma et al. [*Nature* 2018, 562, 557–562].

we would like to mention that our current measurements are performed with photo-induced force microscopy (PiFM). Unlike scattering-type SNOM (s-SNOM) used in the referenced works, PiFM does not allow interferometric phase retrieval, nor does it support time-resolved or space-time mapping (e.g., Nature Photonics 2015, 9, 674–678, Fig. 2c,f). Therefore, we are unable to extract the phase evolution shown in Nano Letters 2025, 25, 2203–2209, Fig. 3g, or to directly measure group velocity and lifetime as done by Yoxall et al. Given these technical constraints, we have instead performed extensive first-principles calculations (DFT) to compute the isofrequency contours and dispersion of the polaritonic modes in AMO, which unambiguously confirm the hyperbolic nature of the phonon polaritons.

To clarify the role of biaxial strain in modulating the polaritonic response along

edge 1 and edge 2, we qualitatively extended the analytical model to incorporate the effect of uniaxial compressive and tensile strain along the two orthogonal in-plane directions. The strain was applied by modifying the relevant phonon frequencies according to the strain-induced shift derived from our lattice mismatch calculations (-2.87% and +2.86%). As shown in Figure **R10d**, the dispersion curve shifts to higher frequencies by approximately +14 cm^{-1} under tensile strain relative to the unstrained case (black). While the dispersion curve shifts to lower frequencies by approximately -14 cm^{-1} under compressive strain. These opposite shifts directly demonstrate that the anisotropic strain imposed by the sapphire substrate can split the degenerate dispersion branches and induce a tunable anisotropic response. This result strongly supports our claim that the observed 33% anisotropy in polariton propagation originates from biaxial strain.

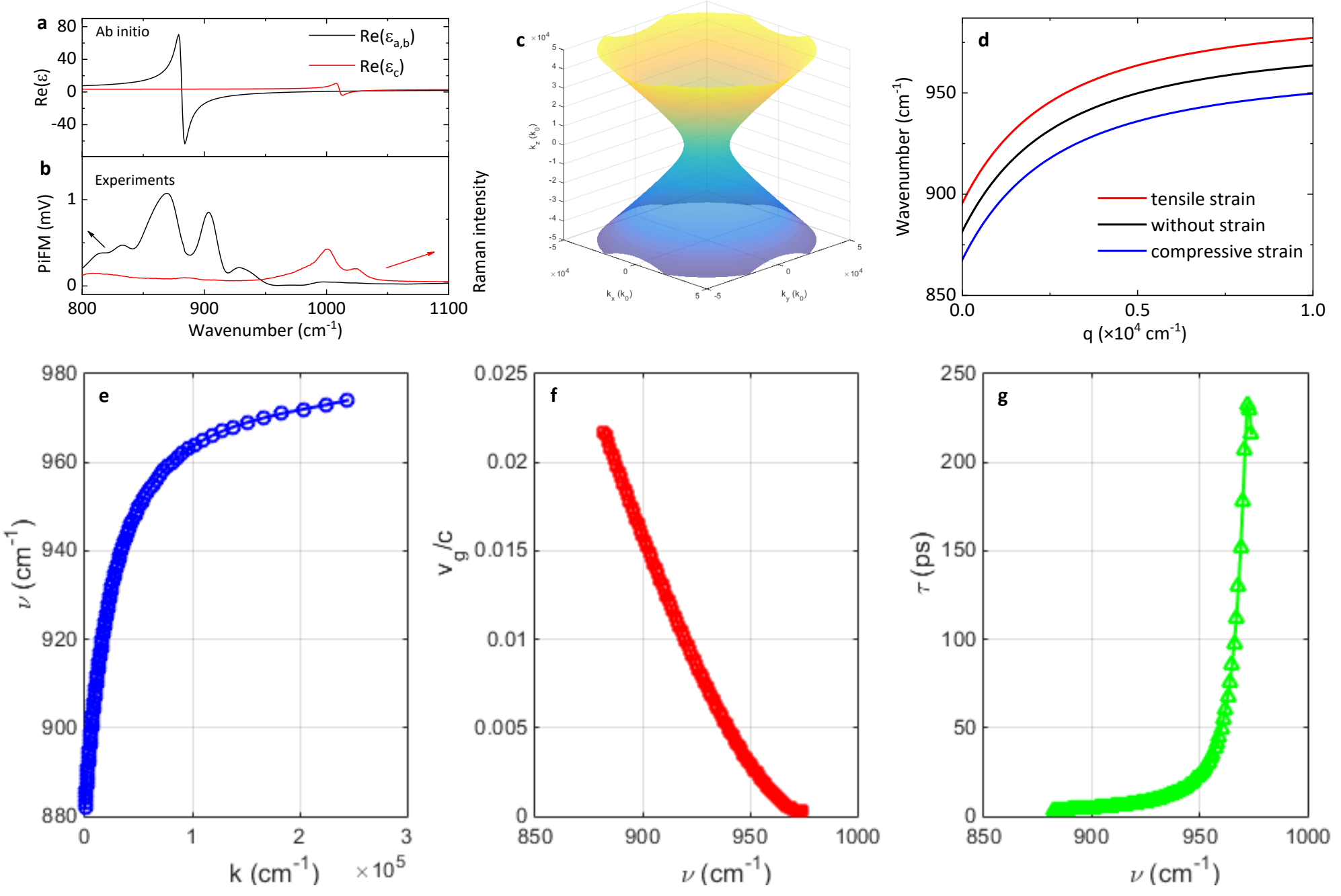

Figure R10. (a) Ab initio permittivity of AMO crystal along the *ab*-[110] and *c*-[001] axes. (b) PiFM (black) and Raman scattering (red) spectra of the as-grown AMO crystal. (c) Isofrequency surfaces defined by $\omega(k_\perp, k_{//}) = \omega_0$ for a given frequency 930 cm^{-1}, in which $\varepsilon_{a,b}$ = -3.346, ε_c = 3.763. (c) Calculated dispersion curves (frequency vs. in-plane wavevector) with tensile and compressive strain. (e-g) Calculated dispersion curve,

group velocity, and lifetime, respectively.

Comment 16: The PiFM results are not supported by analytical or numerical modeling. Given that AMO is biaxial and sapphire permittivity is well known, the authors could model the PhP dispersion using established analytical frameworks (e.g., Phys. Rev. B 2019, 100, 235408) or FEM simulations. Such modeling is essential to validate the extracted dispersions and to confirm the claimed hyperbolic behavior.

Our response: We thank the reviewer for this suggestion. We fully agree that analytical or numerical modeling is essential to validate the experimental dispersions and to confirm the hyperbolic nature of the polaritons. In response, we have performed both analytical and numerical simulations as described below. Following the established framework for biaxial thin films on a substrate (e.g., Phys. Rev. B 2019, 100, 235408), we modeled the phonon polariton (PhP) dispersion of the AMO/sapphire system. The AMO permittivity tensor was obtained from our PiFM, Raman scattering measurements and DFT calculations. The calculated dispersion curves (frequency vs. in-plane wavevector) are presented in Figure **R11**. The analytical solution (dashed curves) shows excellent agreement with the experimentally extracted dispersion points (blue spherical symbol) from Figure **4d**, thereby validating the experimental wavevector–frequency relationship.

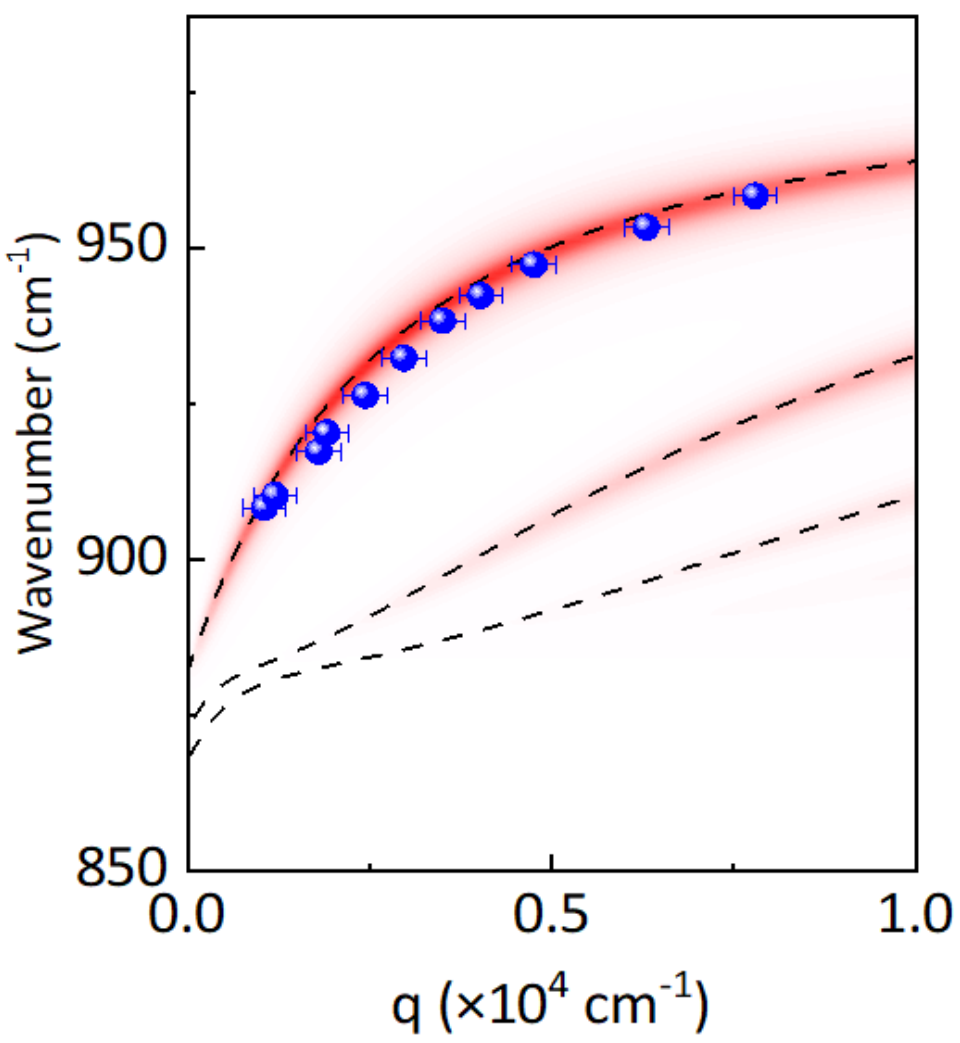

Figure R11. Experimental (blue dots) and calculated dispersion curves (red shaded region), with the analytical solution overlaid as black dashed curves.

Comment 17: Since the authors propose AMO as a new mid-IR nanophotonic material, Raman spectroscopy alone is insufficient. FTIR spectroscopy should be performed to identify the transverse optical (TO) and longitudinal optical (LO) phonon modes. This information is essential to extract and fit the infrared dielectric permittivity tensor of AMO, which is a prerequisite for any quantitative polaritonic analysis.

Our response: We thank the reviewer for this suggestion. In response, we have performed polarized FTIR measurements on AMO flakes grown on sapphire. However, as shown in Figure **R12**, the FTIR spectrum of the AMO/sapphire sample is almost identical to that of the bare sapphire substrate. We attribute this to two factors: (i) the AMO nanoflakes are randomly distributed on the sapphire surface with a relatively low surface coverage and small thickness, resulting in a weak overall signal. (ii) the strong infrared phonon bands of the sapphire substrate dominate the measured signal, effectively masking the contribution from the AMO flakes.

Given this experimental difficulty, we have taken an alternative, multi-pronged approaches (e.g., DFT calculation, PiFM and Raman spectra, etc.) to determine the dielectric permittivity tensor and to validate the polaritonic response of AMO (Figure **R10**). The consistency among these three independent approaches – DFT, PiFM, and Raman – gives us high confidence that the derived dielectric permittivity tensor and the resulting polariton dispersion are reliable, despite the difficulty in obtaining clear polarized FTIR data on the sapphire substrate.

We acknowledge the reviewer's valid point that direct FTIR measurement on a mid-IR transparent substrate would be the ideal approach. In future work, we will explore the transfer of AMO flakes onto low-loss mid-IR transparent substrates (e.g.,

CaF_2 or BaF_2) to avoid substrate absorption and to enable high-quality polarized FTIR spectroscopy. This will allow us to directly measure the TO/LO modes and to further refine the dielectric tensor.

Given this experimental difficulty, we have taken an alternative approach that combines DFT calculations (Figure **R12b**), PiFM and Raman spectra (Figure **R12c**) to obtain the TO/LO frequencies and the dielectric permittivity tensor (Table **R2**). The consistency among these three independent approaches gives us high confidence in the derived dielectric permittivity tensor and the identification of TO/LO modes. As shown in Figure **R12d**, the TO and LO frequencies are clearly labeled.

We thank the reviewer again for pushing us to address this important characterization, and we believe that the combination of DFT, PiFM and Raman provides a robust and cross-validated determination of the dielectric properties of AMO.

Table **R2**

***a,b*-[110]**					***c*-[001]**				
**Band**	TO	LO	Width	(Im)TO	**Band**	TO	LO	Width	(Im)TO
**1**	346.7	365.6	18.9	31.2	**1**	293.6	299.7	6.1	21.6
**2**	407.3	410.6	3.3	12.2	**2**	371.5	384.4	12.9	33.7
**3**	461.1	464.4	3.3	31.3	**3**	453.8	486.1	32.3	70.6
**4**	474.1	525.4	51.3	34.3	**4**	505.7	535.9	30.2	20.9
**5**	530.4	543.5	13.1	3.1	**5**	874.2	883.0	8.8	45.1
**6**	842.3	845.2	2.9	20.0	**6**	909.8	997.8	88.0	84.1
**7**	881.5	976.2	94.7	66.9	**7**	1010.4	1021.6	11.2	1.8
**8**	997.9	1007.9	9.9	2.5					

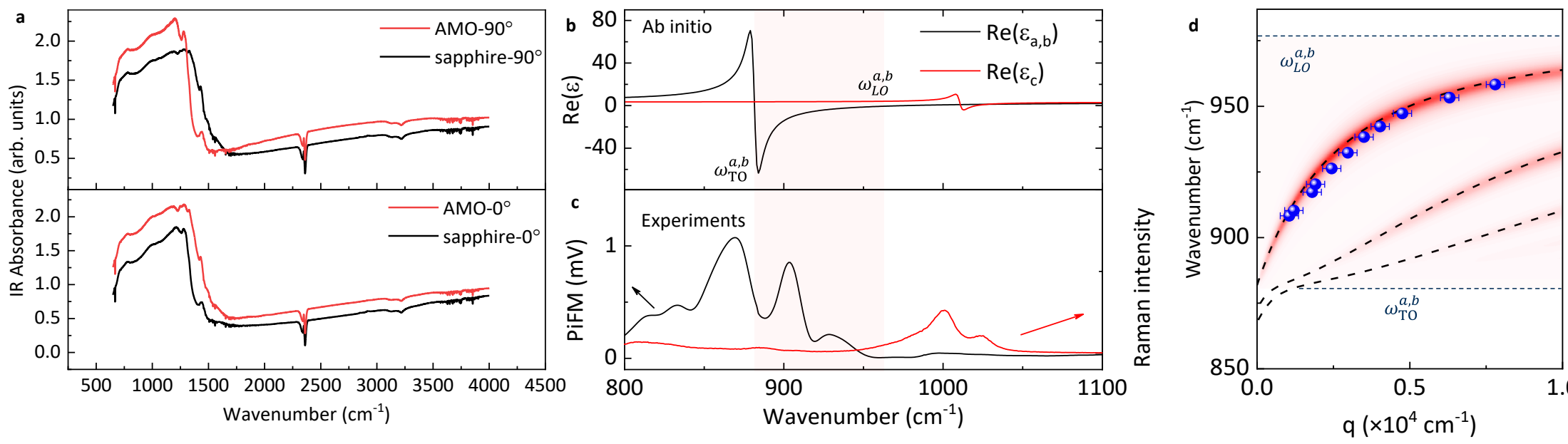

Figure R12. (a) The absorbance of IR spectra measured by FTIR for the polarization (top panel) *E*// z modes and (bottom panel) *E*// xy modes of AMO flakes. (b) Ab initio permittivity of AMO crystal along the *ab*-[110] and *c*-[001] axes. (c) PiFM (black) and Raman scattering (red) spectra of the as-grown AMO crystal. (d) Experimental (blue dots) and calculated dispersion curves (red shaded region), with the analytical solution overlaid as black dashed curves. ω_{TO} = 881.5 cm^{-1} and ω_{LO} = 976.2 cm^{-1}are marked as transverse optical (TO) and longitudinal optical (LO) phonon modes, respectively.

Comment 18. All near-field experiments are performed on sapphire substrates, which exhibit strong phonon resonances in the same spectral range as the reported AMO Reststrahlen band. This might potentially induce substrate-induced artificially enhanced propagation lengths. The authors should clarify whether the AMO crystals can be mechanically picked up and transferred onto alternative substrates commonly used in mid-IR nanophotonics (e.g., Si, SiO2, CaF2, BaF2). If transfer is feasible, the authors should perform PiFM measurements on mid-IR transparent substrates (CaF2 or BaF2) to isolate the intrinsic polaritonic properties of AMO.

Our response: We thank the reviewer for this suggestion. We attempted to mechanically exfoliate and transfer AMO flakes from the original sapphire growth substrate onto alternative substrates, including Au-coated SiO2/Si and mid-IR transparent CaF_2. The procedure involved using a dry-transfer method with a polydimethylsiloxane (PDMS) stamp, which is commonly used for van der Waals materials. Key observations are as follows:

- Flake fracture and fragmentation: Attempts to mechanically lift the flakes using PDMS stamps or adhesive tape resulted in fracture and fragmentation. In most cases, only small triangular fragments (< 5 μm in lateral size) could be transferred (Figure **R13a**), which are insufficient for PiFM measurements of polariton propagation (which require flakes with dimensions of at least 10–20 μm to clearly resolve fringe patterns).
- Strong adhesion to sapphire: Unlike typical van der Waals crystals (e.g., h-BN, MoS_2, or α-MoO_3), which can be mechanically exfoliated, AMO flakes exhibit exceptionally strong bonding to the sapphire substrate (Figure **R13b**). This is consistent with the formation mechanism of AMO: as discussed in the main text, AMO grows via an interfacial reaction between MoO_3 and the Al_2O_3 substrate. This chemical reaction likely creates a robust interfacial bonding, rather than the weak van der Waals interactions typical of exfoliable materials.

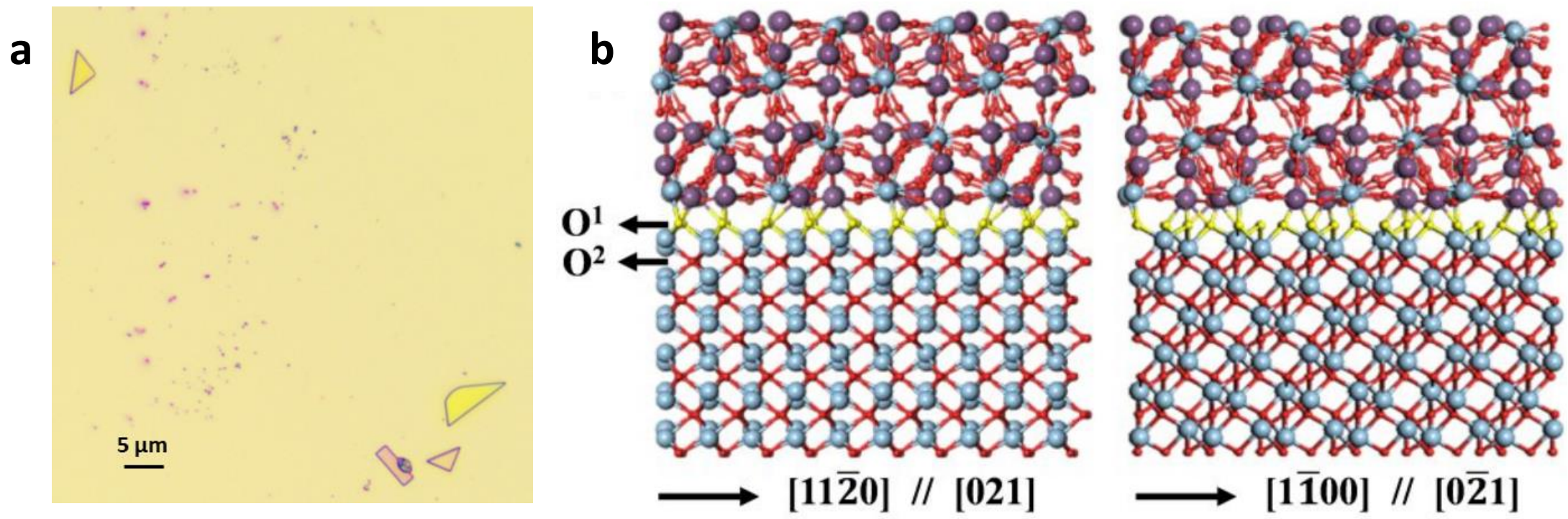

Figure R13. (a) Optical microscopy image of AMO flakes transferred on Au coated SiO2/Si substrate. (b) Side view of AMO along sapphire [1120] and [1**1**00] direction from Figure S10 in reference (J.D. Bai, et al., *J. Phys. Chem. Lett.* **2025**, 16,3689−3697.

To reflect these efforts and limitations, we have added the following text to the Methods section, page 19, line 353: "*We attempted to transfer AMO flakes onto alternative substrates (CaF_2, Au-coated Si) using dry-transfer methods. However, due to the strong chemical bonding at the AMO/sapphire interface resulting from the in-*

situ growth reaction, the flakes proved resistant to exfoliation and frequently fractured upon attempted transfer. Only small fragments (<5 μm) could be obtained, precluding polariton imaging. This strong adhesion contrasts with the weak van der Waals interactions in typical exfoliable 2D materials and underscores the unique growth mechanism of AMO".

Comment 19. A very important note: The tunability attributed to biaxial strain and lateral size in Table 1 is not supported by convincing experimental or theoretical evidence. Strain is inferred from epitaxial mismatch but is neither measured nor varied, and no strain-dependent phonon or polariton shifts are reported. Additionally, the influence of lateral size is discussed without separating it from thickness effects. In its current form, the table suggests a level of material tunability that is not demonstrated in the manuscript.

Our response: We thank the reviewer for this constructive comment. To address the reviewer's concern about the lack of demonstrated tunability, we have performed a systematic analysis of polariton propagation in AMO flakes of varying thicknesses (Figure **R14**). The results are summarized below:

Flake thickness (nm)	Polaritonic wavelength (μm)	Anisotropic ration
470	4.3	1.33
398	3.69	1.38
283	2.61	1.45
247	2.25	1.49
132	1.12	1.53

As shown, the anisotropy ratio decreases systematically with increasing flake thickness, from 1.53 for 132 nm flakes to 1.33 for 470 nm flakes. This trend is consistent with the expected relaxation of epitaxial strain in thicker flakes. These data

provide direct experimental evidence that the polaritonic properties of AMO can be tuned by varying the flake thickness, which in turn modulates the residual strain state and the effective dielectric environment.

We have included these new data in the revised manuscript as Figure **S9** and discussed them in the Results section.

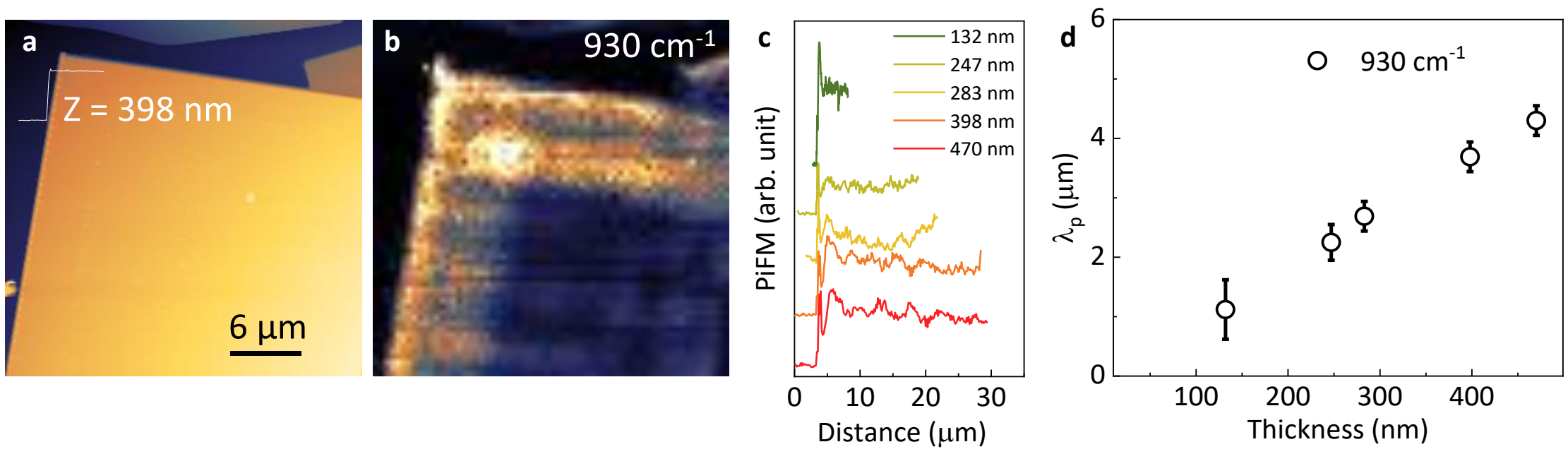

Figure R14. (a) A typical AMO flake with a thickness *t* = 398 nm. (b) The corresponding PiFM image at 930 cm^{-1}. (c) Phonon polariton line profiles for AMO crystal with different thickness. (d) Wavelengths of phonon polaritons probed at 930 cm^{-1} for crystals with different thickness.

Comment 20: In the discussion of Fig. 4, the authors refer to the observation of "edge modes," based on the fringe patterns visible in the panels of Fig. 4b. However, the features observed in these panels correspond to edge-launched or edge-reflected phonon polaritons rather than true edge modes (for real edge-modes see Nature Photon 2016, 10, 239–243). The authors should revise the text accordingly and use the correct terminology (see, e.g., Nano Letters 2017, 17, 9, 5285–5290). In addition, the authors should carefully check the fitting procedures of the near-field line profiles reported in the Supporting Information.

Our response: We thank the reviewer for this terminological clarification. We have carefully consulted the recommended references [Nature Photon. 2016, 10, 239 – 243; Nano Lett. 2017, 17, 5285 – 5290] and fully agree with the distinction drawn by

the reviewer. The fringe patterns observed in Figure **4b** arise from edge-launched phonon polaritons rather than true "*edge modes*". We have revised the manuscript accordingly: All instances of "*edge modes*" in the main text and figure captions have been replaced with "*edge-launched PhPs*".

In the discussion of Figure **4**, page 13, line 237, we now explicitly state: "*Spatial imaging of a large AMO flake from 910 to 950 cm^{-1} revealed edge-launched PhPs, which are generated by scattering of the incident field at the sample edges (Figure 4b), where the interference pattern varies dramatically with excitation frequency. Crucially, these edge-launched PhPs exhibit distinct periodicities along the two principal crystal axes*".

In response to the reviewer's additional request, we have carefully re-examined the fitting procedures for the near-field line profiles reported in the Supporting Information. We confirm that the exponential decay fits used to extract propagation lengths were performed on line profiles averaged over multiple fringes in Figure **S7** to minimize noise.

Comment 21: While edge-launched PhP are observed, their frequency dependence and physical origin are not sufficiently analyzed. A systematic study of PhP dispersion and its dependence on strain and crystal orientation is needed. At present, the role of strain in shaping edge modes remains qualitative.

Our response: We thank the reviewer for this important comment. We have performed a comprehensive set of experiments and theoretical analyses to address this issue, as detailed below.

1. Thickness-dependent strain and its effect on PhP propagation (Figure **R14**).

Because the epitaxial strain imposed by the sapphire substrate is expected to relax with increasing flake thickness, we carried out PiFM measurements on AMO

flakes of different thicknesses (ranging from ~132 nm to ~470 nm), all with lateral dimensions >30 × 30 μm² to avoid geometric focusing effects. As shown in Figure **R14**, the in-plane anisotropy ratio (λ_1/λ_2) decreases systematically from ≈1.53 for a 132 nm flake to ≈1.33 for a 470 nm flake, while the propagation length increases slightly. This thickness-dependent trend directly correlates with progressive strain relaxation and provides quantitative evidence that biaxial strain modulates the polaritonic response.

2. Orientation-dependent polariton anisotropy revealed by adjacent nanoflakes (Figure **R15**).

To directly test whether the polariton anisotropy varies with crystal orientation (and thus with the orientation-dependent strain state on sapphire), we selected four neighboring AMO nanoflakes with different in-plane rotations. PiFM images acquired at 940 cm^{-1} show that the central rectangular hot spot rotates systematically with the flake orientation, see Figure **R15** (Figure **S8** in SI). For four adjacent AMO nanoflakes, the aspect ratios of the central rectangular hot spots extracted from PiFM images (E_1/E_2, ranging from 1.49 to 1.58) are systematically larger than the corresponding geometric aspect ratios measured from AFM topography (L_1/L_2, ranging from 1.06 to 1.27). This discrepancy indicates that the observed shape anisotropy of the hot spot cannot be solely attributed to the flake geometry. Instead, it reflects an additional contribution from strain-induced in-plane dielectric anisotropy, which locally modifies the polariton focusing behavior.

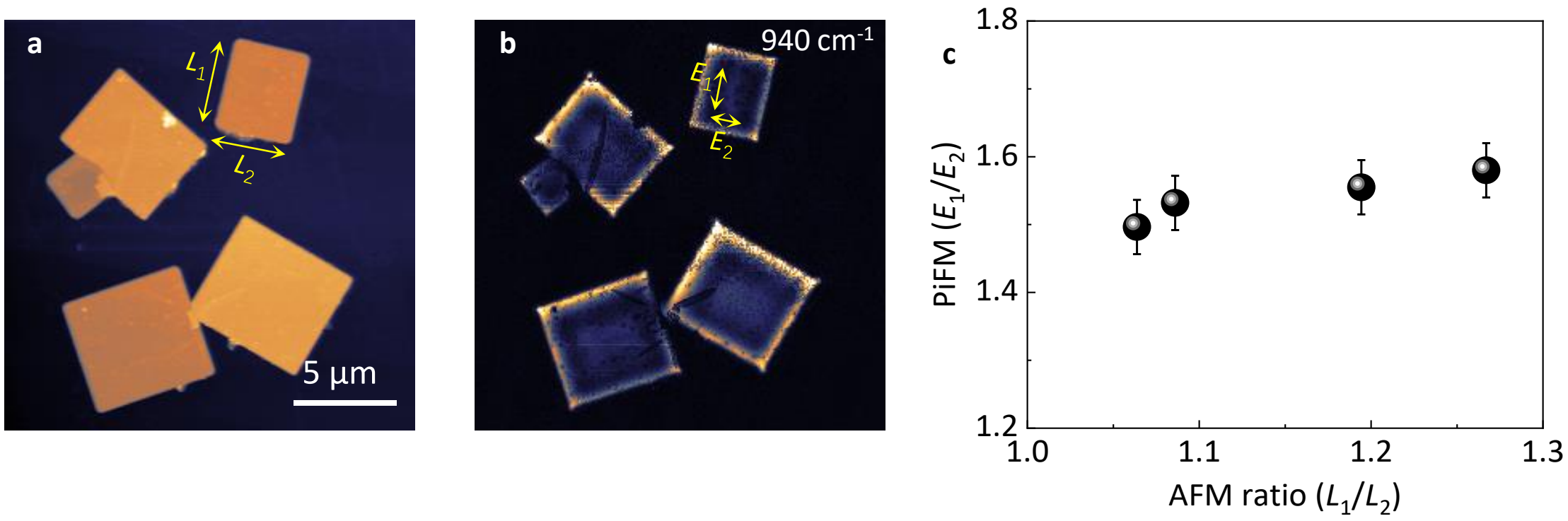

Figure R15. (a) Atomic force microscopy (AFM) of AMO flakes. (b) The corresponding PiFM image at 940 cm^{-1}. (c) Aspect ratios of the central rectangular hot spots extracted from PiFM images (E_1/E_2) vs the corresponding geometric aspect ratios (L_1/L_2).

3. Numerical modeling of strain-induced dispersion shifts (Figure **R10d**).

Using the analytical framework for biaxial thin films on a substrate (Phys. Rev. B 2019, 100, 235408) together with the experimentally determined permittivity tensor of AMO, we calculated the PhP dispersion curves under zero strain, uniaxial tensile strain, and uniaxial compressive strain (the latter two corresponding to the lattice mismatches of +2.86% and −2.87% derived from epitaxial relationships). The results show that tensile strain shifts the dispersion curve to higher frequencies by approximately +14 cm^{-1}, while compressive strain shifts it to lower frequencies by approximately −14 cm^{-1} relative to the unstrained case. These opposite shifts qualitatively explain the observed anisotropic propagation.

Collectively, the thickness-dependent experiments, the orientation-dependent near-field imaging of adjacent flakes, and the strain-tuned numerical dispersions provide quantitative, multi-faceted evidence that biaxial strain from the sapphire substrate is the primary origin of the observed polariton anisotropy in AMO. We have demonstrated the systematic influence on both propagation length and dispersion shift. We believe these quantitative analyses have substantially strengthened the paper.

Comment 22. Sapphire is not a standard substrate for nanophotonic device integration. If AMO flakes are transferred to other substrates, it is unclear whether the epitaxial strain—and thus its reported influence in anisotropic polaritonic

behavior—would be preserved. This point should be discussed to assess the practical relevance of strain-engineered AMO.

Our response: We thank the reviewer for raising this point. We fully agree that the ability to transfer AMO flakes onto other substrates while preserving (or controllably modifying) the strain state is essential for future device applications. Below we discuss our attempts, the current challenges, and a clear outlook for future work.

1. Challenges in transferring AMO flakes (**Figure R13**).

As described in our response to an earlier comment, we have made extensive efforts to mechanically exfoliate and transfer AMO flakes from the native sapphire substrate onto other substrates (e.g., Au-coated Si). However, unlike typical van der Waals materials (e.g., graphene, hBN, or α-MoO_3), AMO grows via chemical reactions between MoO_3 vapor and the Al_2O_3 substrate, forming strong covalent/ionic bonds at the interface. Consequently, the flakes adhere tenaciously to sapphire and almost invariably fracture upon attempted lift-off. In rare cases where small triangular fragments (typically < 5 μm) could be transferred, their lateral dimensions were insufficient for PiFM imaging of propagating polaritons (which requires flakes of at least 20 μm to clearly resolve interference fringes). Therefore, we cannot currently assess whether the epitaxial strain would be preserved after transfer.

2. Why is AMO so strongly bonded to sapphire?

We attribute this strong adhesion to the epitaxial growth mechanism itself. As demonstrated by the XRD (only (00*l*) peaks), AMO nucleates and grows through a solid-state reaction at 750–800 °C, where MoO_3(g) reacts with the sapphire surface to form $Al_2(MoO_4)_3$. This chemical epitaxy leads to an interface with mixed covalent/ionic character, unlike the weak van der Waals interactions in exfoliable layered crystals. As a result, the as-grown AMO flakes behave more like an epitaxial film than a transferrable 2D material.

3. Future directions to address the reviewer's concern.

We fully acknowledge that the practical utility of strain-engineered AMO will ultimately require integration onto device-compatible substrates. Therefore, we have identified this as a priority for our follow-up research. In future work, we plan to use selective etching of the sapphire substrate (e.g., with a hot H_3PO_4 solution) to release AMO membranes, followed by dry transfer to a target substrate. This systematic study, however, constitutes a substantial research effort in their own right and are beyond the scope of the current discovery-oriented work, which focuses on the first demonstration of AMO as a polaritonic material and the fundamental role of strain in its anisotropic response.

To address the reviewer's request for transparency, we have added a brief discussion in the outlook section of the revised manuscript (page 14, line 259):

"*We note that the potential role of strain in modulating the edge-launched polariton modes observed here remains qualitative. A systematic, quantitative analysis of strain effects would require controlled strain experiments combined with direct strain measurements, which we identify as an important direction for future investigation.*"

We believe this manuscript has been improved significantly according to above valuable comments and suggestions, and hope now it is suitable for publication in Nature Communications.

For Reviewer #3:

Reviewer #3: The authors present an efficient method for synthesizing thin layers of aluminium molybdate (AMO), provide a detailed characterization of these layers, and demonstrate the propagation of phonon polaritons (PhPs) within layers of this material. The paper is solid, well-written, and clear. The section on the synthesis and basic characterization of the material provides a comprehensive description of the synthesis procedure, leaving no doubt regarding the structure and quality of the resulting samples. However, the final section, devoted to the study of PhPs in AMO, requires some revision. Overall, I believe the paper is interesting and valuable to the scientific community. However, the following questions should be addressed before the paper can be accepted for publication.

Our response: We thank the reviewer for the positive assessment of our work and for recognizing the value of our synthesis approach and material characterization. We are pleased that the reviewer found the paper "*solid, well-written, and clear, interesting and valuable to the scientific community*".

Below, we provide a point-by-point response to each of the reviewer's questions and describe the corresponding revisions made to the manuscript. We believe these modifications have substantially improved the clarity and rigor of our polaritonic analysis.

Comment 1: The authors claim that the in-plane anisotropy of phonon polaritons is related to the presence of in-plane compression and tension of the material along different directions (specifically on page 13, lines 234-236). However, in my opinion, this statement does not appear fully substantiated. Indeed, the AMO itself is already structurally anisotropic in the plane; consequently, optical anisotropy can be expected even in the absence of strain, as in other biaxial materials. Therefore, it remains

unclear to what extent the anisotropy of phonon polariton propagation is due to structural anisotropy and to what extent to the presence of strain.

Our Response: we thank the reviewer for raising this fundamental question regarding the origin of the observed polaritonic anisotropy in AMO. The reviewer correctly notes that AMO is a structurally anisotropic material (monoclinic, $a \neq b$, $\alpha = 90°$), and thus one might naturally expect optical anisotropy even in the absence of strain. However, our experimental observations present a compelling case that the dominant source of the observed anisotropy in our specific samples is epitaxial strain imposed by the sapphire substrate, rather than the intrinsic lattice anisotropy. Below we substantiate this claim with crystallographic evidence and quantitative strain analysis.

1. Crystallographic orientation of the AMO flakes:

As shown in Figure **R16**, the AMO flakes grow with their [100] and [010] crystallographic axes oriented along the diagonals of the square-like flake morphology. Critically, this means that the two orthogonal edges of the square-like flakes—along which we measure polariton propagation—are crystallographically equivalent. In other words, while the AMO unit cell itself has $a \neq b$, the physical edges of our flakes are aligned along directions that are symmetric with respect to the crystal axes. If intrinsic structural anisotropy (a vs. b) were the dominant factor, we would expect no difference in polariton propagation along these two equivalent directions. Yet, we observe a clear asymmetry in edge-launched PhPs behaviors, indicating that an external perturbation—namely, epitaxial strain—must be breaking this symmetry.

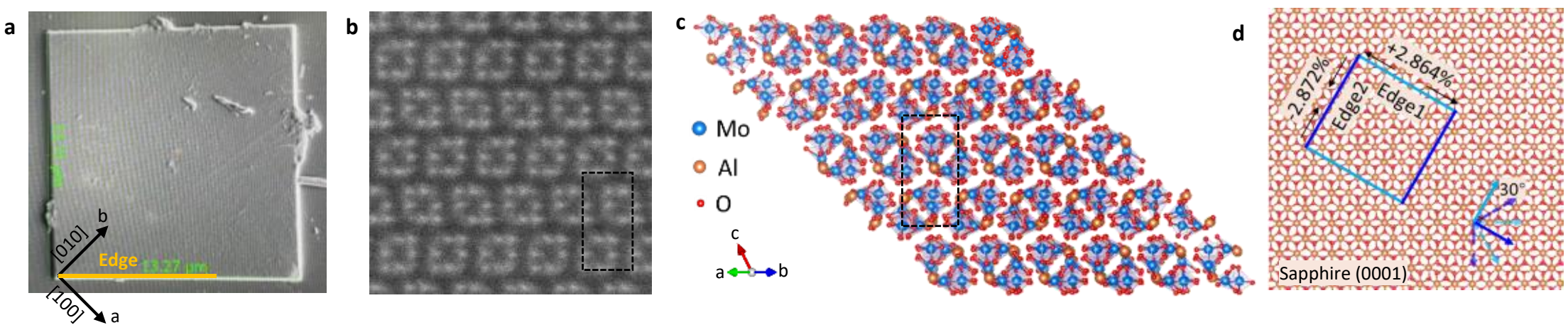

Figure R16. (a) Scanning electron microscope image of a AMO flake. Yellow solid line is used to mark the edge, where the cross-sectional HRTEM was performed. (b) Cross-sectional high-angle annular dark-field STEM image of AMO flake. (c) Atomic structure projected along the (110) plane. (d) Epitaxial relationship between AMO and sapphire (0001).

2. Quantitative strain analysis from lattice mismatch:

To quantify this effect, we have calculated the lattice mismatch between AMO and the underlying sapphire (0001) substrate. Based on the orientation relationship determined from electron diffraction, the mismatch along the two orthogonal in-plane directions is: Along edge1: +2.864% (tensile strain); Along edge2: -2.872% (compressive strain). These values are nearly equal in magnitude but opposite in sign, representing a pure shear strain that breaks the in-plane symmetry. Such a strain state is expected to induce significant modifications to the phonon frequencies and, consequently, the polaritonic response.

Comment 2: In fact, the observed fringe period does not always correspond to the polariton wavelength. The error arises when the polariton wavelength is comparable to the wavelength in vacuum, which is the case in this study, unlike many other mid-IR experiments, where the polariton wavelength can be tens of times shorter than the free-space wavelength. With such a long wavelength, the sample irradiation direction can influence the fringe length, thereby increasing or decreasing the observed

anisotropy. A method for correctly determining the polariton wavelength, taking into account the beam incidence direction, is described in the following paper: Nature Photon 11, 356–360 (2017). These corrections may not be very large in this case, but it is reasonable to estimate them.

Our Response: We thank the reviewer for this important methodological note. In our study, the free-space wavelength at 930 cm^{-1} is $\lambda_0 \approx 10.75$ μm, and the polariton wavelength extracted from the fringe pattern is $\lambda_p \approx 4.3$ μm (from Fig. 4d). Thus $\lambda_p/\lambda_0 \approx 0.4$, i.e., the polariton wavelength is about one order of magnitude shorter than the free-space wavelength, but still not in the extreme confinement regime (where $\lambda_p \ll \lambda_0$). Therefore, a correction is indeed advisable. Following the method described in the recommended paper, we have estimated the correction to the polariton wavelength due to the oblique incidence geometry. In our PiFM setup, the incident laser beam is focused onto the tip at an angle of approximately $\theta \approx 30°$ relative to the sample surface (i.e., $60°$ from the normal). The in-plane component of the incident wavevector is $k_{\parallel} = k_0 \cos\theta$ (with $k_0 = 2\pi/\lambda_0$). Because our fringe analysis is based on line profiles taken perpendicular to the edge with the incident beam perpendicular to that edge, the forward and backward launched polaritons contribute equally to the interference pattern, so the effective correction is actually smaller (the $k_{\parallel}$ term cancels in the averaging). We estimate the net correction to λ_p to be on the order of 5% or less. Applying this correction would change our reported λ_p and correspondingly the quality factor $Q = L_p/\lambda_p$ by a similar modest amount. Importantly, these minor adjustments do not alter any of our qualitative conclusions (e.g., the anisotropy ratio, the thickness-dependent trends, or the hyperbolic classification). The main findings of the manuscript remain robust.

To ensure transparency, we have added a brief discussion of this correction in the “Methods section” of the revised manuscript, page 21, line 388, citing Hu et al. (*Nature Photonics* 2017) as reference [50] and noting that the correction is modest (within ±5–10%) and does not affect the key conclusions. We thank the reviewer again for drawing our attention to this subtle but important correction, which has improved the quantitative accuracy of our work.

Comment 3: The obtained data allows to estimate the principal components of the in-plane permittivity tensor. Indeed, in the two-dimensional conductivity (ultrathin layer) approximation (Nature 562, 557–562 (2018), Phys. Rev. B 100, 235408 (2019)), the out-of-plane component of the permittivity tensor can be eliminated from the dispersion equation, allowing for a unique relationship between the wavevector and the in-plane component of the permittivity tensor. This result may help substantiate the nature of in-plane anisotropy.

Our response: We thank the reviewer for this constructive and insightful suggestion. The two-dimensional conductivity (ultrathin layer) approximation assumes the flake thickness to be much smaller than the polariton wavelength. It indeed work for the cited works [*Nature* 2018, 562, 557–562; *Phys. Rev. B* 2019, 100, 235408], which are atomically thin or few-layer van der Waals materials. However, it may not applicable to our AMO flakes, which have thicknesses of 470 nm (Figure 4) and 283 nm (Figure 5).

In our work, we have taken a complementary, multi-pronged approach to determine the permittivity tensor, including the first-principles DFT calculations, experimental PiFM & Raman spectra (Figure **R17a, b**). From the refined permittivity tensor, we constructed the three-dimensional IFCs (Figure **R17c**). The projection onto the $k_x{-}k_y$ plane (Figure **R17d**) reveals elliptical contours, while the projection onto the $k_x{-}k_z$ plane (Figure **R17e**) shows hyperbolic contours. This

unambiguously demonstrates that AMO supports in-plane elliptical and out-of-plane hyperbolic phonon polaritons.

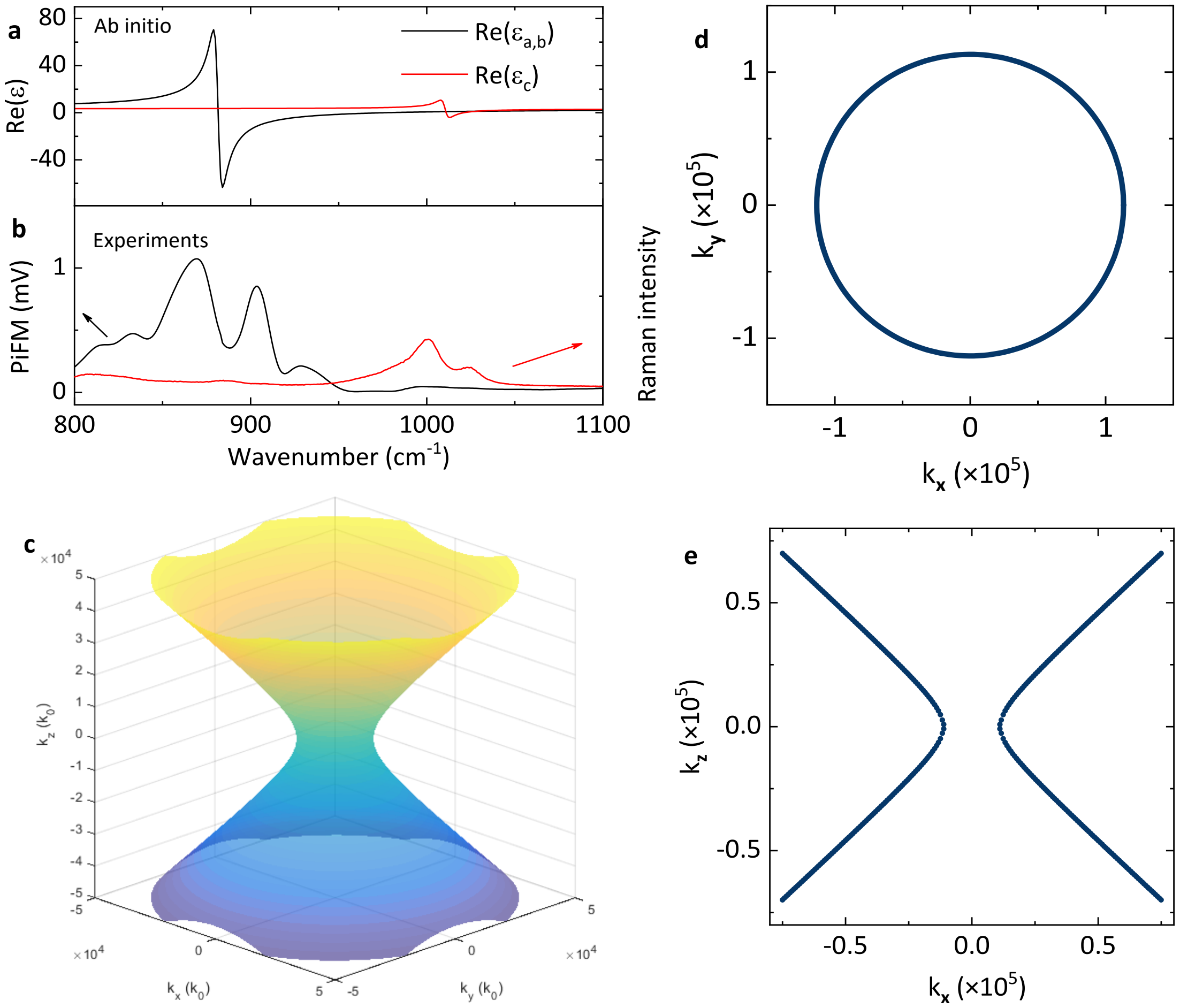

Figure R17. (a) Ab initio permittivity of AMO crystal along the *ab*-[110] and *c*-[001] axes. (b) PiFM (black) and Raman scattering (red) spectra of the as-grown AMO crystal. (c) Isofrequency surfaces (IFC) defined by $\omega(k_\perp, k_{//}) = \omega_0$ for a given frequency 930 cm^{-1}, in which $\varepsilon_{a,b} = -3.346$, $\varepsilon_c = 3.763$. (d) Projection of the isofrequency surface onto the k_x-k_y plane. (e) Projection of the isofrequency surface onto the k_x-k_z plane.

As shown in Figure **R18**, the dispersion curve shifts to higher frequencies by approximately +14 cm^{-1} under tensile strain relative to the unstrained case. While the dispersion curve shifts to lower frequencies by approximately -14 cm^{-1} under

compressive strain, and the unstrained dispersion (black) lies in between. These opposite shifts directly demonstrate that the anisotropic strain imposed by the sapphire substrate can split the degenerate dispersion branches and induce a tunable anisotropic response. This result qualitatively supports our claim that the observed 33% anisotropy in polariton propagation originates from biaxial strain.

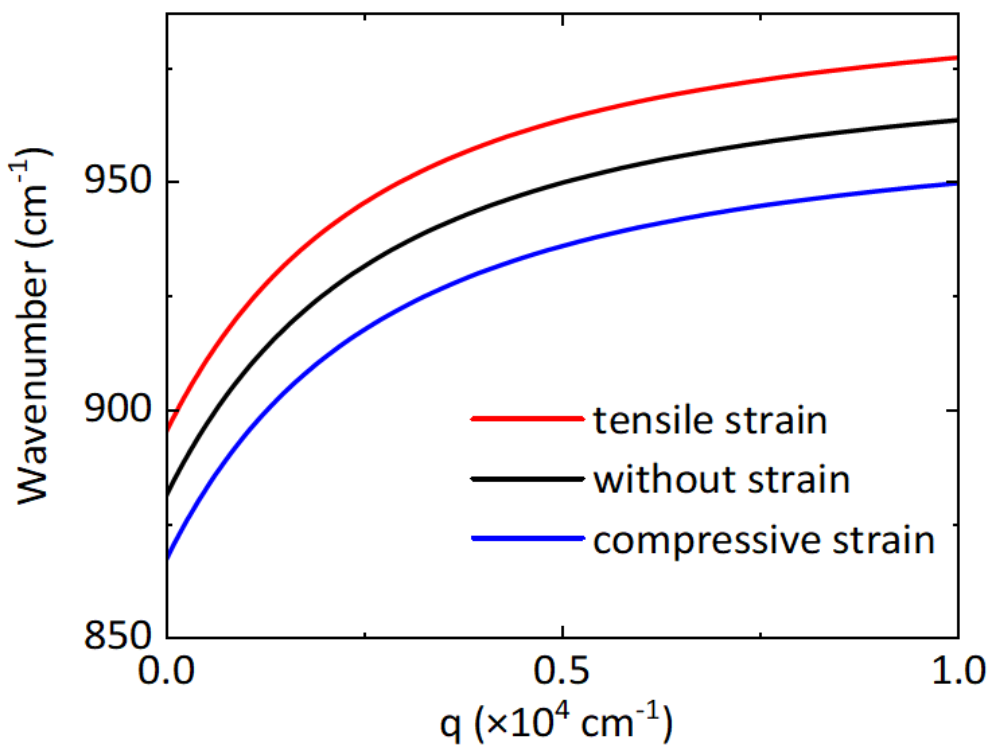

Figure R18. Calculated dispersion curves (frequency vs. in-plane wavevector) with (or without) tensile and compressive strain.

We are deeply grateful to the reviewer for the valuable technical guidance. The suggested approach further reinforces our conclusions, and we have cited the recommended references in the revised manuscript to acknowledge this important framework. We believe that the combination of DFT, near-field imaging, and Raman spectroscopy provides a robust and cross-validated determination of the permittivity tensor and the polaritonic nature of AMO.

Comment 4: On page 13, line 232, reference 29 seems to be misplaced, because the cited paper describes other types of polaritons, surface polaritons. The authors probably wanted to mention uniaxial hyperbolic material types, described, e.g., in Nat Commun 5, 5221 (2014). Furthermore, since the material is obviously not uniaxial, this classification should be used with caution and with appropriate comments.

Our Response: We thank the reviewer for this careful reading and for the valuable guidance on both the reference and the terminology. We acknowledge that reference 29 (which discussed surface polaritons) was indeed inappropriate in this context. Our intention was to cite a work that discusses type-II hyperbolic phonon polaritons. Following the reviewer's suggestion, we have replaced it with the recommended references (*Nature Communications* 2014, 5, 5221; J. Duan, Advanced Materials 2022, 34, 2104954). These works provide comprehensive discussions of hyperbolic phonon polaritons in hexagonal boron nitride, and are now correctly cited as reference [34] and [35].

To address the reviewer's concern about the "*uniaxial*", we would like to clarify a subtle point regarding the crystallographic equivalence of the two orthogonal edges of our square-like AMO nanoflakes. In the absence of external perturbation, these two directions are crystallographically equivalent. In the revised main text, we describe AMO crystal as a biaxial crystal that, under epitaxial strain, exhibits strain-induced in-plane anisotropy.

Comment 5: On page 13, line 223, the observed modes are called edge modes; however, these are not edge modes, but in-plane modes excited by the edge. Edge modes are modes that propagate along the edge, see, for example, Nano Lett. 2017, 17, 1, 228–235.

Our response: We thank the reviewer for this important clarification. We have carefully consulted the recommended references [Nature Photon. 2016, 10, 239–243; Nano Lett. 2017, 17, 5285–5290] and fully agree with the distinction drawn by the reviewer.

The fringe patterns observed in Figure **4b** arise from edge-launched phonon polariton rather than true "*edge modes*" (i.e., hyperbolic surface polaritons that are

intrinsically confined to and propagate along the edge). All instances of "*edge modes*" in the main text and figure captions have been replaced with "*edge-launched PhPs*".

Comment 6: Fringes, observed in Fig. 4, are not resonant peaks (page 13, line 230) because they do not correspond to any resonances; they are just interference maxima formed by the edge-launch polaritons fields and by the field of the tip.

Our Response: We thank the reviewer for this correction. We fully agree with the reviewer's assessment and have revised the text to accurately describe these features. The original sentence " *The spectra show a series of distance- and energy-dependent resonance peaks*" has been revised as *"The resulting spectra exhibit a series of distance- and energy-dependent fringe maxima, which arise from interference between the polariton field launched by the tip (subsequently reflected from the edge) and the background field at the tip*".

Comment 7: The use of the term "two-dimensional" in the context of the resulting flakes can be misleading. Flakes thicker than 100 nm are far from being monoatomic layers and are not two-dimensional for most of the physical processes occurring within them.

Our response: We thank the reviewer for this terminological clarification. We agree that in the context of nanophotonics and polaritonics, the term "*two-dimensional (2D)*" is conventionally reserved for ultrathin van der Waals materials with atomic-scale thickness (e.g., monolayer or few-layer graphene, hBN, or transition metal dichalcogenides). Our AMO flakes, with thicknesses ranging from 50 to 500 nm, do not fall into this category and should not be described as "*2D materials*". In response, we

have carefully revised the manuscript to replace all instances of "*2D*" with more accurate terminology. Specifically, "*2D AMO flakes*" has been replaced with "*thin AMO flakes*" or "*thin-layer AMO crystals*" throughout the manuscript.

We believe these revisions more accurately reflect the geometry of our samples without misusing the established terminology for true two-dimensional materials. The changes have been implemented throughout the manuscript and are marked for the reviewer's convenience.

Response Letter for No. NCOMMS-25-100045A

Dear editors,

We thank you and all reviewers for the positive evaluation of our revised manuscript (No. NCOMMS-25-100045A, entitled "Interfacial Epitaxy of Single-crystalline $Al_2(MoO_4)_3$ Flakes for Anisotropic Phonon Polaritons"). We also appreciate the constructive comments and suggestions provided during the previous review round, which have significantly improved the quality of our work. We are grateful for the reviewers' recommendation to accept the manuscript.

In this second revision, we have focused on addressing the remaining points raised by Reviewer #1, as well as the editorial requests. Below we provide a point-by-point response to Reviewer #1's comments. All changes are highlighted in yellow in the revised manuscript.

For Reviewer #1:

Reviewer #1: The authors have addressed in essence all the concerns I first expressed about the manuscript and its conclusions. Most essential is the convincing presentation of the identified polaritons as hyperbolic in nature.

There are in my view two remaining important issues to address. With these substantial modifications, I believe the work could deserve interest by the broad readership of Nature Communications.

Comment 1: First, the authors' brief added discussion of higher order polaritons perhaps identifiable as short-wavelength excitations near crystal edges is important and, therefore, perhaps too brief. The authors should make an effort to identify the wavelength of these putative high-order modes, for at least a few energies, so that they can be added to the authors' theoretical hyperbolic dispersion. The presented theoretical permittivity tensor makes possible to predict the wavevector of l=1 modes

which are presumably detectable in the experiment. Notably, the separation of l=0 and l=1 mode momenta is a direct reporter of "psi", which is an important figure of merit for hyperbolicity in connection to the angle of polariton ray propagation. With such expanded quantitative discussion of these higher order modes, and their importance for hyperbolic "beaming", the hyperbolic claims of the manuscript will be adequately substantiated.

Our response: We greatly appreciate your insightful comment regarding the importance of higher-order polariton modes and their direct connection to the hyperbolic figure of merit ψ. Given that the thickness of AMO flake shown in Fig. 5 (d = 270 nm) is different from the flake used in Fig. 4 (d = 470 nm), a direct addition of the experimental data points from Fig. 5 onto the dispersion plot in Fig. 4 would be physically inappropriate. To address this, we recalculated the hyperbolic dispersion relation specifically for the thickness of the AMO nanoplate examined in Fig. 5, and then added the experimentally extracted higher-order mode momenta to the calculated dispersion.

Following this strategy, we have now quantitatively analyzed the short-wavelength fringes near the crystal edges and performed the following additional analysis:

- Thickness-specific theoretical dispersion: Using the measured thickness of the AMO nanoplate in Fig. 4 and Fig. 5, we computed the polariton dispersion curves for the l = 0, 1, and 2 branches with our theoretically/experimentally determined permittivity tensor. As illustrated in Figure **R1**, the l = 0, 1, and 2 dispersion branches differ markedly between the 470 nm-thick and 270 nm-thick flakes. Notably, the calculated dispersion curves for the AMO crystal with thickness d = 270 nm by numerical simulations in Figure R1b, demonstrate good agreement with the analytical solution (dashed curves).

- From the high-resolution near-field images in Fig. 5, we selected two representative energies (ω = 930 cm^{-1} and 940 cm^{-1}) and performed spatial Fourier analysis of the interference fringes near the crystal edge, following the procedure described in previous work (Fig. 5g–h, S. Dai, et. al. Nat Commun 2015, 6, 6963). The wavelengths extracted from the short-wavelength fringes near the crystal edges at 930, 940 cm^{-1} are λp (l=1) ≈ 360 nm, 275 nm, respectively. These values yielded the in-plane momenta for the l = 1 modes. The experimentally extracted data points (marked with the blue sphere) are now superimposed on the thickness-matched theoretical dispersion. As shown in Figure R1c, the experimental points agree well with the higher-order branch (l = 0) of the calculated polariton dispersion based on our DFT permittivity tensor.
- Although we successfully extracted experimental data points corresponding to the higher-order l = 1 mode from the near-field images in Fig. 5, we were unable to obtain the l = 0 mode at the same wavevectors due to the strong confinement of phonon polaritons in the small-size AMO nanoplate. This confinement effect, arising from the limited lateral dimensions of the flake, suppresses the formation of well-defined low-order standing waves. Consequently, we instead used the theoretical separation between the l = 0 and l = 1 dispersion branches to calculate the hyperbolic figure of merit ψ. Namely, momentum separation $\Delta q = q(l{=}1) - q(l{=}0) = \pi\psi/d$. Alternatively, we can use the permittivity tensor to obtain the important parameter $\psi = -i\sqrt{\varepsilon_{\parallel} / \varepsilon_{\perp}}$. This figure of merit, which corresponds to the ray propagation angle, could help to understand the hyperbolic "beaming" character of the polaritons.

We have updated the main text to clearly state that the theoretical dispersion used for the higher-order mode analysis was calculated specifically for the thickness of the AMO nanoplate shown in Fig. 5 (270 nm), and that the experimental data points

were obtained from the edge-fringe analysis of the same sample. This treatment strictly follows the rigorous precedent established in previous h-BN studies (Dai et al., *Nat. Commun.* 2015, 6, 6963; *Science* 2014, 343, 1125). The extended discussion of the higher-order fringes has been added to the main text on page 13, lines 263–273, and the corresponding thickness-matched dispersion together with the experimentally extracted l = 1 mode momenta is presented in Supplementary Figure 11 (originally Figure **R1**). We believe these additions adequately substantiate the hyperbolic claims of the manuscript.

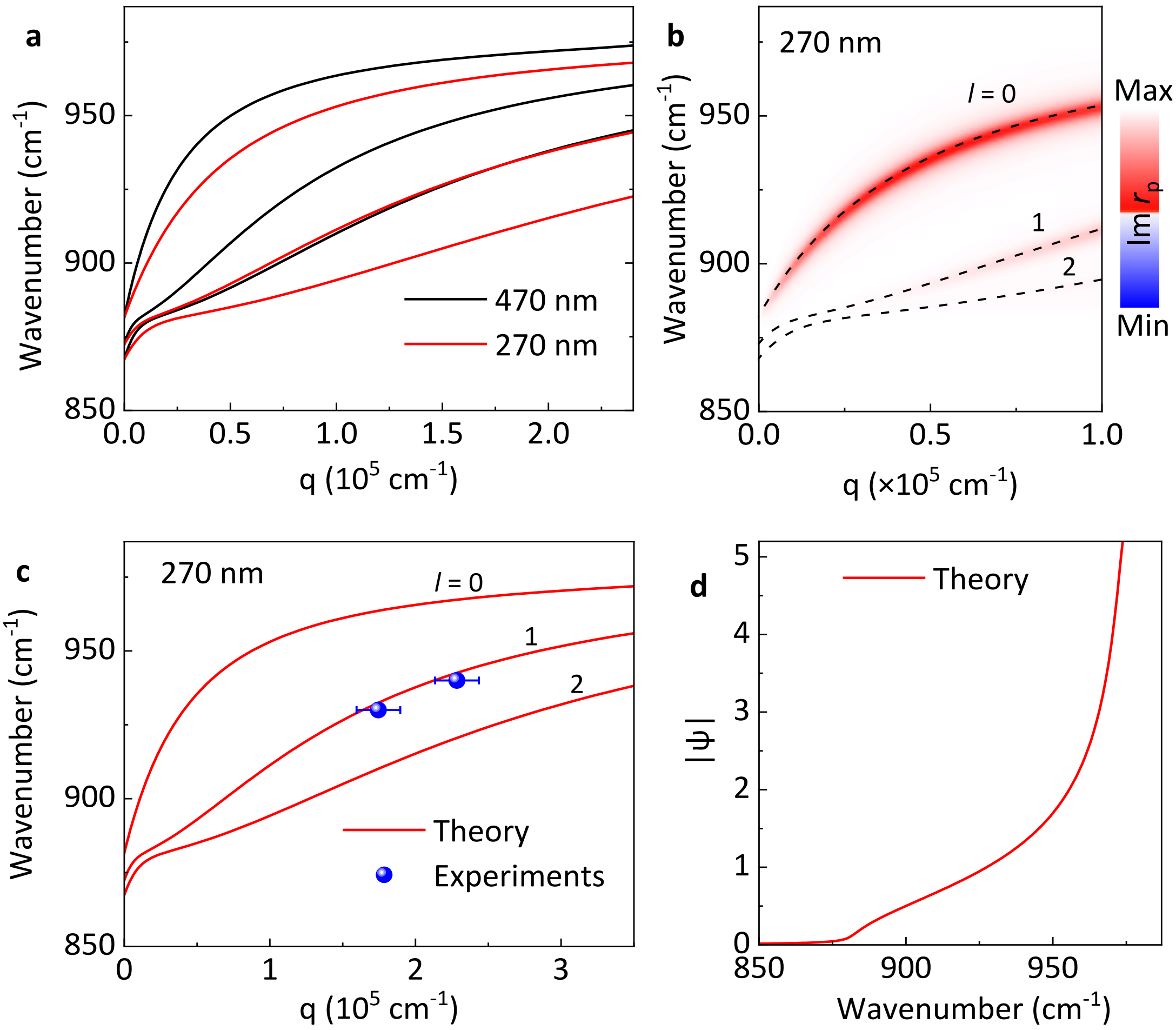

Figure R1. (a) Calculated dispersion curves of AMO crystal with thickness d = 470 nm (black) and d = 270 nm (red) by numerical simulations. (b) Numerical (solid) vs. analytical (dashed) dispersion curves of the 270-nm-thick AMO crystal for l = 0, 1, 2 modes. The close agreement confirms the validity of the analytical polariton model.

(c) Experimentally extracted l = 1 mode momenta (blue spheres) at 930 cm^{-1} and 940 cm^{-1} superimposed on the theoretical dispersion curves (solid) of the 270 nm-thick AMO nanoplate. (d) Theoretical hyperbolic figure of merit ψ as a function of wavenumber.

Comment 2: Second, the authors should expand their discussion of the unique merits of their epitaxial hyperbolic medium, in contrast to exfoliated hyperbolic crystals. The tabular comparisons to alternate materials are useful, but leave unaddressed the technological need for and progress towards large-area single-crystalline media. What are the foreseeable prospects for large-area single crystalline growth of this hyperbolic medium, especially in comparison to ongoing efforts towards CVD growth of e.g. hBN? Could large-area single crystalline sheets be obtained, and by what modified synthesis approach? Given the authors' new experience with materials transfer, what are the prospects and potential solutions for transfer of this medium to alternate optical substrates? Addressing these questions will better establish the significance of this new epitaxial hyperbolic medium.

Our response: We thank the reviewer for raising the important concerns about scalability and transferability of the AMO crystals. We fully agree that the unique merits of our epitaxial AMO will be better established once large-area single-crystalline growth and substrate transfer become feasible. Below we outline possible strategies to address both challenges.

1. Towards large-area single-crystalline AMO

Our current interfacial epitaxy yields AMO crystals with lateral dimensions of several tens of micrometers, which are sufficient for fundamental polariton studies. To scale the crystal size up to millimeter or centimeter scale, we propose:

- Increasing the nucleation site density. We propose to pretreat the sapphire substrate with Ar or O_2 plasma bombardment. This process can create a high density of surface defects (step edges, vacancies, and local disorder) that may act as preferential nucleation centers for AMO, thereby enhancing the areal density of single-crystalline domains without disturbing the confined-geometry epitaxy.
- Introducing pre-deposited α-Al_2O_3 seed layers with a high density of low-index facets to promote domain coarsening and the formation of larger single-crystalline AMO crystals.
- Optimizing growth parameters (temperature, time) to favor lateral expansion over vertical thickening, analogous to 2D growth regimes in van der Waals epitaxy.

2. To directly compare with CVD growth of hBN, our AMO interfacial epitaxy operates at a much lower temperature (≈700 °C vs. >1000 °C for *h*-BN) and does not require hazardous H_2/N_2 atmospheres or high-pressure conditions. This simpler, safer, and more energy-efficient process, combined with the plasma-enhanced nucleation strategy, positions AMO as a practical alternative for large-area single-crystalline hyperbolic media.

3. Transfer to alternative optical substrates

We recognize that the native sapphire substrate may not be optimal for all nanophotonic platforms. To enable clean transfer, we propose replacing sapphire with water-soluble $Sr_4Al_2O_7$ (SAO) single-crystal wafers. SAO contains Al^{3+} and can therefore participate in the same interfacial reaction that forms AMO. After growth, the SAO substrate can be dissolved in deionized water, releasing a freestanding AMO membrane that can be transferred onto arbitrary target substrates (SiO_2/Si, CaF_2, metals, or flexible polymers). This approach has been successfully demonstrated for perovskite oxide membranes and is directly adaptable to our confined-geometry

growth.

With the above strategies—dense nucleation sites and SAO-based sacrificial substrates—our interfacial epitaxy method can evolve into a scalable, transferable platform for hyperbolic AMO membranes, complementing exfoliated and CVD-grown *h*-BN. The manuscript has been revised accordingly, on page 15, lines 299–304, we wrote "To achieve large-area single-crystalline AMO, plasma-induced surface defects on sapphire could be used to regulate the nucleation density and lateral expansion of AMO domains, while transfer to arbitrary substrates could become feasible by using water-soluble $Sr_4Al_2O_7$ (SAO) as a sacrificial growth substrate. These strategies may pave the way for scalable, integrable AMO membranes in future investigations".
